# Identifying the proton donor in electrocatalytic hydrogen evolution by a model hydrogenase

**DOI:** 10.1007/s00775-026-02166-9

**Published:** 2026-08-04

**Authors:** Riley E. Stein, Ashlee E. Wertz, Peter J. Moore, Hannah S. Shafaat

**Affiliations:** 1 Department of Chemistry and Biochemistry, The Ohio State University, Columbus, OH 43210 USA; 2 Department of Chemistry and Biochemistry, University of California, Los Angeles, Los Angeles, CA 90095 USA

## Abstract

Proton transfer is essential in all multi-electron transformations. Identifying the proton donor in such processes is crucial to understanding the catalytic mechanism and optimizing activity through pK_a_ matching, which can enhance proton transfer rates. However, aqueous solutions offer multiple proton sources, which confounds clear identification of the catalytically relevant proton donor. In this work, we utilize a model hydrogenase, nickel-substituted rubredoxin (NiRd), which is capable of electrocatalytic hydrogen evolution. Prior mechanistic studies suggested a proton transfer process was involved in the rate determining step on the basis of isotopic substitution and kinetic modeling. In this study, the identity of the proton donor in this rate-determining step was investigated by employing a range of buffers with varied pK_a_ values. The impacts of concentration and pH on catalytic currents and turnover frequencies (TOF) were investigated. These results suggest that intermolecular, buffer-dependent proton transfer is not the rate-determining step for catalysis under typical electrocatalytic conditions. Quantitative electrochemical simulations incorporating buffer information allowed extraction of intrinsic thermodynamic and kinetic parameters for catalysis. Ultimately, mechanistic distinctions between the NiRd system and other hydrogen-evolving catalysts suggest that controlling the proton donor may serve as a handle for modulating activity in an intentional manner.

**Supplementary Information:**

The online version contains supplementary material available at https://doi.org/10.1007/s00775-026-02166-9.

## Introduction

Hydrogen offers a sustainable, renewable alternative energy carrier to traditional carbon-based fuels, especially for its ability to be generated from water [1]. However, current industrial means of H_2_ production utilize harsh conditions and generate CO_2_, motivating the development of more sustainable methods [1,2]. Enzymes as biocatalysts offer an attractive alternative approach for numerous reasons, including specificity, high rates, and the ability to catalyze reactions in ambient, aqueous conditions. In nature, hydrogenases have evolved for the efficient production of hydrogen gas from water using Earth-abundant transition metals, such as iron and nickel [3–5]. Despite the distinct advantages of hydrogenases as biocatalysts, several factors limit their large-scale usage, including their complex biosynthetic routes, sensitivity to oxygen, large size, and requisite coupling into metabolic pathways [6–9]. To harness the enhanced activity imparted by the protein fold, replicating enzyme active sites in simpler protein scaffolds to create artificial metalloenzymes offers the potential ability to better mimic structure and activity than molecular complexes [10,11].

**Fig. 1:**
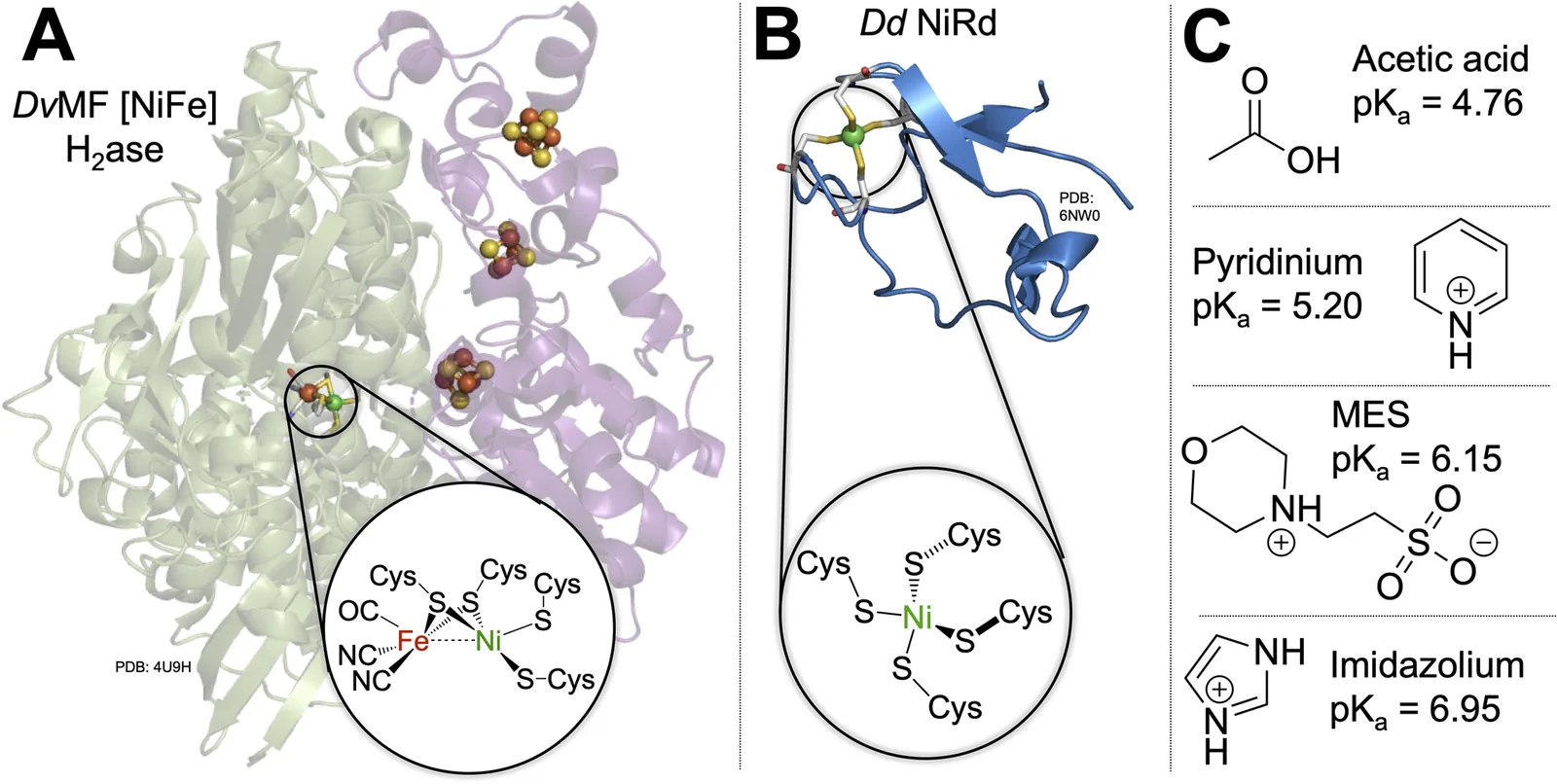
Cartoon structure and ChemDraw representation of the active sites of (**A**) *Dv*MF [NiFe] hydrogenase (PDB: 4U9H) and (**B**) *Dd* nickel-substituted rubredoxin (PDB: 6NW0). (**C**) Structures and pK_a_ values of the proton donors used in this study

Rubredoxins (Rds) are small electron transport proteins that natively bind iron (FeRd) in a tetrathiolate coordination. It has been previously demonstrated that the metal-binding site can be easily substituted with a variety of first row transitions metals, including nickel [12–14] The nickel-substituted rubredoxin (NiRd) thus closely resembles the redox-active metal site of the [NiFe] hydrogenase, where only the nickel undergoes changes in oxidation state throughout the catalytic cycle (Fig. 1) [3–5] Previous work by our group has demonstrated that in addition to being a structural mimic, NiRd serves as both a functional and mechanistic model of the [NiFe] hydrogenases [15–18] Moreover, NiRd offers several benefits over the native system. The rubredoxin scaffold is robust to a broad range of pH and temperatures and is notably insensitive to oxygen. These factors establish NiRd as an ideal platform for investigating molecular factors affecting catalysis.

The proton transfer process can have profound effects on catalysis, especially in biological systems [19,20]. However, in aqueous media, it is often difficult to evaluate the proton donor, as there can be multiple proton sources, including the solvent itself. Elucidating the proton donor is crucial for understanding the catalytic mechanism and optimizing activity. Prior work by the Bren lab and others have established that the buffers in solution can play an active role in proton transfer for many different systems [21–23]. In a synthetic CO_2_-reducing system, the buffer was found to directly modulate the activity and selectivity through binding to the nickel active site [24]. Buffer considerations have also been examined for an artificial metalloenzyme that catalyzes hydrogen evolution, where buffer pK_a_ was shown to impact the catalytic mechanism and buffer sterics impacted the activity [22]. Buffer participation is also seen in natural systems. An iconic example is the role of buffer in carbonic anhydrase, where a buffer molecule transfers protons to a residue near the active site, and at low concentrations of buffer, this proton transfer becomes rate-limiting [25,26].

Motivated by prior findings that buffer effects can impact catalysis, we sought to understand the role of buffer in the activity of NiRd. The mechanism of the hydrogen evolution reaction (HER) of NiRd has been found to involve a rate-determining proton transfer step, but the source of this proton remained unidentified (e.g., buffer, solvent, an intramolecular source). To resolve this and explore opportunities to use buffering molecules to enhance catalysis, the electrocatalytic behavior of NiRd was assessed across a suite of buffers as proton donors through protein film electrochemistry (PFE). Both cationic and neutral proton donors along with varied proton donor functional group were investigated. Prior work on other catalysts suggested that buffer charge could modulate selectivity for CO_2_ reduction, implicating a role for the proton donor in the rate-determining step [27]. In this case, however, the catalytic activity of NiRd was only slightly impacted by varying the concentration over multiple orders of magnitude and across different pH values. Quantitative electrochemical modeling incorporating explicit buffer effects was used to extract fundamental catalytic parameters. These results suggest that intermolecular proton transfer is not the rate-determining step for hydrogen evolution by NiRd, in contrast to what has been proposed for other artificial metalloenzymes that catalyze aqueous HER, [21, 22, 28] which offers design guidelines for targeted catalyst optimization. This general approach can be applied across diverse catalytic systems, with the goal of assessing key aspects of proton transfer in aqueous solutions.

## Materials and methods

### Protein expression, purification, and metalation

*Desulfovibrio desulfuricans* ATCC 27,774 wild-type rubredoxin (Rd) was heterologously expressed, purified, and metalated as previously described [15,16,29]. Purity was assessed with native gel electrophoresis following metalation.

### Electrochemistry

Covalent coupling of Rd to the surface of freshly polished homemade pyrolytic graphite (PG) electrodes was conducted following the procedure previously outlined [17]. Homemade PG electrodes were made using the same materials and procedure detailed previously [17]. Experiments were repeated at least three times as independent biochemical replicates with different coupled protein films (Figures S1 – S25).

All electrochemistry was conducted in a nitrogen glovebox ([O_2_] < 3 ppm; Vigor Technologies and MBRAUN) using a CH Instruments 760E bipotentiostat. All electrochemistry was performed with a three-electrode system consisting of a platinum wire counter electrode (Alfa-Aesar), homemade PG (edge-plane) working electrode, and an Ag/AgCl reference wire (KCl saturated; Pine Research Instrumentation). All reported potentials were converted to the normal hydrogen electrode (NHE) potentials by adding 0.197 to collected potentials vs. Ag/AgCl. Electrodes were scanned at 25, 50, 100, 200, 500, and 1000 mV/s twice, and the second scan was used for data analysis. Electrochemical analysis of noncatalytic signals was performed in qSOAS, a software package for electrochemical data analysis [30,31]. The noncatalytic signal was integrated (I_int_), and the average of the cathodic and anodic signal was related to electroactive coverage (Γ_FeRd_) through Eq. 1

1 $${{\rm{I}}_{{\rm{int}}}}{\rm{ = Fv}}{{\rm{\Gamma }}_{{\rm{FeRd}}}}$$

where F is Faraday’s constant, and v is the scan rate.

Experiments were performed in either acetate (VWR Life Science), pyridine (Spectrum Chemical MFG Corp.), 2-(N-morpholino)ethanesulfonic acid (MES; Goldbio), or imidazole (Alfa-Aesar) in 100 mM KCl (VWR Life Science). Solutions were prepared by dissolving solids in deionized water (18.2 MΩ⋅cm). To adjust the pH, an InLab^®^ Expert Pro-ISM pH probe (Mettler-Toledo) was calibrated before measurements with calibration buffers of 2.0, 4.0, 7.0, and 10.0 (VWR) accurate to 0.01 pH units. Solutions were titrated with minimal amounts of either 1.0 M or 10 M solutions of HCl or KOH as necessary, diluted once at the desired pH to the appropriate volume, and the pH was rechecked to be within ± 0.01 pH unit of the desired pH. Solutions were then extensively sparged with N_2_ (1 min/mL solution) and then brought into a glovebox and allowed to equilibrate with the atmosphere for at least 24 h. Stochiometric amounts of buffer solution were prepared by adding an appropriate amount of volume from a 100 mM stock. The volume of the solution was brought close to the final volume (~ 98% totally final volume) and then adjusted with 1 mM stocks of HCl or KOH as needed. Solutions were then brought to volume and the pH was rechecked and adjusted as needed with less than 1% of the volume with 100 µM stocks of HCl or KOH as needed to ensure the appropriate pH.

Reported overpotentials were determined through Eq. 2:

2 $$\:{E}_{overpotential}=\:\left|{E}_{HER,\:\:\:pH}^{\circ\:{\prime\:}}-\:{E}_{HER,\:\:\:pH,\:\:\:NiRd}^{\circ\:{\prime\:}}\:\right|$$

where all potentials are in V. $\:{E}_{HER,\:\:\:pH}^{\circ\:{\prime\:}}$ is the thermodynamic potential of proton reduction at a given pH determined by the relationship − 0.059 V*pH, and $\:{E}_{HER,\:\:\:pH,\:\:\:NiRd}^{\circ\:{\prime\:}}$ is the onset potential of proton reduction by NiRd at a given pH determined as outlined below.

### Quantitative protein film electrochemistry

 Quantitative protein film electrochemistry was carried out as previously described [16,17]. All electrochemical experiments used a 1:1 mixture of NiRd and FeRd covalently attached to the electrode for turnover frequency (TOF) calculations. The electroactive coverage of FeRd was determined as previously described and the electroactive coverage of NiRd was assumed to be the same. Because of the lack of a plateau current for catalysis, the NiRd onset potential (E_onset_) was determined as the inflection point of the first derivative of the cathodic portion of the cyclic voltammogram (CV; Figure S26). The use of inflection points as a metric for reduction potential is also suggested for irreversible couples [32]. The capacitive current for all CVs was obtained by measuring the current at a point 100 mV more positive than the onset potential and subtracted accordingly. The catalytic current used for calculations ($\:{i}_{{E}_{analysis}}$) was collected at a point 100 mV more negative than the onset potential and the TOF was calculated with Eq. 3:

3 $$\:TOF=\frac{{i}_{{E}_{analysis}}}{{{\Gamma\:}}_{NiRd}nF}$$

where $\:{{\Gamma\:}}_{NiRd}$ is the electroactive coverage of NiRd, assumed to be the same as FeRd, * n* is the number of electrons transferred during the redox reaction (2 for proton reduction), and * F* is Faraday’s constant [17]. Current normalization was performed to account for film deactivation and achieved by taking the catalytic current of the reference solutions before and after the buffer concentration of interest ($\:{i}_{{E}_{analysis,\:\:\:100\:mM\:reference}}$) and averaging the two values. The catalytic current of the condition of interest was then divided by the reference catalytic current to obtain the normalized current and correspondingly reported as *i/i*_*100 mM*_.

## Results

### Buffer identity, charge, and concentration effects on catalysis

**Fig. 2:**
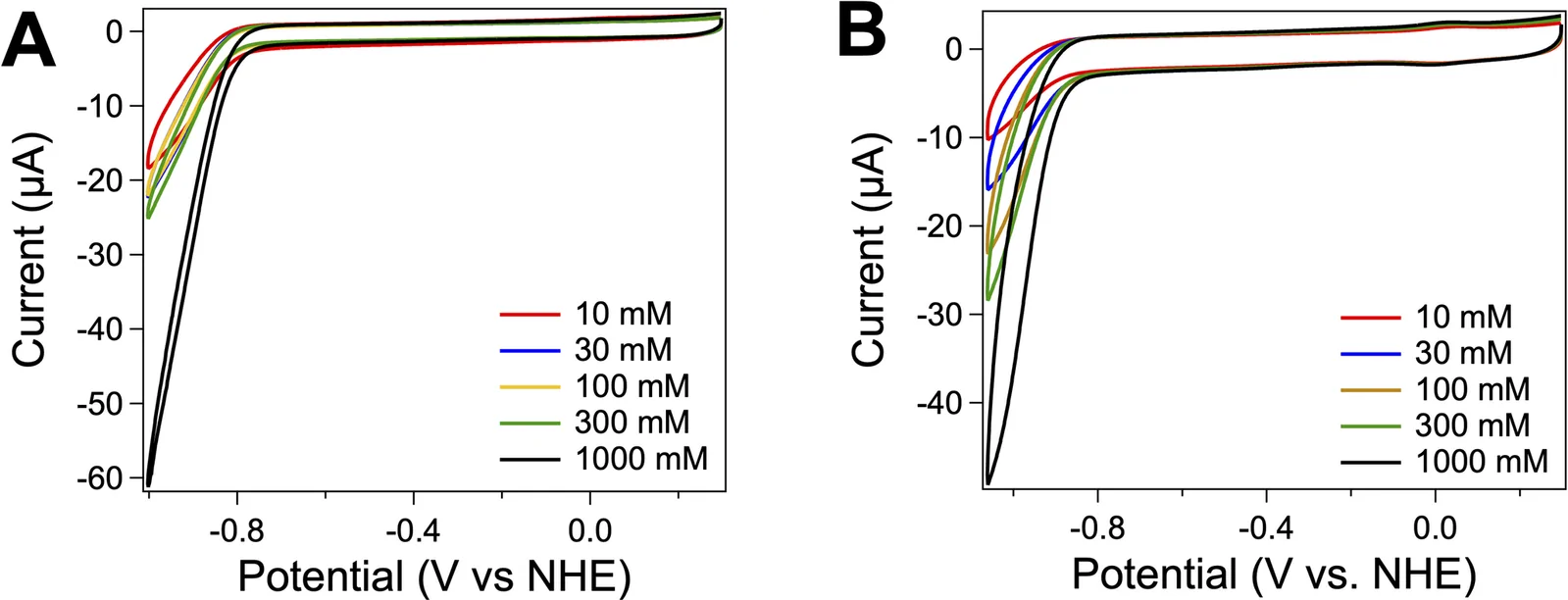
Cyclic voltammograms (ν = 25 mV/s) of 1:1 mixtures of NiRd and FeRd covalently coupled to PG electrodes measured in acetate buffer at (**A**) pH 4.5 and (**B**) pH 5.5 at the indicated concentrations. All buffer solutions included 100 mM KCl

The electrochemical characterization of both NiRd and FeRd has been previously conducted on pyrolytic graphite (PG) surfaces through several coupling methods in the CHAMP (*C*HES, *H*EPES, *a*cetate, *M*ES, *p*erchlorate) buffering electrolyte system [16,17]. In this work, we expand that characterization to probe the effects of varying the proton donor and use acetic acid, pyridinium, imidazolium, and MES buffers as proton donors. The protein is stable under all conditions tested, and even at high excess, no direct interaction between the buffering molecule and the nickel center is observed (Figure S27). The direct covalent attachment of the Rd scaffold to the intrinsic functional groups on the PG surface has demonstrated prolonged film stability, allowing a single film to be probed over long timescales [17]. In order to investigate the behavior of a single film in all investigated conditions, the covalent attachment method was used for all presented cyclic voltammograms. All films included a one-to-one mixture of FeRd and NiRd in order to use the quantitative protein film electrochemistry analysis developed previously [16,17].

**Fig. 3:**
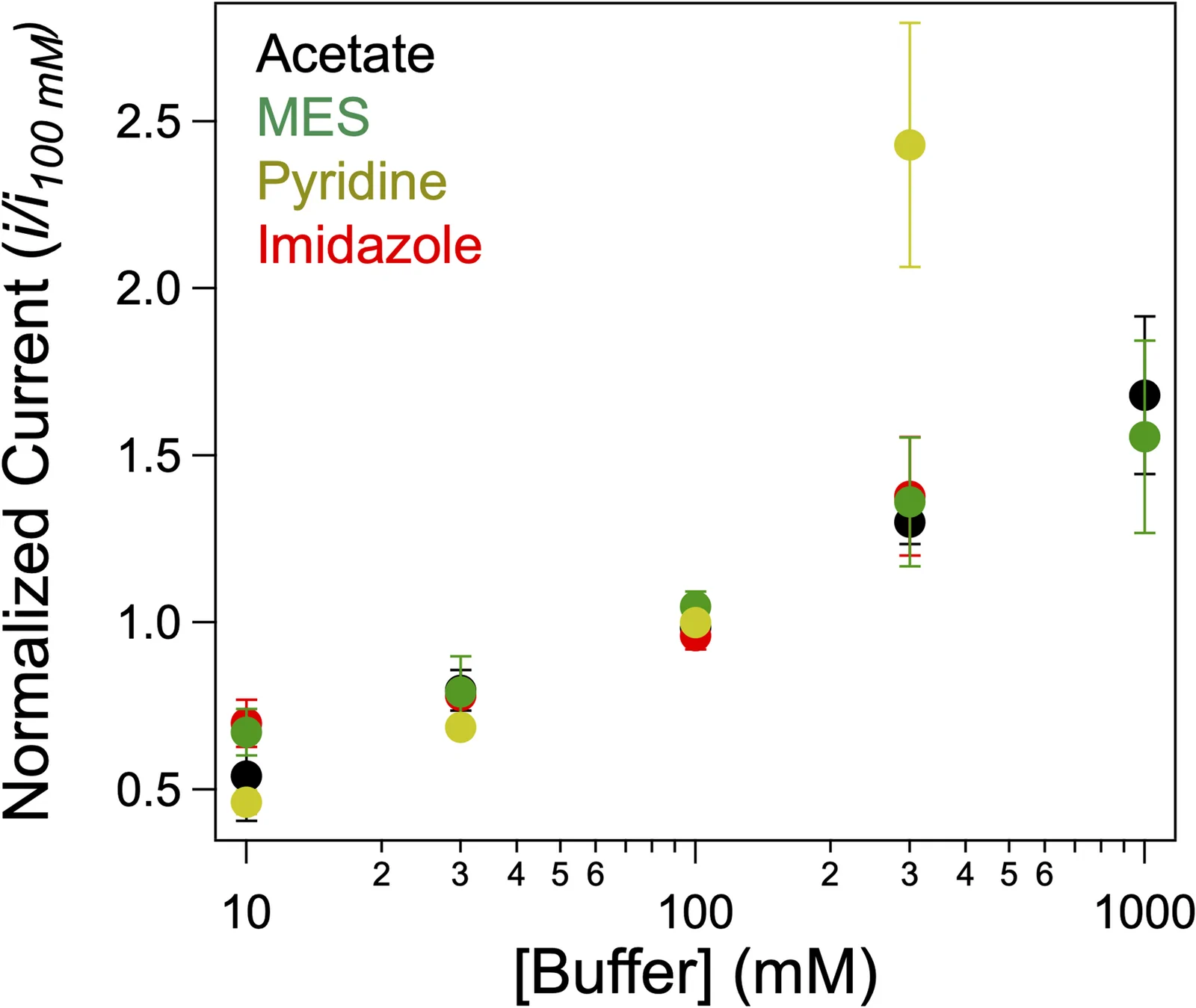
Average normalized current values of NiRd in acetate (*black*), MES (*green*), pyridine (*yellow*), and imidazole (*red*) buffers at pH 4.5 at the indicated buffer concentrations. All buffer solutions included 100 mM KCl. Currents were evaluated at 100 mV more negative than the onset potential of catalysis

As has been previously reported, a single reversible feature corresponding to the Fe^III^/Fe^II^ couple is observed around +38 mV vs. NHE, and an irreversible catalytic wave due to H_2_ evolution catalyzed by NiRd is observed with a modest overpotential of 564 mV.^16^ Representative cyclic voltammograms (CVs) of FeRd/NiRd in acetate buffer at pH 4.5 and pH 5.5 are presented in Figure 2. At both pH values, the catalytic current increases with buffer concentration. To quantitatively assess these trends, the normalized current is used as a point of comparison, where the current in the buffer of interest is compared to the current in a 100 mM reference solution taken before and after each scan in the buffer of interest. The normalized currents for every buffer investigated in this study are shown in Figure 3 for pH 4.5 and Figure S28 for pH 5.5. For all buffers, the normalized current increases modestly with buffer concentration, regardless of buffer identity, charge, pK_a_, or pH. These increases span only a factor of 2-3 across the 100-fold increase in buffer concentration, far lower than would be expected for a first-order dependence. For pyridine and imidazole buffers, the highest concentration used was 300 mM, because artifacts due to buffer redox processes were observed in both cases at 1 M, as reported previously (Figures S7-12, S19-S25) [33]. However, the electrochemical signals at lower (≤300 mM) buffer concentrations were characteristic of NiRd electrocatalytic behavior.

**Fig. 4:**
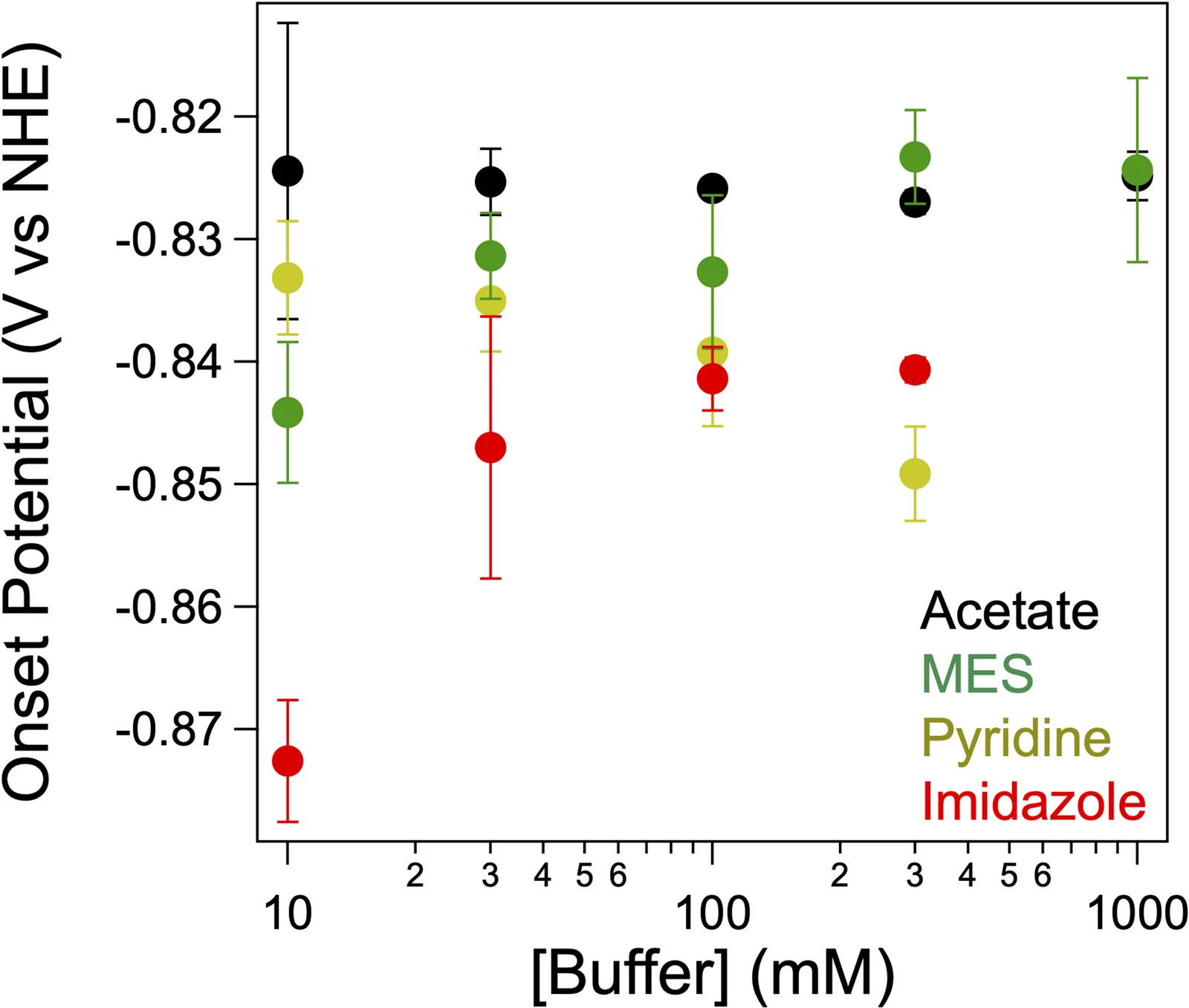
Catalytic onset potential of NiRd in acetate (*black*), pyridine (*yellow*), MES (*green*), and imidazole (*red*) at pH 4.5 at the respective buffer concentrations. Values shown represent the average across *n* ≥3 independent trials

The electrocatalytic onset potential reflects the kinetic barrier for entering the catalytic cycle and may also be affected by the proton donor, as previously seen for other artificial metalloenzyme catalysts [22]. Because the NiRd catalysis does not reach a limiting current value from which to obtain the onset potential, the onset potential is instead defined as the inflection point of the first derivative of the CV, as described previously [16]. All of the catalytic waves resulted in a sigmoid-shaped derivative, validating this approach under these conditions. Two trends are observed in onset potential as a result of varying buffer identity and concentration (Figure 4, Table S1). For the more acidic buffers, acetate (pK_a_ 4.76) and pyridine (pK_a_ 5.2), the onset potential is independent of buffer concentration, resulting in onset potentials of -826 ± 1 mV vs. NHE and -840 ± 7 mV vs. NHE, respectively. A slight increase in overpotential is even observed at 300 mM pyridine, though the catalytic signals at this concentration are convolved with background signals and thus have increased associated error (Figures S19-S21). For the more basic buffers, MES (pK_a_ 6.15) and imidazole (pK_a_ 6.95), there is a noticeable dependence of onset potential on buffer concentration. In MES, this dependence is modest. From 10 mM to 30 mM, the onset potential increases from -845 mV vs. NHE to -832 mV vs. NHE, but concentrations above this remain constant with an onset potential of -829 ± 8 mV vs. NHE. Imidazole induces a more dramatic, concentration-dependent change in onset potential. From 10 mM to 30 mM, the onset potential shifts from -874 mV vs. NHE to -848 mV vs. NHE. Like MES, above 30 mM, the onset potential is approximately invariant of buffer concentration, with an average of -844 ± 3 mV vs. NHE. At pH 5.5, the onset potential is independent of buffer concentration for all buffers (Figure S28). Acetate has an onset potential of -904 ± 9 mV vs. NHE; pyridine has an onset potential of -899 ± 6 mV vs. NHE; MES exhibits an onset potential of -885 ± 4 mV vs. NHE; and the onset potential in imidazole is -895 ± 4 mV vs. NHE. The slight differences in average onset potential at the higher pH values may reflect greater kinetic barriers for protonation, as discussed further below. The same trends are evident when considering only the concentration of protonated buffer molecule, or proton donor concentration (Figure S29).

**Fig. 5:**
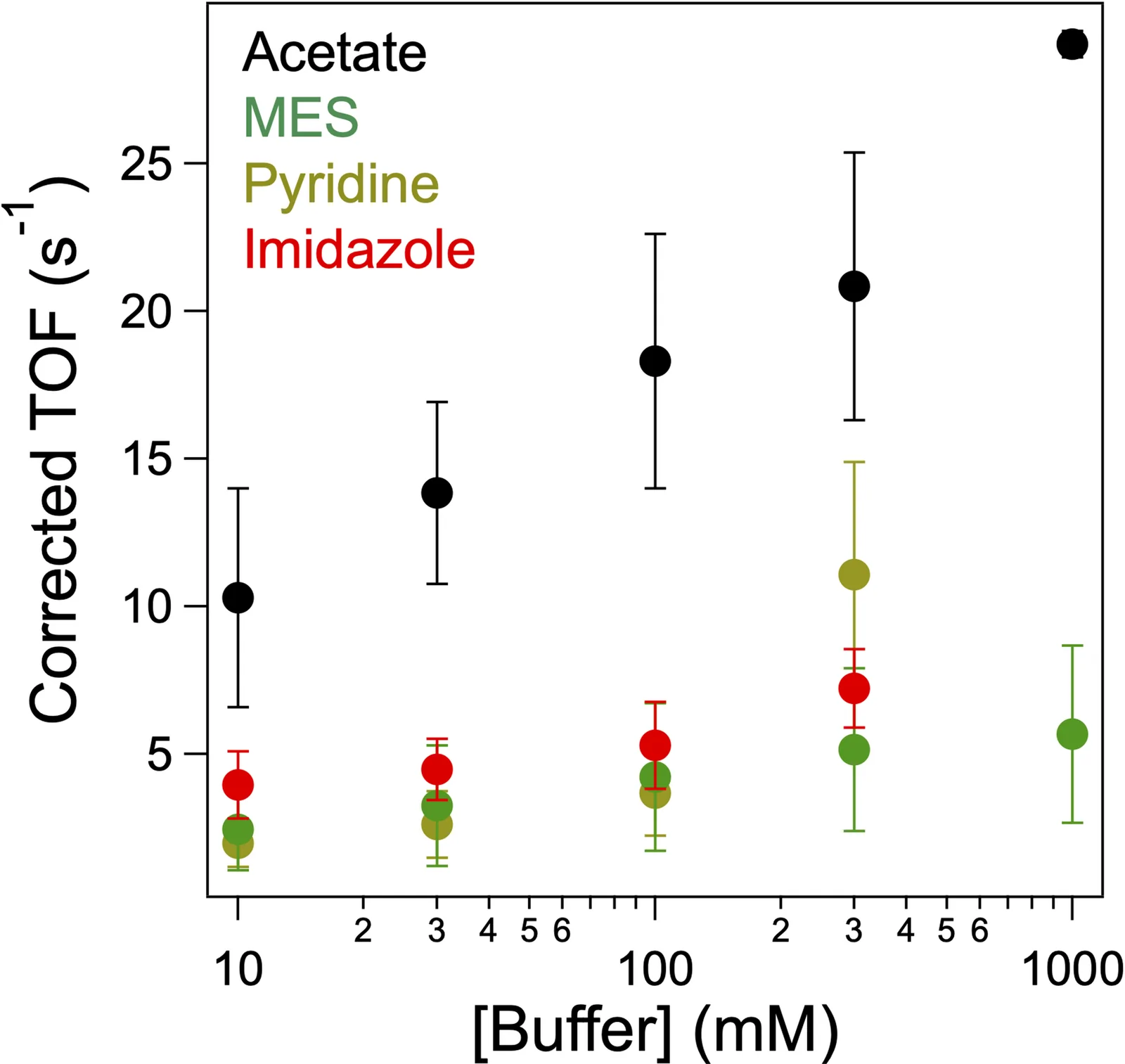
Average (*n*≥3) NiRd TOF values in acetate (*black*), pyridine (*yellow*), MES (*green*), and imidazole (*red*), obtained from qPFE analysis of 1:1 mixtures of NiRd and FeRd at pH 4.5 at the indicated concentrations

Turnover frequency (TOF) is another important parameter for assessing the intrinsic activity of catalysts and comparing rates between catalysts performing the same reaction. To further assess the trends of buffer on NiRd catalysis, the quantitative protein film electrochemistry (qPFE) method previously outlined was applied to quantify electrochemical TOFs (Figure 5) [16]. Additionally, a procedure from Fourmond et al. was adapted to further control for protein film deactivation and is detailed in the Supplementary Methods and Figures S30-S31 [34]. As expected from the current increases, the TOFs generally increase with increasing buffer concentration, with acetate showing the largest dependence. The lowest TOFs are observed for pyridine, suggesting the buffer pK_a_ does not drive this behavior, while a shallow monotonic increase is observed in MES and imidazole. The overall TOFs are comparable to those previously reported by Treviño et al., with a characteristic decrease in going from adsorbed protein films to a covalently attached system [17].

### Cyclic voltammograms of NiRd under limiting buffering conditions

**Fig. 6:**
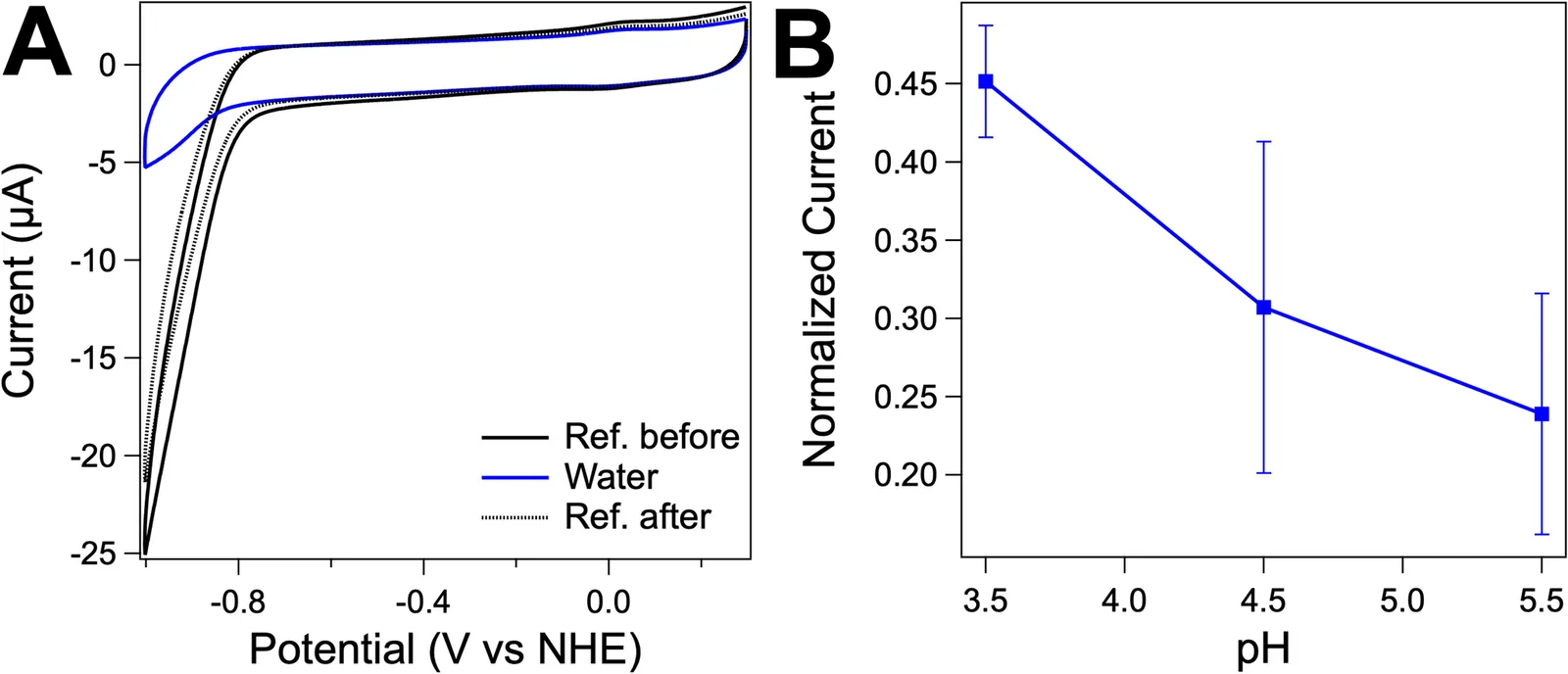
(**A**) CVs of 1:1 mixtures of NiRd and FeRd in unbuffered water (*blue*) shown with 100 mM acetate references measured before and after the experiment. Samples contained 100 mM KCl and were adjusted to pH 4.5. (**B**) Normalized catalytic currents for NiRd-catalyzed HER in unbuffered water at the indicated pH values

To elucidate the role that buffers play in modulating aqueous catalysis, it is useful to consider the catalytic properties in the absence of any buffer, i.e., in unbuffered water. NiRd is amenable to electrocatalytic experiments in unbuffered water, as its modest levels of activity are not expected to significantly alter the local pH environment during a CV. A representative CV of NiRd in unbuffered water compared to a buffered solution is presented in Fig. 6A along with the normalized current data, referenced to a buffered solution of 100 mM acetate at that pH value (Fig. 6B**)**. Because the signals at pH 4.5 were low but reproducible (Figure S32), we performed additional CVs at pH 3.5, where the signals more closely resembled those of NiRd in buffered solution (Figure S33); at pH 5.5 in unbuffered water, even lower catalytic signals were observed (Figure S34).

**Fig. 7:**
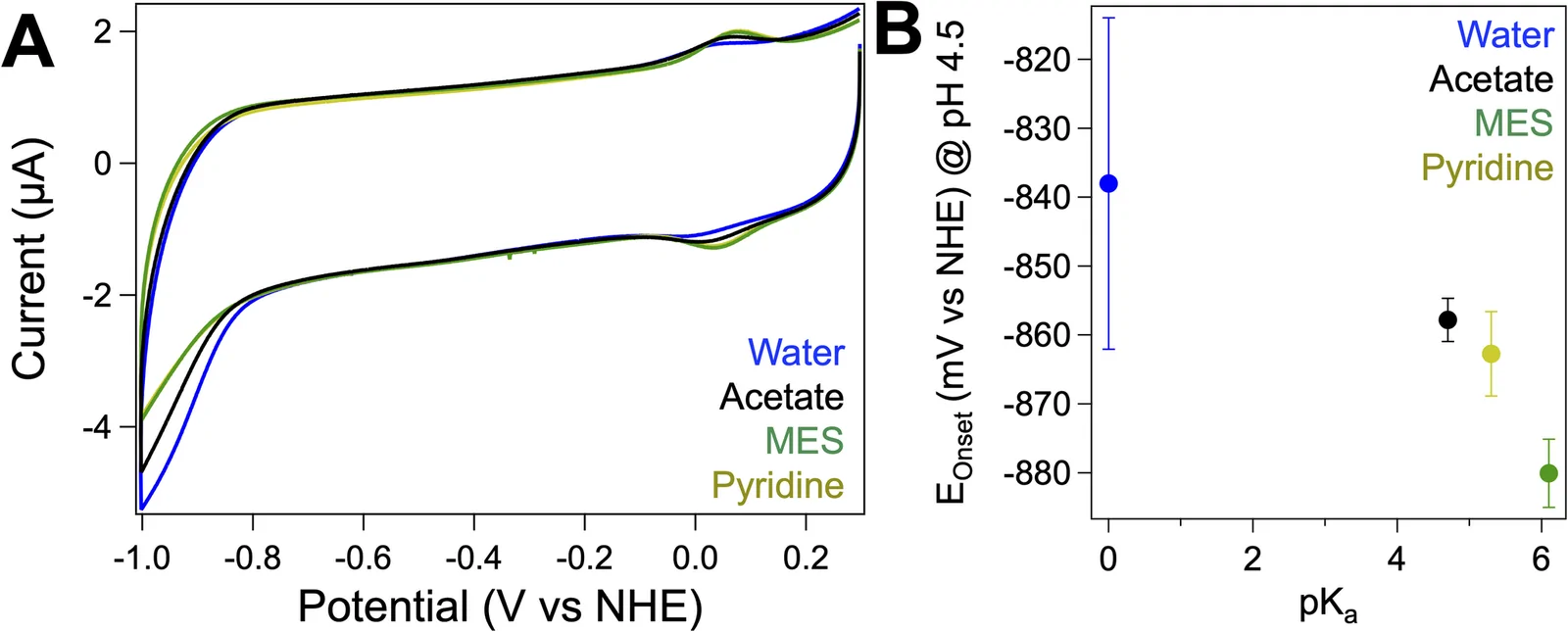
(**A**) Representative CVs of 1:1 mixtures of NiRd and FeRd in unbuffered water (*blue*), 31.6 µM acetate (*black*), 31.6 µM pyridine (*yellow*), and 31.6 µM MES (*green*) with 100 mM KCl at pH 4.5. CVs are scaled to correct for background electrode capacitance. (**B**) Catalytic onset potentials obtained from the CVs in (**A**) with unbuffered water (*blue*), acetate (*black*), pyridine (*yellow*), and MES (*green*) plotted as a function of proton donor pK_a_. The hydronium pK_a_ was used as this would be the relevant proton donating species under these conditions.

Unbuffered water gives lower currents and a slightly lower onset potential than the most dilute buffered solutions previously considered, suggesting that the hydronium ion is a less effective proton donor than the buffer. However, this experiment also changed the relative concentration as well as the identity of the proton donor. To account for this additional variation, experiments were performed using concentrations of buffer that are equivalent to the concentration of hydronium at pH 4.5, which is 31.6 µM (Fig. 7A). In these experiments, the onset potential for catalysis varies with buffer pKa, with the least-negative onset potential (lowest overpotential) observed for the lowest pK_a_ proton donor (Fig. 7B). This dependence is fairly shallow, though, with only a ~-6 mV/ pKa unit shift across the series. Even removing the contribution from hydronium, the shift is only ~ 16 mV/pKa unit. NiRd catalytic signal was not able to be resolved over background electrode signals using 31.6 µM imidazole as proton donor.

### Electrochemical simulations provide intrinsic catalytic parameters

**Fig. 8:**
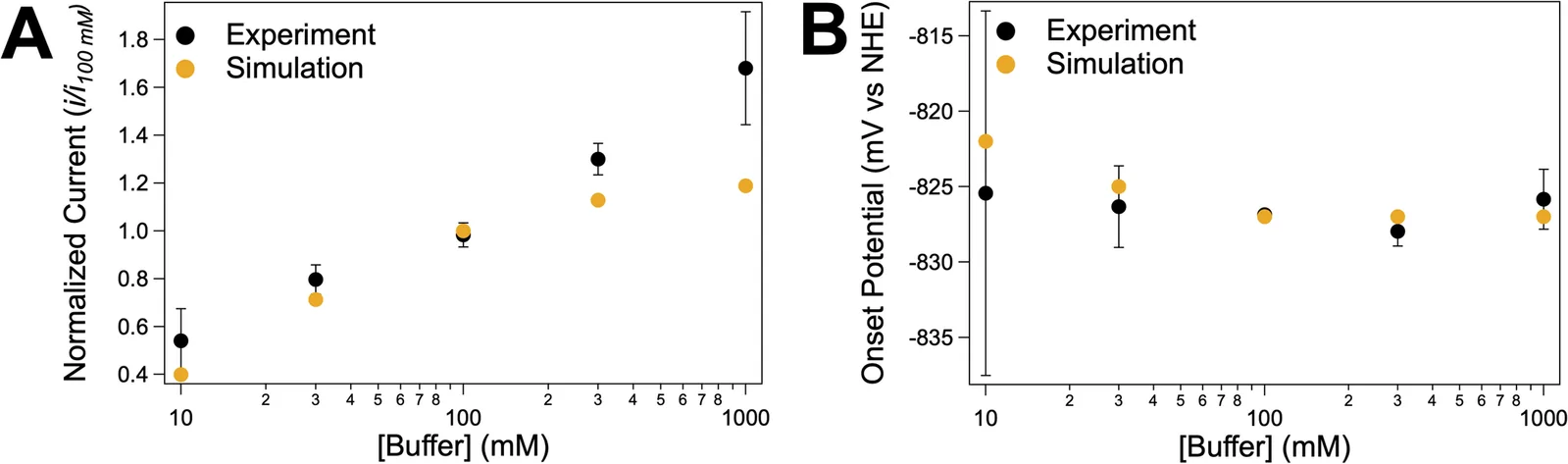
Experimental data (*black circles*) overlaid with simulated values (*orange circles*) of (**A**) NiRd normalized current and (**B**) NiRd onset potentials in acetate buffer at pH 4.5 at the indicated concentrations.

**Fig. 9:**
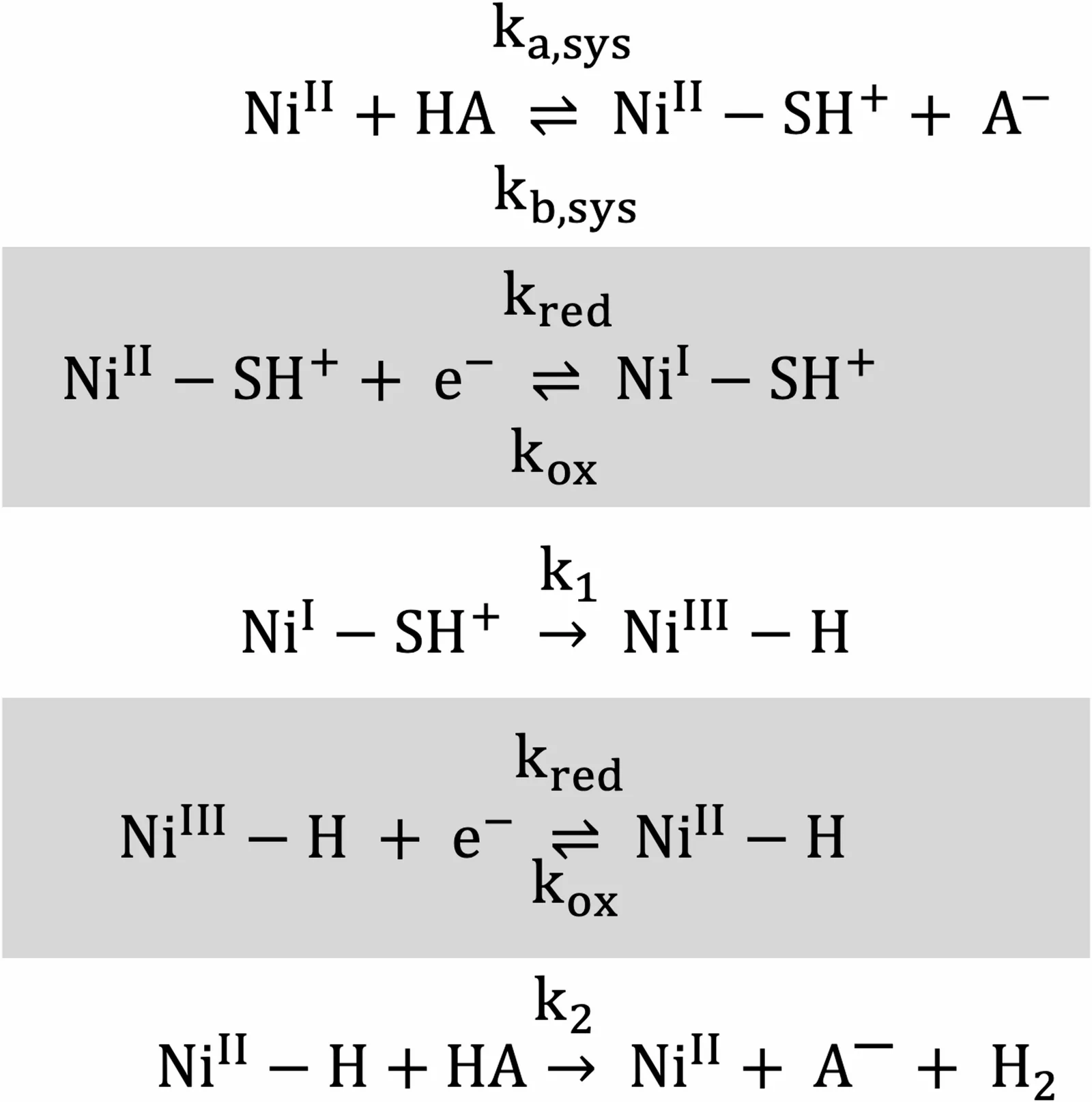
Proposed mechanism of hydrogen evolution by NiRd

Past experimental characterization of NiRd in conjunction with semi-quantitative voltammetric simulations suggested a mechanism similar to the native [NiFe] hydrogenase [16]. The previous electrocatalytic model invokes a CECEC pathway as the unique mechanism that can recapitulate the experimental observables; the four-step EECC, ECEC, CECE, and CEEC mechanisms fail to reproduce (i) the pH-dependent onset potentials; (ii) the pH-independent turnover frequencies; and (iii) the absence of a defined nickel-based one-electron couple [16]. This model assumed direct protonation of a coordinating thiolate from water, which successfully predicted the pH-dependent onset potential with a slope of 59 mV/pH unit and the nearly pH-independent TOFs for the adsorbed and covalently attached systems [17]. However, it does not account for the trends observed in this work when varying buffer concentration. To reconcile this, the model has been expanded, and the buffer was considered to be the proton donor, with explicit concentrations considered. A corresponding term reflecting the conjugate base (k_b_) and the buffer pK_a_ (k_a_/k_b_) was added to the elementary steps within the previously proposed mechanism, taking into account the estimated pK_a_ of the nickel(II) thiolate site. The direct protonation of NiRd by buffer results in rate and equilibrium constants for protonation and deprotonation that encompass the whole system pK_a_ (k_a, sys_/k_b, sys_) (Figure 9). This simple additional consideration reproduced the experimental trends in good agreement with the quantitative values (Fig. 8, **Figures S35-S38**,** and Appendix 1**).

The simulations constrain additional intrinsic catalytic parameters. While most of the parameters were found to be invariant across buffer identity, including the pK_a_ of the Ni(II)-bound thiolate and the intrinsic reduction potential of the system, some differences were resolved in k_a_, the forward rate constant for protonation of the NiRd system by buffer, and in k_1,_ the intramolecular proton-transfer and hydride formation step. In comparison to our previous model, where k_a_ was assumed to be diffusion-limited [16], the k_a_ values were obtained here by matching the observed shifts in onset potential (e.g., Figure 8B). By including a term accounting for the pK_a_ of the proton donor, which locks in the ratio k_b, sys_/k_a, sys_, the k_a, sys_ value can report on the rate of productive interaction between the proton donor and the nickel site. This value therefore encodes information about the protein environment accommodating the buffer molecule, which requires some rearrangement of the loops around the active site.^35^ The k_1_ value, which is the rate-determining step even under these conditions, could be constrained by reproducing the changes in normalized currents (or TOF values) as a function of buffer concentration and identity. By including a kinetic model that incorporates the proton donor, additional information on how buffer impacts the dynamics of catalysis can be obtained. The heuristically modeled best-fit k_1_ and k_a_ values are summarized in Table 1. The rates of interfacial electron transfer were constrained by Butler-Volmer theory adapted for a distribution of orientations, [36] with an intrinsic reduction potential of -751 mV vs. NHE. Because the second reduction process and second protonation step are not observed in the electrochemical experiments, they are modeled as fast relative to k_a, sys_, k_red_, and k_1_ values, as described earlier.^16^.

As observed qualitatively in Fig. 4, there is a distinction between buffers with low pK_a_ (acetate, pyridine) and high pK_a_ (MES, imidazole) values. In order to reproduce the only modest changes observed in onset potential for the high pK_a_ buffers, the rate of protonation must be modeled as approximately an order of magnitude faster; keeping the same k_a_ across all of the buffers results in significantly worse agreement between the simulation and the experimental data (Figures S39-S40). Similarly, to match the experimental turnover frequencies observed in each of the buffers, which only vary by a factor of ~ 5, the value for k_1_, the rate-determining chemical step, must also increase slightly for the high pK_a_ buffers. However, we note that these values are less constrained because of their minimal impact on onset potential, which is the experimental property that is most accurately known. The uniqueness of the modeled parameters is supported by experimental constraints on pK_a_ (Ni-SH^+^), which must fall below solution-accessible pH values and above 2 to match the pH dependence of the onset potential, as discussed previously (Figure S41).^16^.

**Table 1:** Modeled Kinetic Parameters for Each Buffer with CECEC Mechanism

Buffer	pK_a_	k_a_ (M^− 1^-s^− 1^)	k_1_ (s^− 1^)
Acetate	4.76	10^3^	15
Pyridine	5.2	10^3^	17
MES	6.15	10^4^	55
Imidazole	6.95	10^4^	60

## Discussion

Proton transfer is an important process in biological systems and has profound impact on catalysis. In many hydrogen-evolving systems, proton transfer can be the rate-determining step, and thus identifying the proton donor offers the ability to tune catalysis and modulate mechanism.^37^,^38^ The robustness and activity of the NiRd model system offers an opportunity to elucidate, with high mechanistic precision, how the proton donor pK_a_ and concentration can impact HER activity. The most notable observation from these experiments is that the proton donor only weakly affects catalysis, which can be mapped to mechanistic information. If the initial, buffer-mediated proton transfer step were the rate-determining step, then modulating the proton donor through changing pK_a_ or concentration would be expected to have profound effects on activity, as previously observed in work by Bren and coworkers.^21^,^22^ For example, in voltammograms of catalysts where intermolecular proton transfer has been identified as a rate-limiting step, a linear dependence between current and concentration is observed. However, this was not observed for NiRd. Despite a 100-fold increase in buffer concentration, the observed normalized current was found to increase only approximately two-fold in all buffers (Fig. 3). This shallow dependence of about 0.3-fold/log[buffer] suggests that while this initial proton transfer is not the rate-determining step, it occurs on a similar timescale as the rate-determining step and therefore does impact catalysis, albeit weakly. Additionally, the normalized currents, turnover frequencies, and the onset potentials were found to be essentially independent of buffer identity between 10 mM and 1000 mM, though the proton donors used here span a range of 2 pK_a_ units, giving over 100-fold change in donor ability. The proton donor pK_a_ has been shown to be highly impactful for catalysis in systems where ECCE or ECEC mechanisms are at play for the HER, with the onset potential shifting − 59 mV/pKa unit per proton involved in the rate-determining step. A similar relationship would be expected if proton transfer were partially limiting but with a small Brönsted proportionality constant α. [39] In the NiRd system, no effect on onset potential is seen when the buffers are in moderate to high (≥ 10 mM) concentrations. An extremely shallow slope of -6 mV/pKa unit is observed under very low buffer concentrations. This further suggests that the initial proton donation step does not dominate the kinetic barrier for catalysis under typical operating conditions, consistent with the CECEC mechanism proposed previously, though mass transport limitations of protonated buffer to the electrode cannot be ruled out. Moreover, no trend in turnover frequency is observed as a function of pKa, with both the highest and lowest turnover frequencies observed for the most acidic buffers, or buffer size, as imidazole and pyridine have similar steric profiles but differ in their protonation rate by an order of magnitude. Further, MES and acetate show very similar onset potentials, despite dramatic differences in size.

From the electrochemical simulations, it is apparent that the catalytic onset potential reflects both the pK_a_ of the NiRd system and the electrochemical potential. Given the low pK_a_ of the nickel-thiolate active site, which is estimated at ~ 2.5 [16,18], the population of protonated NiRd is low in all of the buffers across the investigated pH values. Once protonated, however, electron transfer is favored at low potentials, and this initiates turnover. The elementary properties for catalysis can be extracted considering the qPFE analysis, which shows that 10-fold variation in the rate of buffer-donated proton transfer to the metal-bound thiolate is required for the more basic buffers. This increase in protonation rate is necessary to account for the minimal change in onset potential observed, which overcomes the lower thermodynamic propensity towards proton donation and could reflect increased intrinsic diffusion rates of the buffer molecule or increased dynamical motion of the active site in these buffers. The former option seems unlikely, given the similarity in size and charge between pyridine and imidazole, and therefore it seems that buffer can affect the motion of the active site. The simulations also suggest slight variation in the rate constant for intramolecular proton transfer and hydride formation, the rate-determining step, across buffers. While the values for this coupled process are lower than typically associated with protein loop motion, the overall rate constants of 15–60 s^− 1^ are higher than those previously reported for intramolecular proton transfer and hydride formation for synthetic complexes, which range from approximately 10^− 2^ – 10^− 3^s^− 1^[40].

Additional mechanistic insights were gained when performing catalysis at limiting proton donor concentrations. A surprising amount of catalysis was seen in unbuffered water, with NiRd retaining approximately 60% of the activity in unbuffered water relative to 10 mM acetate at pH 4.5. At low buffer concentrations, the pK_a_ of the proton donor impacts both activity and onset potential, implicating a shift in the rate-determining step from intramolecular thiol inversion to intermolecular proton transfer. This is likely due to the extremely low proton donor availability, which decreases the overall rate of bimolecular proton transfer to lower than the intramolecular process. The ability to access different mechanistic regimes with only minor perturbations to reaction conditions underscores the shallow potential energy surfaces observed in protein-based catalysts, which tends to be more analogous to native enzymes than synthetic compounds.

The mechanistic information obtained in these studies provides design guidelines for improved catalysts for HER that operate in aqueous solution. First, it is essential to maintain a proton donor at sufficiently high concentrations (> 10 mM) to drive high catalytic currents, indicating the importance of an effective buffering system. Moreover, the rate of protonation can be impacted by the nature of the buffer, independent of the pK_a_ or charge, as the increased protonation rates extracted from the simulations for MES and imidazole buffers over acetate and pyridine suggest increased active site flexibility or loop motion to expose the active site to buffer.^35^ The protein can thus be tuned such that the secondary sphere loops are better able to interact with buffer (e.g., through installation of side chains with complementary charges or strong H-bonding interactions). This could increase transient solvent accessibility of the active site, further mitigating any limiting effects from buffer protonation. Finally, as the rate-determining step for catalysis is shown to be an intramolecular process, modulating this proton transfer process offers another direction for catalyst optimization by increasing flexibility or designing a thiolate-containing loop with a lower transition state barrier. Resolving these targeted methods for improving activity that rely on the secondary and outer spheres and dynamical motion highlights the advantage a tunable protein scaffold can offer in developing highly active catalysts.

## Conclusions

Artificial metalloenzymes offer insight into complex native systems in simpler biochemical frameworks and provide opportunities for rational catalyst design and optimization. In this study, the proton transfer processes in a model hydrogenase enzyme, NiRd, which is proposed to evolve hydrogen under a similar mechanism to the native system, was interrogated through protein film electrochemistry. The catalytic current and onset potential were found to be only weakly dependent on buffer concentration, suggesting that intermolecular proton transfer is not the rate-determining step for catalysis. Under dilute buffering conditions, however, the rate-determining step changes to depend on intermolecular proton transfer. These findings add to the growing awareness of the role that buffers often play in catalysis, where their presence is generally not innocent. The implications of this study may also be considered within the complex environment of a cell, where the presence of diverse proton donors may provide intrinsic biological control over activity.

## Supplementary Information

Below is the link to the electronic supplementary material.

Supplementary Material 1

## Data Availability

All data supporting the findings of this study are available within the paper and its Supporting Information.
