# Supplementary material for: Identifying the proton donor in electrocatalytic hydrogen evolution by a model hydrogenase
Source: J Biol Inorg Chem. 2026 Aug 4;31(5):395–408. doi: 10.1007/s00775-026-02166-9 (PMC13562281; doi:10.1007/s00775-026-02166-9)
Supplement: Supplementary file 1 [file 775_2026_2166_MOESM1_ESM.pdf]

Identifying the proton donor in electrocatalytic hydrogen evolution by a model hydrogenase

Riley E. Stein[1], Ashlee E. Wertz[1], Peter J. Moore[1], Hannah S. Shafaat[1,2]*

[1]*Department of Chemistry and Biochemistry, The Ohio State University, Columbus, OH, 43210, USA*

[2]*Department of Chemistry and Biochemistry, University of California, Los Angeles, Los Angeles, CA, 90095, USA*

Supporting Information

Supplemental Materials and Methods…………………………………………..**S4 – S5**

Supplemental Figures……………………………………………………….....**S6 – S43**

Figure S1. CVs of NiRd in Acetate pH 4.5 Trial 1……………………………………**S6**

Figure S2. CVs of NiRd in Acetate pH 4.5 Trial 2……………………………………**S7**

Figure S3. CVs of NiRd in Acetate pH 4.5 Trial 3……………………………………**S8**

Figure S4. CVs of NiRd in Acetate pH 5.5 Trial 1……………………………………**S9**

Figure S5. CVs of NiRd in Acetate pH 5.5 Trial 2…………..…………………………**S10**

Figure S6. CVs of NiRd in Acetate pH 5.5 Trial 3………..……………………………**S11**

Figure S7. CVs of NiRd in Imidazole pH 4.5 Trial 1………..…………………………**S12**

Figure S8. CVs of NiRd in Imidazole pH 4.5 Trial 2………..…………………………**S13**

Figure S9. CVs of NiRd in Imidazole pH 4.5 Trial 3………..…………...………………**S14**

Figure S10. CVs of NiRd in Imidazole pH 5.5 Trial 1………..…………………………..**S15**

Figure S11. CVs of NiRd in Imidazole pH 5.5 Trial 2………..…………………………..**S16**

Figure S12. CVs of NiRd in Imidazole pH 5.5 Trial 3………..…………………………..**S17**

Figure S13. CVs of NiRd in MES pH 4.5 Trial 1………..………………………………..**S18**

Figure S14. CVs of NiRd in MES pH 4.5 Trial 2………..………………………………..**S19**

Figure S15. CVs of NiRd in MES pH 4.5 Trial 3………..………………………………..**S20**

Figure S16. CVs of NiRd in MES pH 5.5 Trial 1………..………………………………..**S21**

Figure S17. CVs of NiRd in MES pH 5.5 Trial 2………..………………………………..**S22**

Figure S18. CVs of NiRd in MES pH 5.5 Trial 3………..……………………...……………...**S23**

Figure S19. CVs of NiRd in Pyridine pH 4.5 Trial 1………..……………………………**S24**

Figure S20. Pyridine Catalytic Wave Derivatives………..……………………………....**S25**

Figure S21. CVs of NiRd in Pyridine pH 4.5 Trial 2………..……………………………**S26**

Figure S22. CVs of NiRd in Pyridine pH 4.5 Trial 3………..……………………………**S27**

Figure S23. CVs of NiRd in Pyridine pH 5.5 Trial 1………..……………………………**S28**

Figure S24. CVs of NiRd in Pyridine pH 5.5 Trial 2………..……………………………**S29**

Figure S25. CVs of NiRd in Pyridine pH 5.5 Trial 3………..……………………………**S30**

Figure S26. Procedure used to identify the onset potential of NiRd HER..................**S31**

Figure S27. UV-Vis Spectrum of NiRd with added Imidazole………..…………………**S32**

Figure S28. Normalized Current and Onset Potential at pH 5.5………..……………...**S33**

Figure S29. Onset Potential as a Function of Protonated Buffer………..……………..**S34**
Figure S30. NiRd current over time………..………………………………………...**S35**
Figure S31. Correction Factor Application………..………………………………**S36**
Figure S32. CVs of NiRd in water at pH 4.5………..……………………………**S37**
Figure S33. CVs of NiRd in water at pH 3.5………..……………………………**S38**
Figure S34. CVs of NiRd in water at pH 5.5………..……………………………**S39**
Figure S35. Simulated mechanistic parameters of NiRd in acetate pH 4.5…………..**S40**
Figure S36. Simulated mechanistic parameters of NiRd in pyridine pH 4.5…..……**S41**
Figure S37. Simulated mechanistic parameters of NiRd in MES pH 4.5……………...**S42**
Figure S38. Simulated mechanistic parameters of NiRd in imidazole pH 4.5………..**S43**

Table S1. Tabulated onset potentials with error for each buffer..................................**S44**

Appendix I…………..………..……………………………………………….…**S43 – S50**
Figure S39. Simulated mechanistic parameters for best-fit k_a values.........................**S51**
Figure S40. Simulated mechanistic parameters for alternative k_a values...................**S52**
Figure S41. Sensitivity of mechanistic parameters..**S53**

Supplementary References…………………………………………………..…..**S54**

Supplemental Materials and Methods

General Materials.

All materials were prepared as described in the main text. Solutions were prepared by dissolving the appropriate buffer in MilliQ water (18.2 MΩ). All solutions were prepared with 100 mM KCl as a supporting electrolyte.

General Experimental.

All experiments were performed in an anaerobic glovebox with degassed solutions. Following covalently coupling of protein to pyrolytic graphite edge (PGE) electrodes, adventitious protein was rinsed from the electrode and electrodes were transferred into the glovebox. Protein films were then placed into a three neck round bottom flask with the PGE as the working, a Ag/AgCl reference wire, and a platinum counter wire. Cyclic voltammograms (CVs) were conducted in order at 100, 25, 50, 200, 500, and 1000 mV/s with two full potential scans. The second scan was used for analysis. Buffer concentrations of 10, 30, 100, 300, and 1000 mM were examined. For imidazole and pyridine, 1000 mM concentrations were omitted from analysis as electrochemical events unrelated to NiRd HER heavily overlapped with NiRd HER signal and did not allow for analysis. Before and after every surveyed buffer concentration, a reference scan in 100 mM of the same buffer was taken to account for film deactivation. For low concentration buffer experiments, 100 mM acetate at the same pH was used as the reference. For experiments conducted in water, water was carefully adjusted to the desired pH, and 100 mM acetate at the same pH was also used as the reference solution. UV−visible spectra were measured using a Shimadzu UV-2600 spectrophotometer. Protein samples were diluted to ∼131 μM from a stock of ∼3−5 mM Rd in 50 mM Tris, pH 8.0 buffer with 100 mM acetate buffer pH 4.5.

Electrochemical Analysis.

Data analysis was performed in Igor Pro (Wavemetrics) and QSoas.[1] All experiments were repeated with different protein batches, different buffer preparations, and different films for reproducibility and the individual trials are shown in Figures S2 – S26.

Normalized current and onset potential

Catalytic currents were obtained from a point 100 mV more cathodic of the onset potential. Catalytic current values were then normalized to the current value from the reference solutions to account for film deactivation during the electrochemical experiments. Due to the irreversibility and lack of a plateau during catalysis, the onset potential was determined by the inflection point of catalytic current, which has been noted to be a more reliable indicator of the electrochemical potentials of irreversible systems.[2] The inflection point was identified by taking the first derivative of the CV and fitting the catalytic feature to a sigmoid where the midpoint of the fit was the onset potential. This is equivalent to the minimum of the second derivative of the voltammogram (**Figure S1**). In the case of imidazole, electrochemical events more anodic of NiRd HER complicated identification of the point 100 mV more anodic of the onset potential. To account for this, a line was extrapolated from the cathodic sweep of the CV in the region with only non-faradaic current.

Turnover Frequencies

Turnover frequencies (TOFs) were calculated by the equation presented in the main text (Eq S1)

$$TOF = \frac{i_{E_{analysis}}}{\Gamma_{NiRd} nF}$$

where $i_{E_{analysis}}$ is the current analyzed at a given potential obtained as previously described, Γ_{NiRd} is the electroactive coverage of NiRd, n is the number of electrons transferred in the electrochemical conversion, 2 for proton reduction, and F is Faraday's constant. Γ_{NiRd} was approximated by determining the electroactive coverage from the noncatalytic $Fe^{II/III}$ redox couple from the FeRd and assuming a 1:1 ratio of NiRd to FeRd. This has previously been shown to be a valid assumption.[3,4] However, in this study, the same protein film was repeatedly subjected to catalytic conditions for extended periods of time, resulting in deactivation of NiRd, making the 1:1 assumption invalid. To account for this, a procedure for film deactivation was adapted from a study from Fourmond and coworkers.[5] This procedure enables the quantitation of film deactivation to account for ratios of NiRd to FeRd being less than 1:1.

To account for NiRd deactivation during subjection to electrocatalytic conditions, the catalytic current of the same film was analyzed for every 100 mM reference solution over the course of experimental conditions (i.e. the entire time it took to scan the different buffer concentrations). The catalytic current of the reference solution was plotted against time and fit to an exponential function (Eq S2):

$$i_{reference}(t) = i_0 + A \cdot \exp\left(\frac{-t}{\tau}\right)$$

where i_0 is the initial current from the reference, A is a scaling factor, t is the time the reference solution was scanned, and τ is the rate of decay. To approximate the inactivation of the film at the time of the experimental condition, the time of the experimental condition (t_{exp}) was used to solve for the reference current at that time to obtain i_{exp}. i_{exp} was then divided by i_0 to determine the correction factor for film deactivation and the turnover frequency obtained for the condition at t_{exp} was divided by the correction factor. This resulted in trends in TOF that track exactly with normalized current, which by virtue of utilizing a reference solution before and after the experimental condition already had this consideration accounted for. With this approach, the initial scan of the reference solution was assumed to have a 1:1 ratio of NiRd to FeRd, which has previously shown to be true.

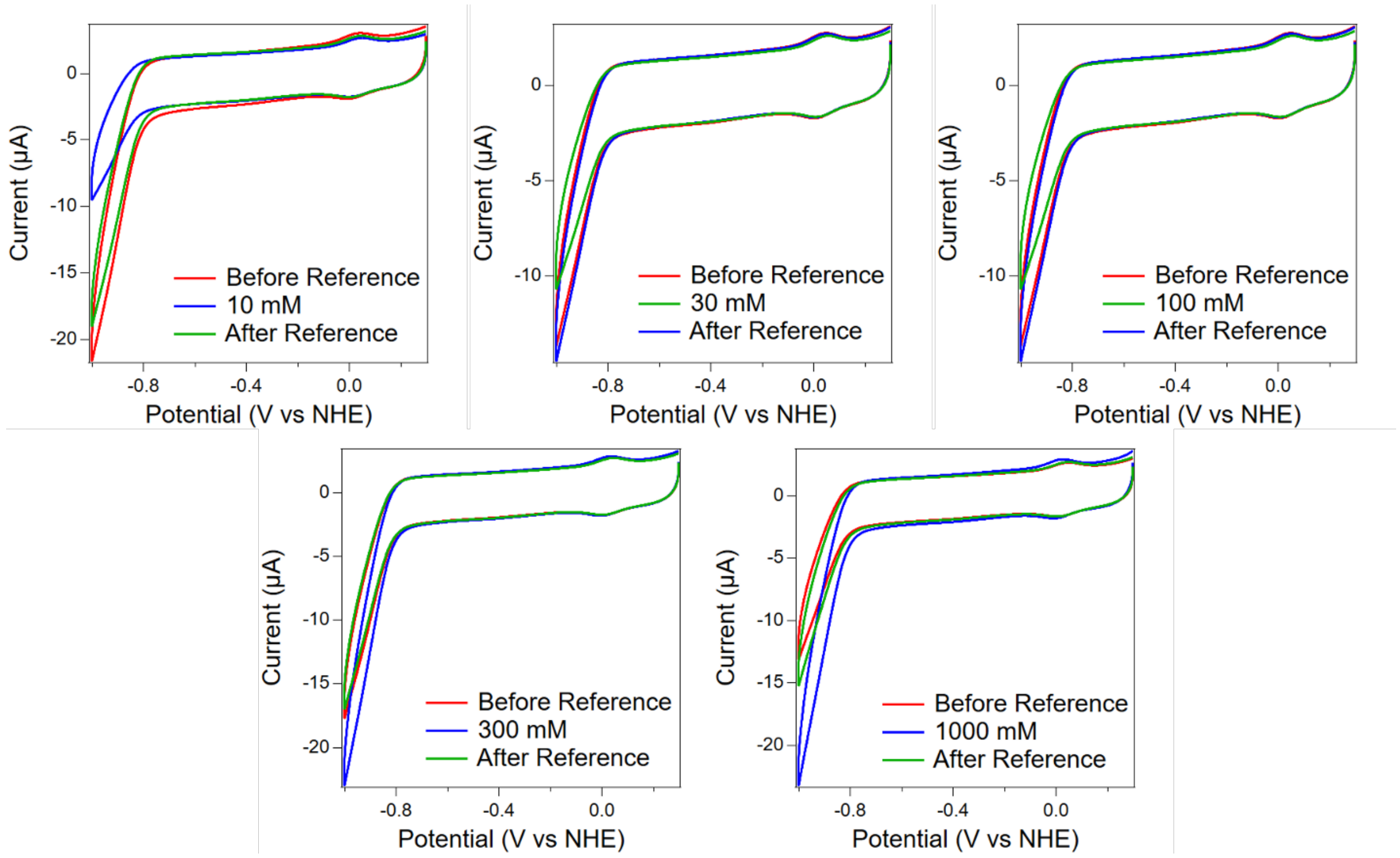

Figure S1. Acetate pH 4.5: Trial 1. Cyclic voltammograms of NiRd covalently attached to electrode surface with a unique film for each trial. For each buffer concentration surveyed, a reference in a 100 mM buffer solution was recorded before (*red trace*) and after (*green trace*) the experiment was conducted in the buffer concentration of interest (*blue trace*).

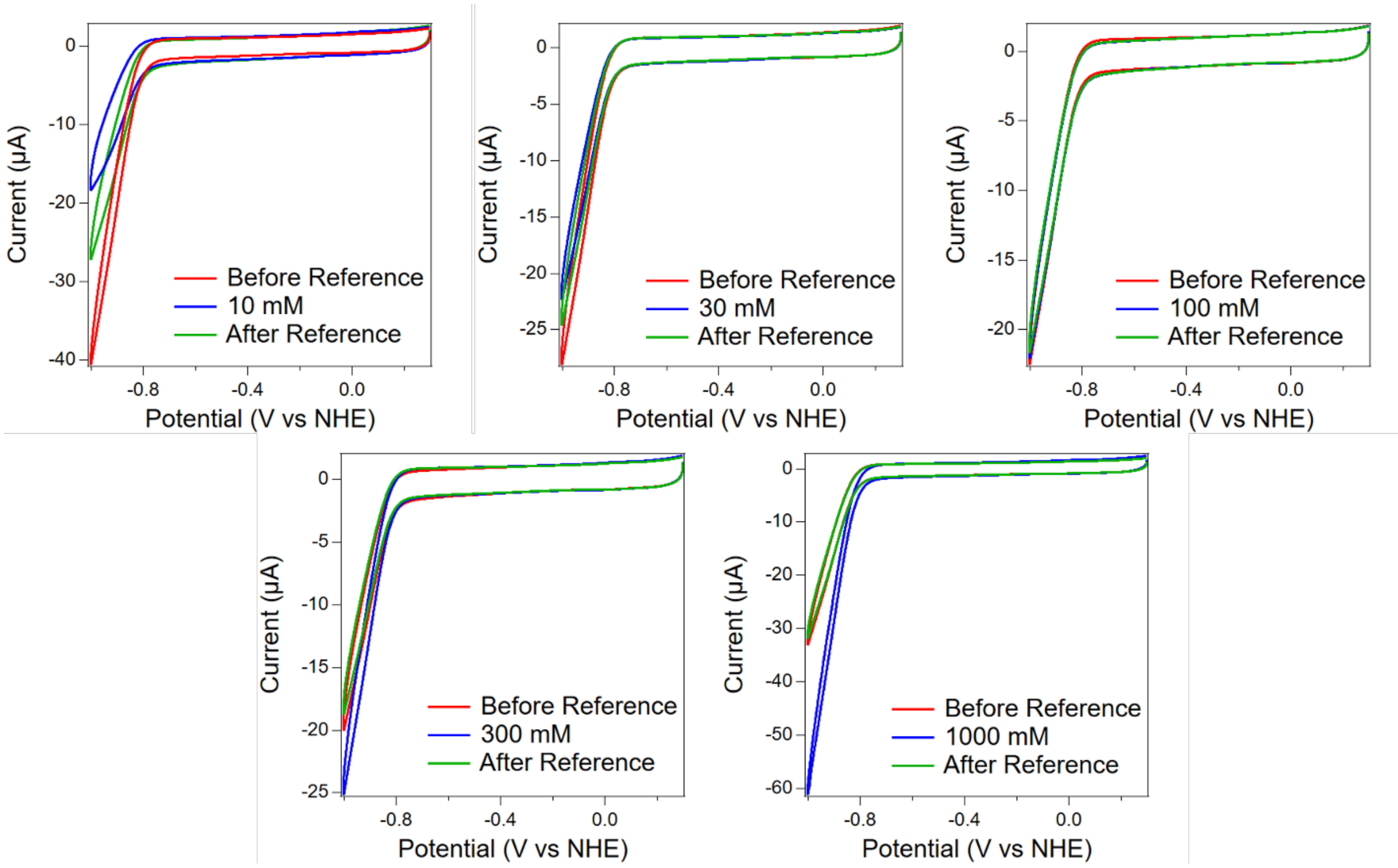

Figure S2. Acetate pH 4.5 Trial 2. Cyclic voltammograms of NiRd covalently attached to electrode surface with a unique film from other trials. For each buffer concentration surveyed, a reference in a 100 mM buffer solution was recorded before (red trace) and after (green trace) the experiment was conducted in the buffer concentration of interest (blue trace).

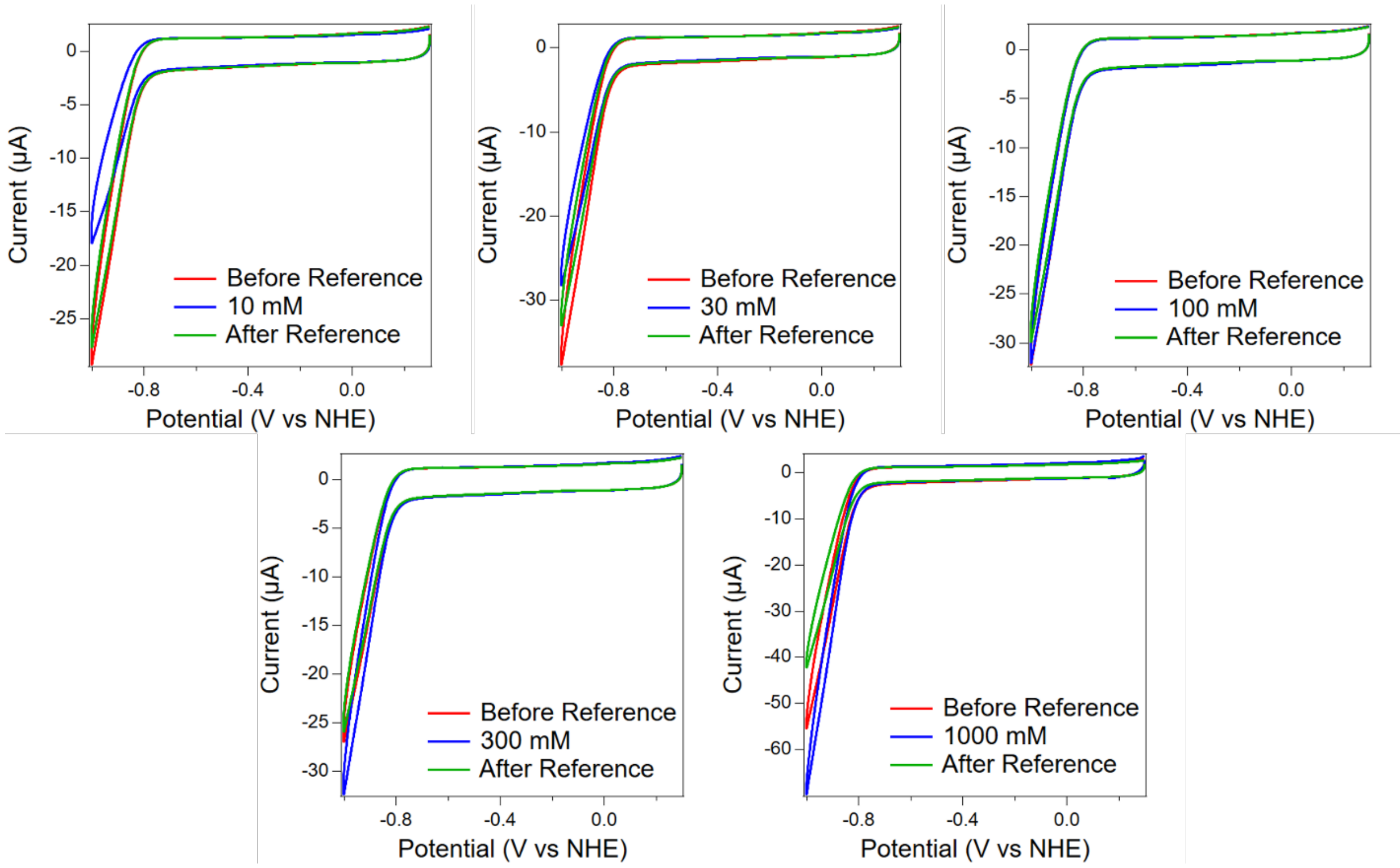

Figure S3. Acetate pH 4.5 Trial 3. Cyclic voltammograms of NiRd covalently attached to electrode surface with a unique film from other trials. For each buffer concentration surveyed, a reference in a 100 mM buffer solution was recorded before (red trace) and after (green trace) the experiment was conducted in the buffer concentration of interest (blue trace).

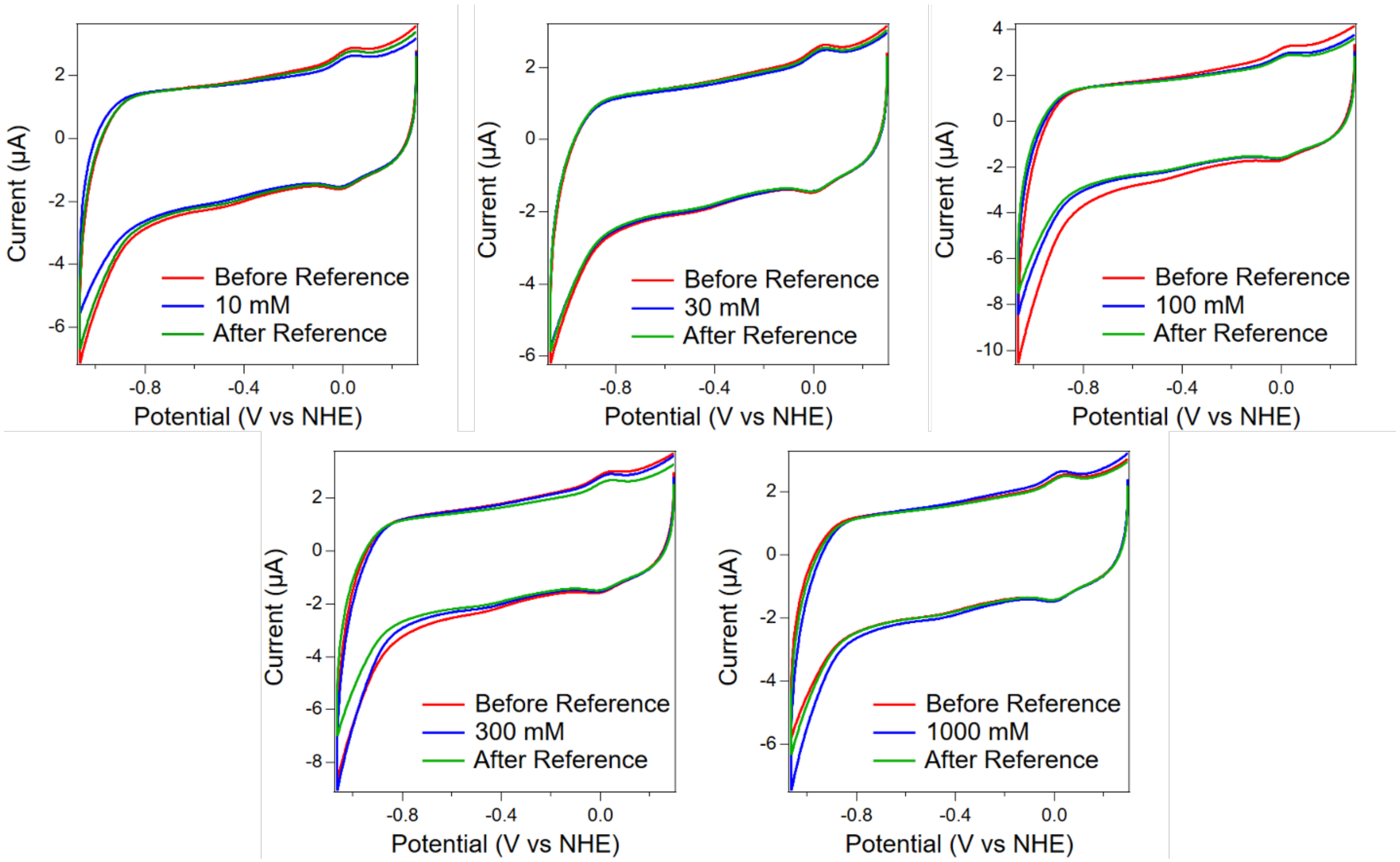

Figure S4. Acetate pH 5.5 Trial 1. Cyclic voltammograms of NiRd covalently attached to electrode surface with a unique film from other trials. For each buffer concentration surveyed, a reference in a 100 mM buffer solution was recorded before (red trace) and after (green trace) the experiment was conducted in the buffer concentration of interest (blue trace).

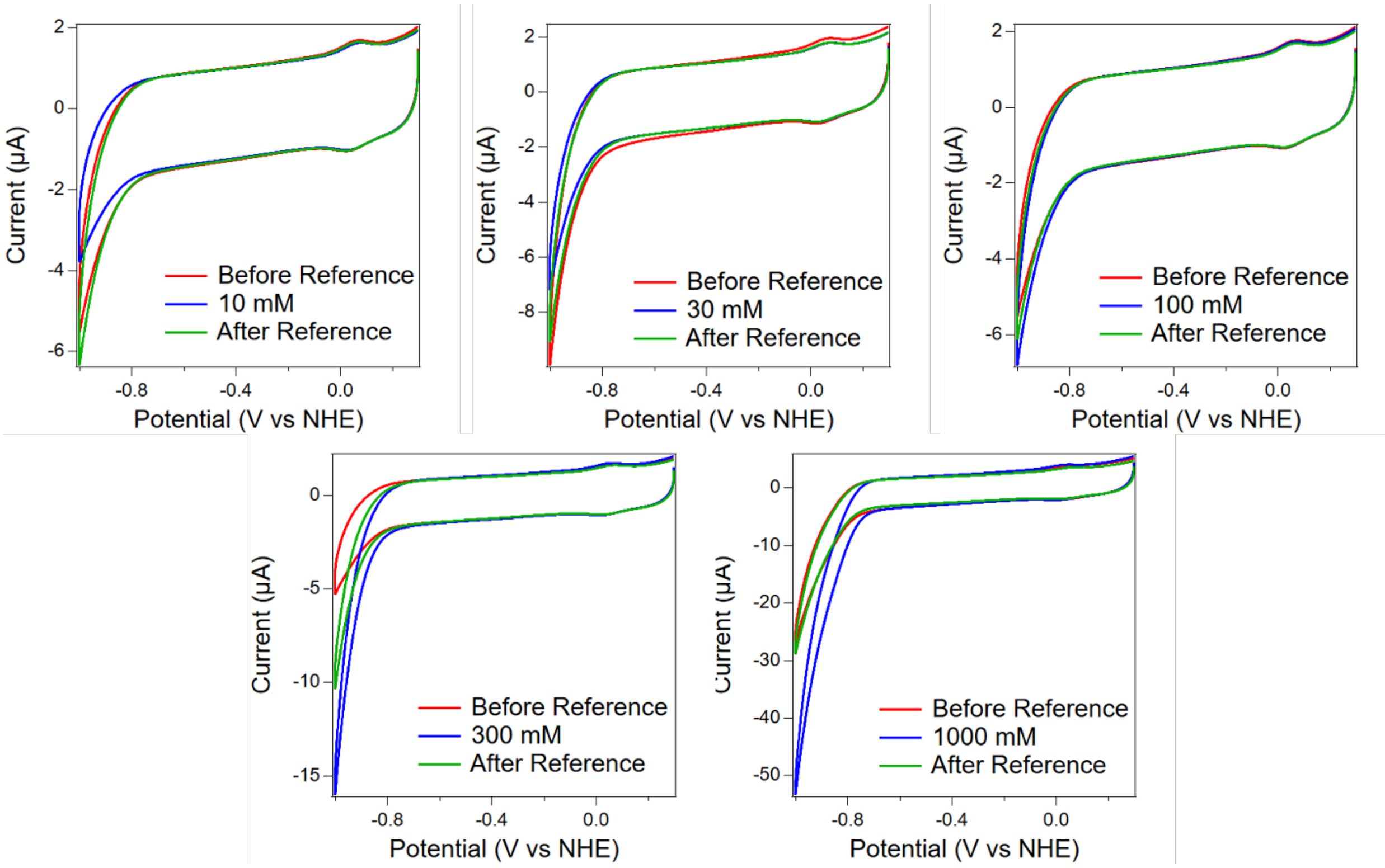

Figure S5. Acetate pH 5.5 Trial 2. Cyclic voltammograms of NiRd covalently attached to electrode surface with a unique film from other trials. For each buffer concentration surveyed, a reference in a 100 mM buffer solution was recorded before (red trace) and after (green trace) the experiment was conducted in the buffer concentration of interest (blue trace).

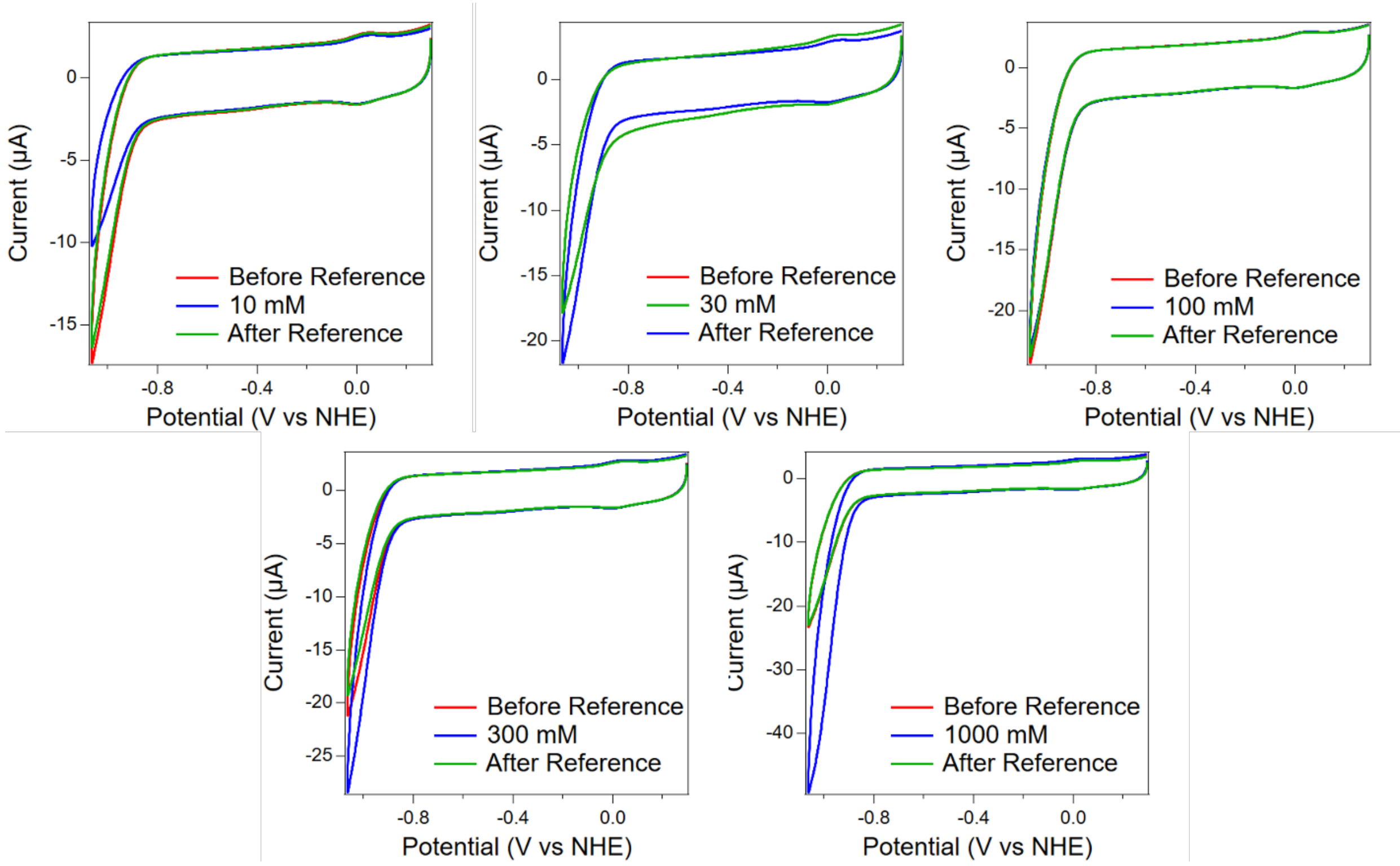

Figure S6. Acetate pH 5.5 Trial 3. Cyclic voltammograms of NiRd covalently attached to electrode surface with a unique film from other trials. For each buffer concentration surveyed, a reference in a 100 mM buffer solution was recorded before (red trace) and after (green trace) the experiment was conducted in the buffer concentration of interest (blue trace).

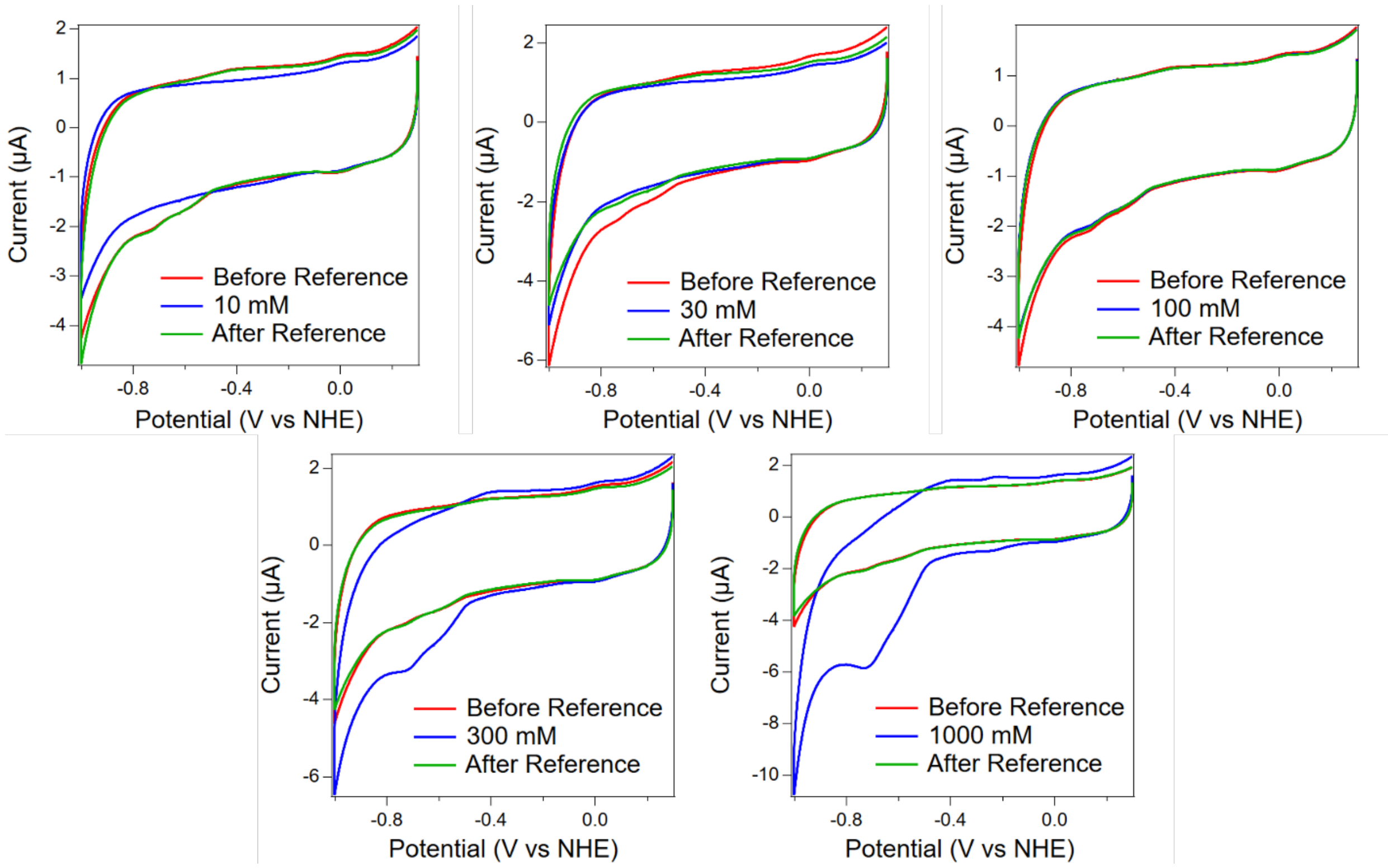

Figure S7. Imidazole pH 4.5 Trial 1. Cyclic voltammograms of NiRd covalently attached to electrode surface with a unique film from other trials. For each buffer concentration surveyed, a reference in a 100 mM buffer solution was recorded before (red trace) and after (green trace) the experiment was conducted in the buffer concentration of interest (blue trace). Electrochemical events related to the buffer are present around -300 and -700 mV, which are especially pronounced at 1000 mM of imidazole.

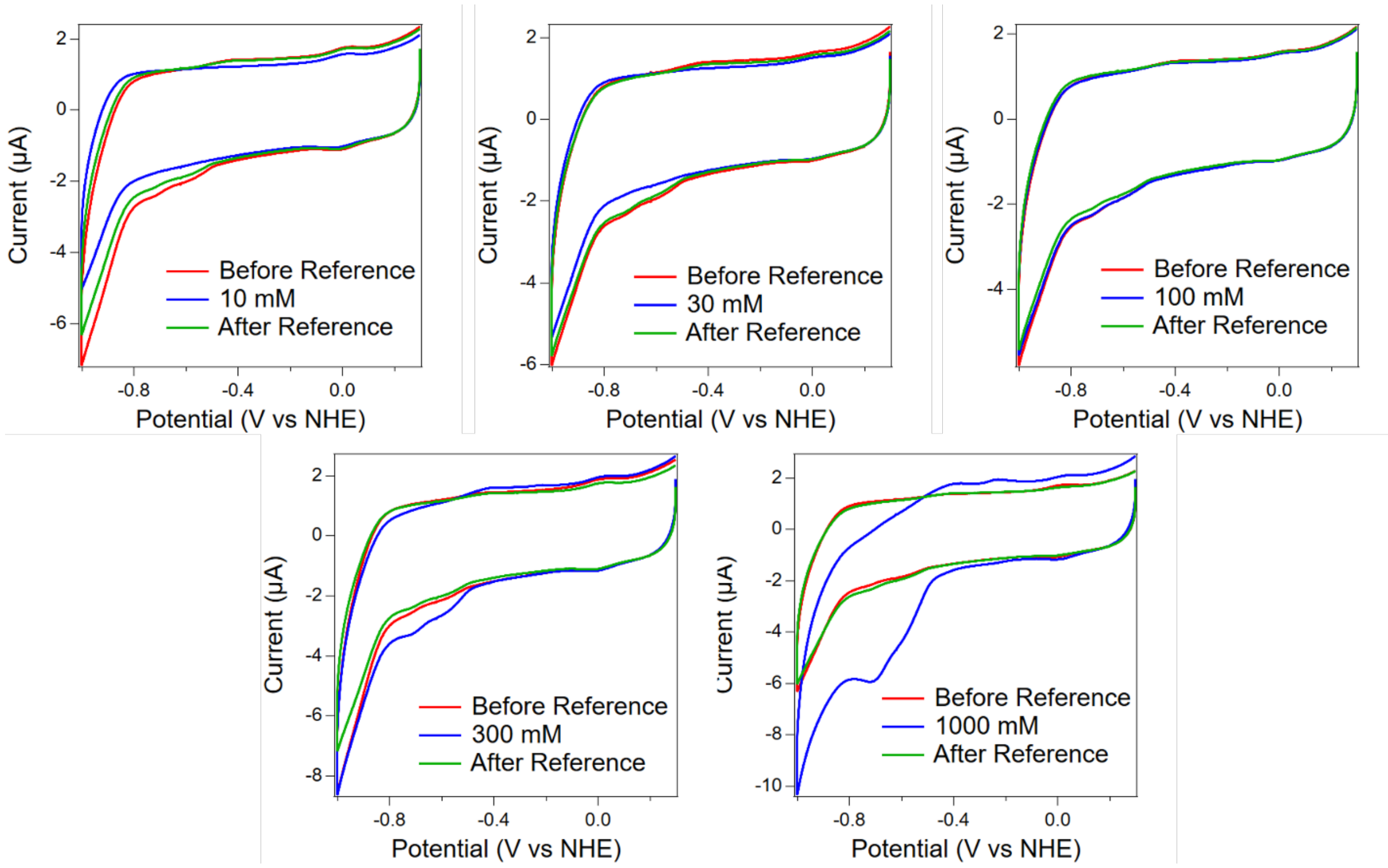

Figure S8. Imidazole pH 4.5 Trial 2. Cyclic voltammograms of NiRd covalently attached to electrode surface with a unique film from other trials. For each buffer concentration surveyed, a reference in a 100 mM buffer solution was recorded before (red trace) and after (green trace) the experiment was conducted in the buffer concentration of interest (blue trace).Electrochemical events related to the buffer are present around -300 and -700 mV, which are especially pronounced at 1000 mM of imidazole.

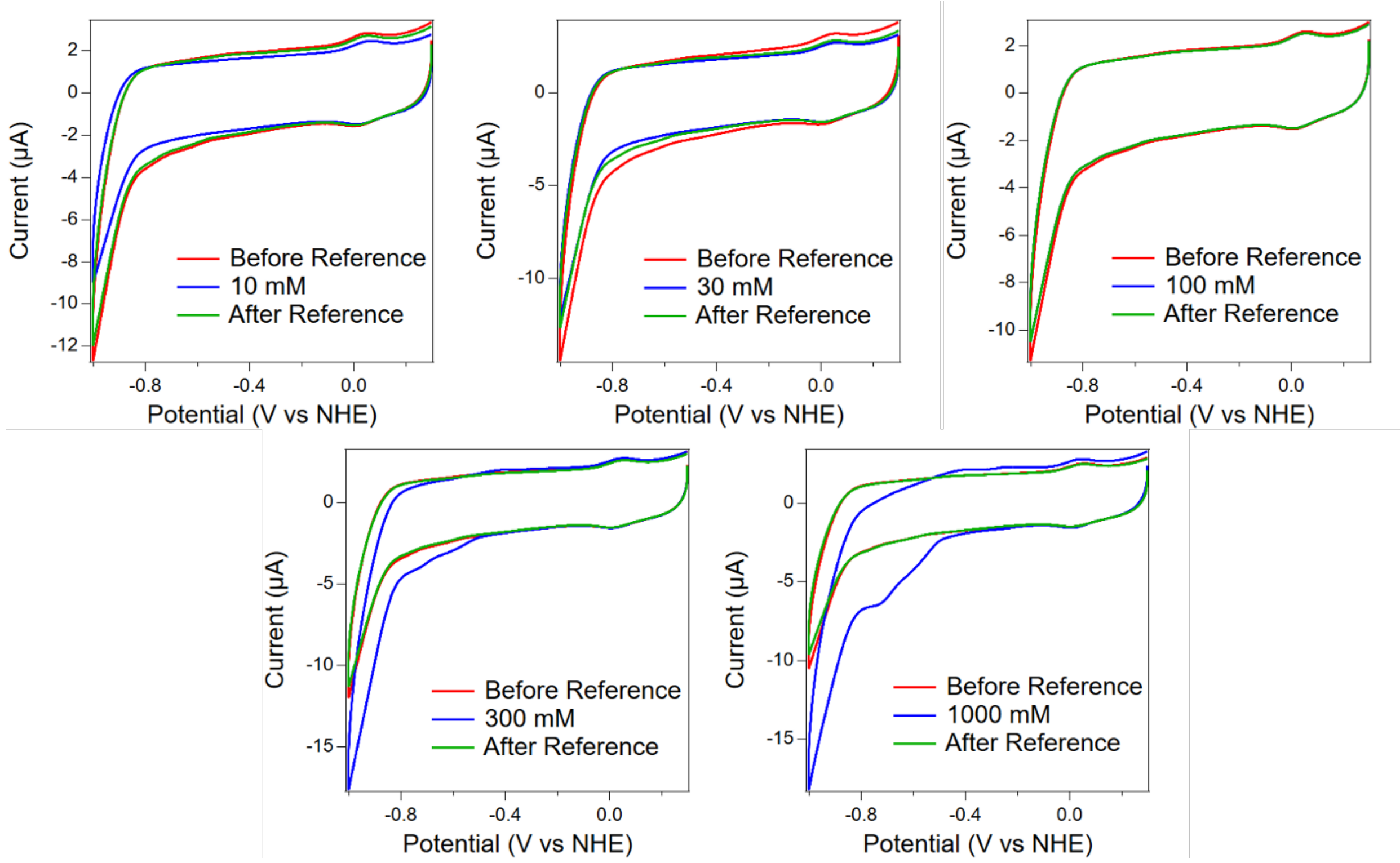

Figure S9. Imidazole pH 4.5 Trial 3. Cyclic voltammograms of NiRd covalently attached to electrode surface with a unique film from other trials. For each buffer concentration surveyed, a reference in a 100 mM buffer solution was recorded before (red trace) and after (green trace) the experiment was conducted in the buffer concentration of interest (blue trace).Electrochemical events related to the buffer are present around -300 and -700 mV, which are especially pronounced at 1000 mM of imidazole.

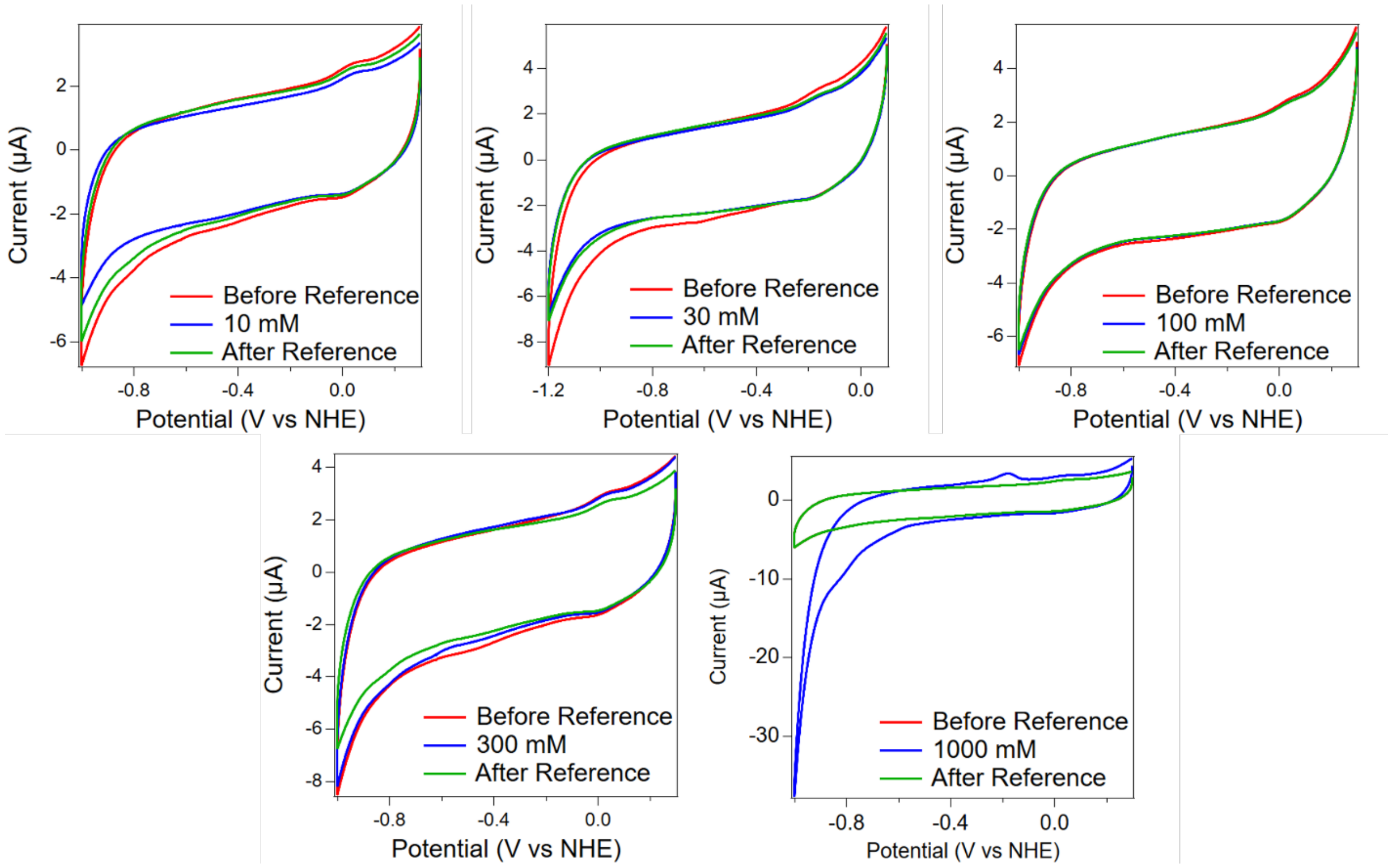

Figure S10. Imidazole pH 5.5 Trial 1. Cyclic voltammograms of NiRd covalently attached to electrode surface with a unique film from other trials. For each buffer concentration surveyed, a reference in a 100 mM buffer solution was recorded before (red trace) and after (green trace) the experiment was conducted in the buffer concentration of interest (blue trace).Electrochemical events related to the buffer are present around -300 and -700 mV, which are especially pronounced at 1000 mM of imidazole and precluded analysis of NiRd HER. As a result, 1000 mM imidazole was not recorded further.

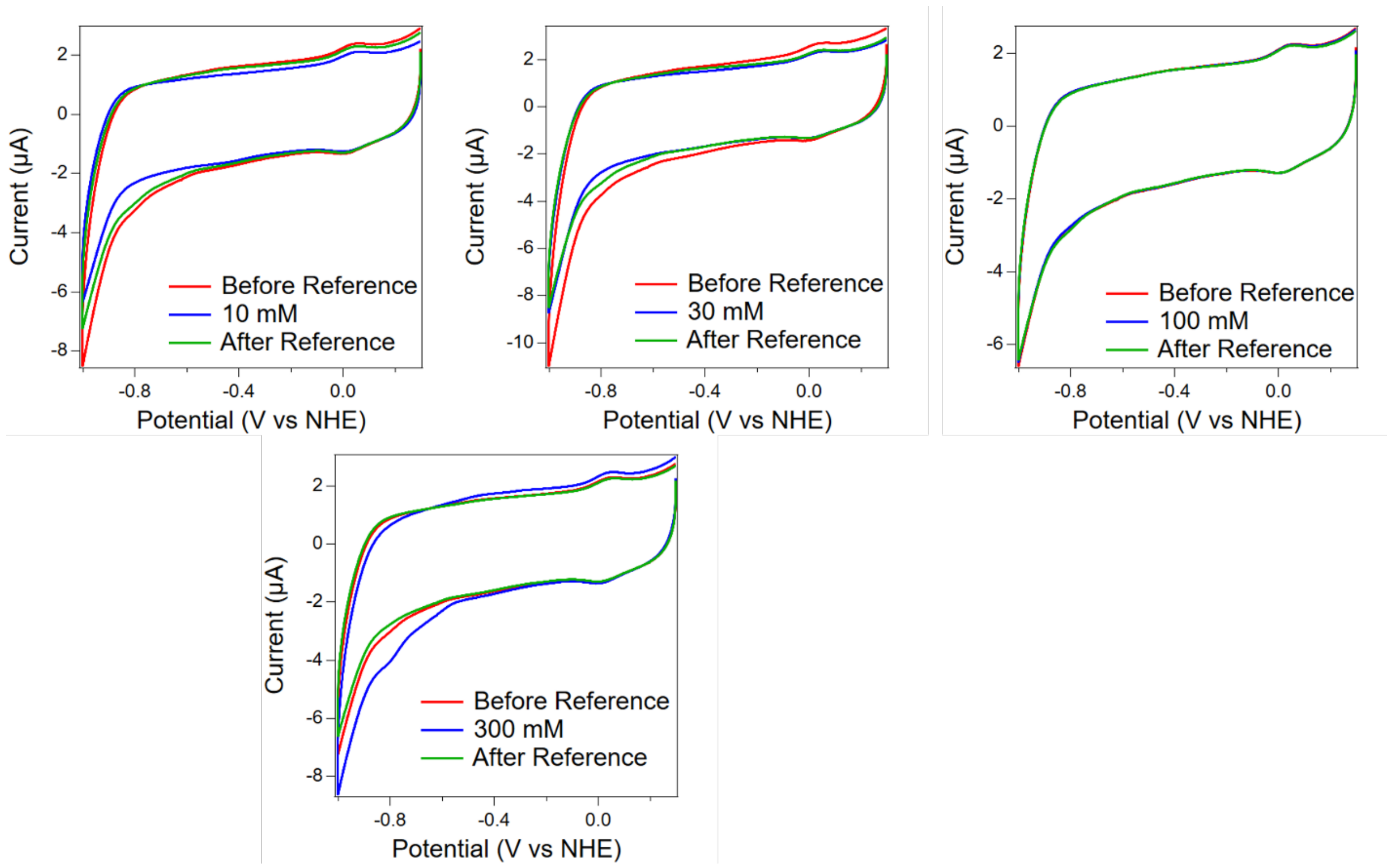

Figure S11. Imidazole pH 5.5 Trial 2. Cyclic voltammograms of NiRd covalently attached to electrode surface with a unique film from other trials. For each buffer concentration surveyed, a reference in a 100 mM buffer solution was recorded before (red trace) and after (green trace) the experiment was conducted in the buffer concentration of interest (blue trace).Electrochemical events related to the buffer are present around -300 and -700 mV.

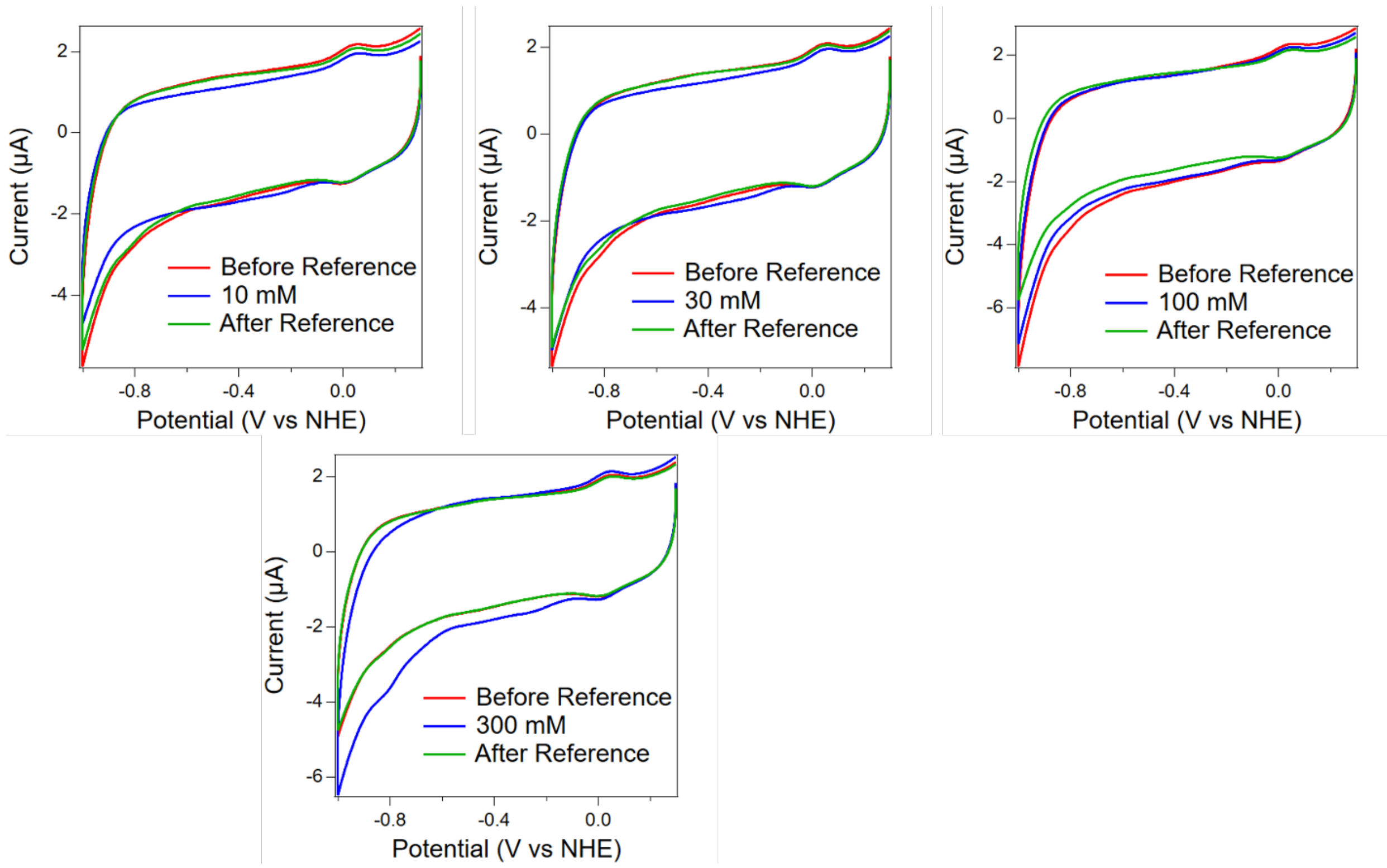

Figure S12. Imidazole pH 5.5 Trial 3. Cyclic voltammograms of NiRd covalently attached to electrode surface with a unique film from other trials. For each buffer concentration surveyed, a reference in a 100 mM buffer solution was recorded before (red trace) and after (green trace) the experiment was conducted in the buffer concentration of interest (blue trace).Electrochemical events related to the buffer are present around -300 and -700 mV.

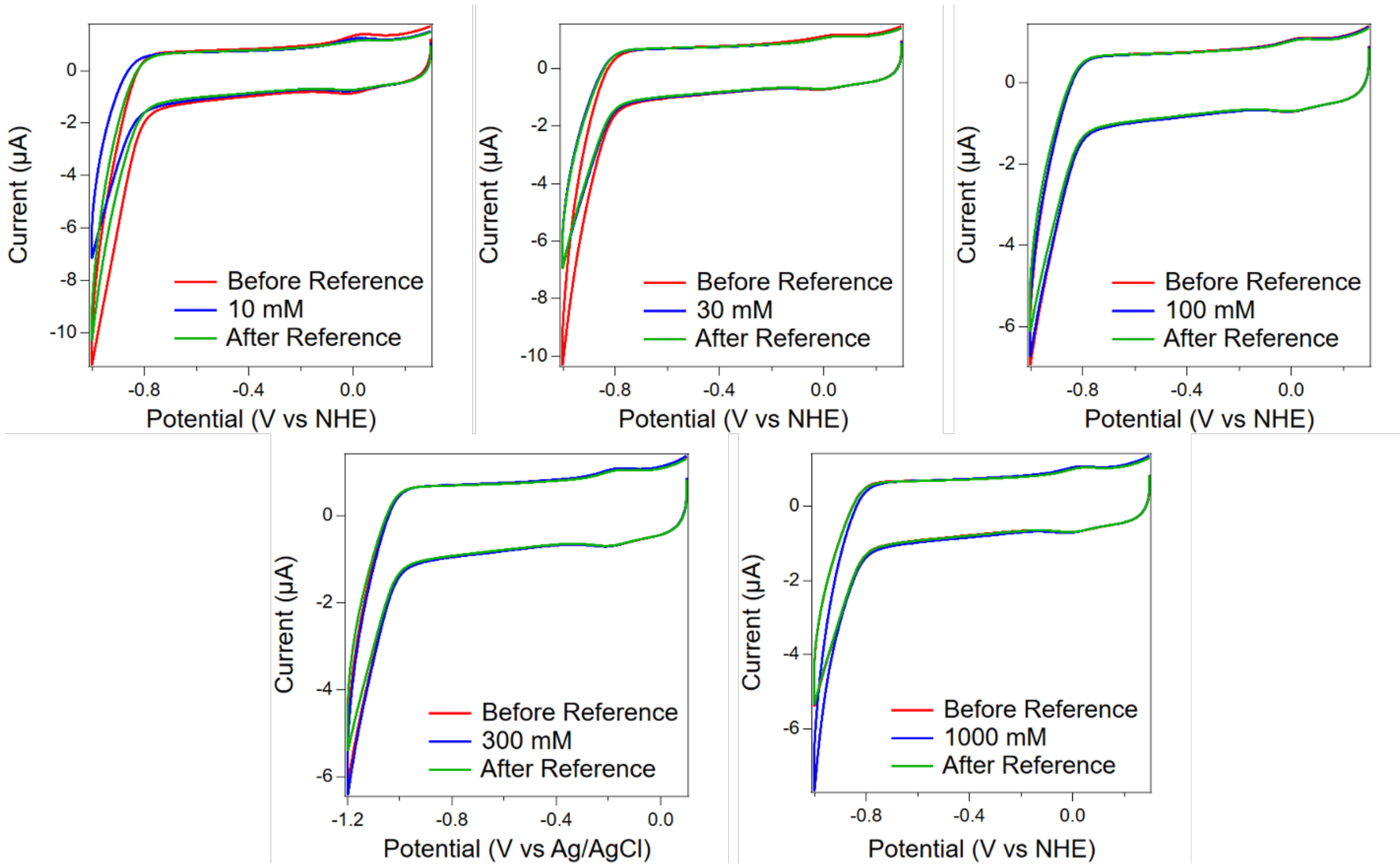

Figure S13. MES pH 4.5 Trial 1. Cyclic voltammograms of NiRd covalently attached to electrode surface with a unique film from other trials. For each buffer concentration surveyed, a reference in a 100 mM buffer solution was recorded before (red trace) and after (green trace) the experiment was conducted in the buffer concentration of interest (blue trace).

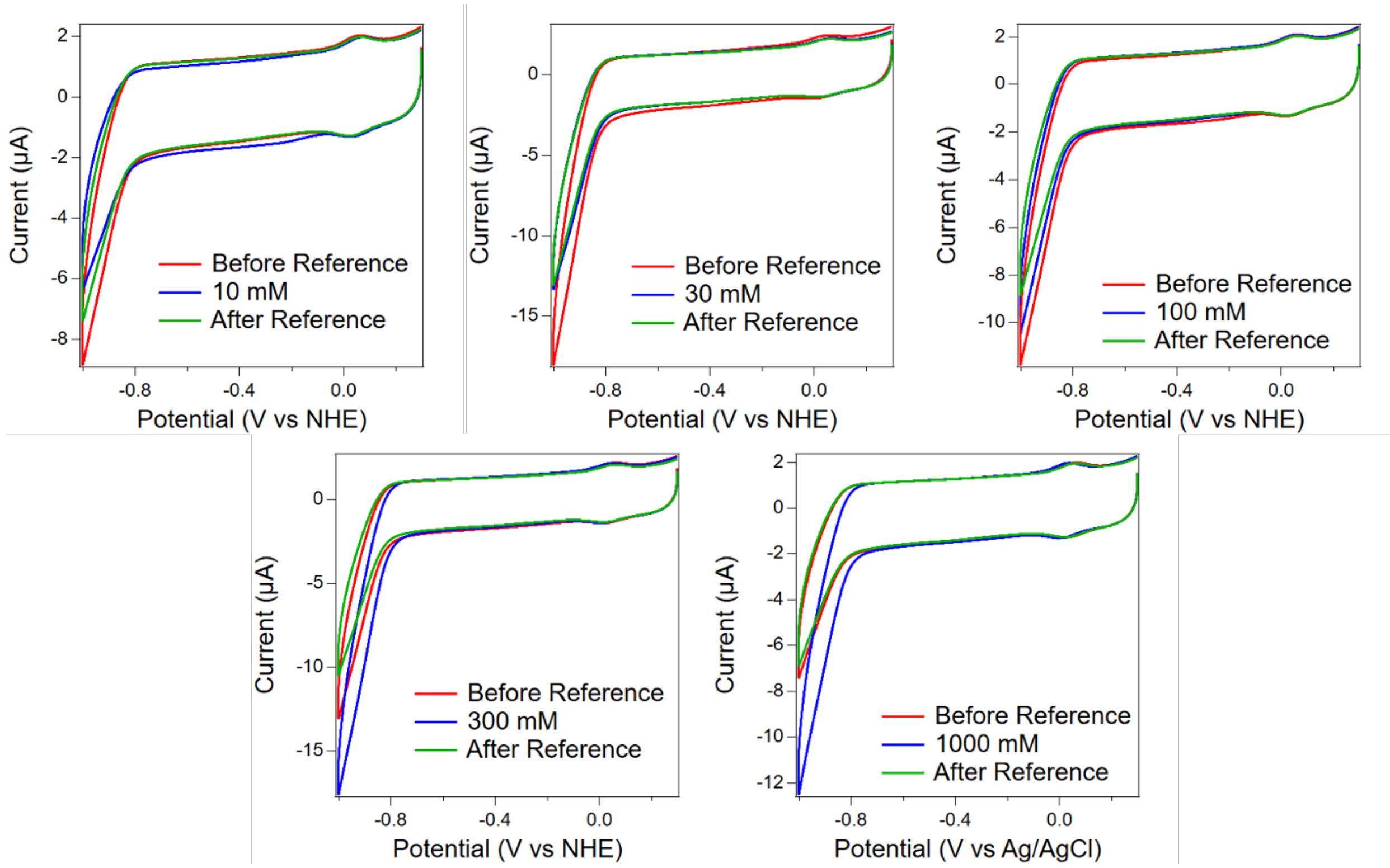

Figure S14. MES pH 4.5 Trial 2. Cyclic voltammograms of NiRd covalently attached to electrode surface with a unique film from other trials. For each buffer concentration surveyed, a reference in a 100 mM buffer solution was recorded before (red trace) and after (green trace) the experiment was conducted in the buffer concentration of interest (blue trace).

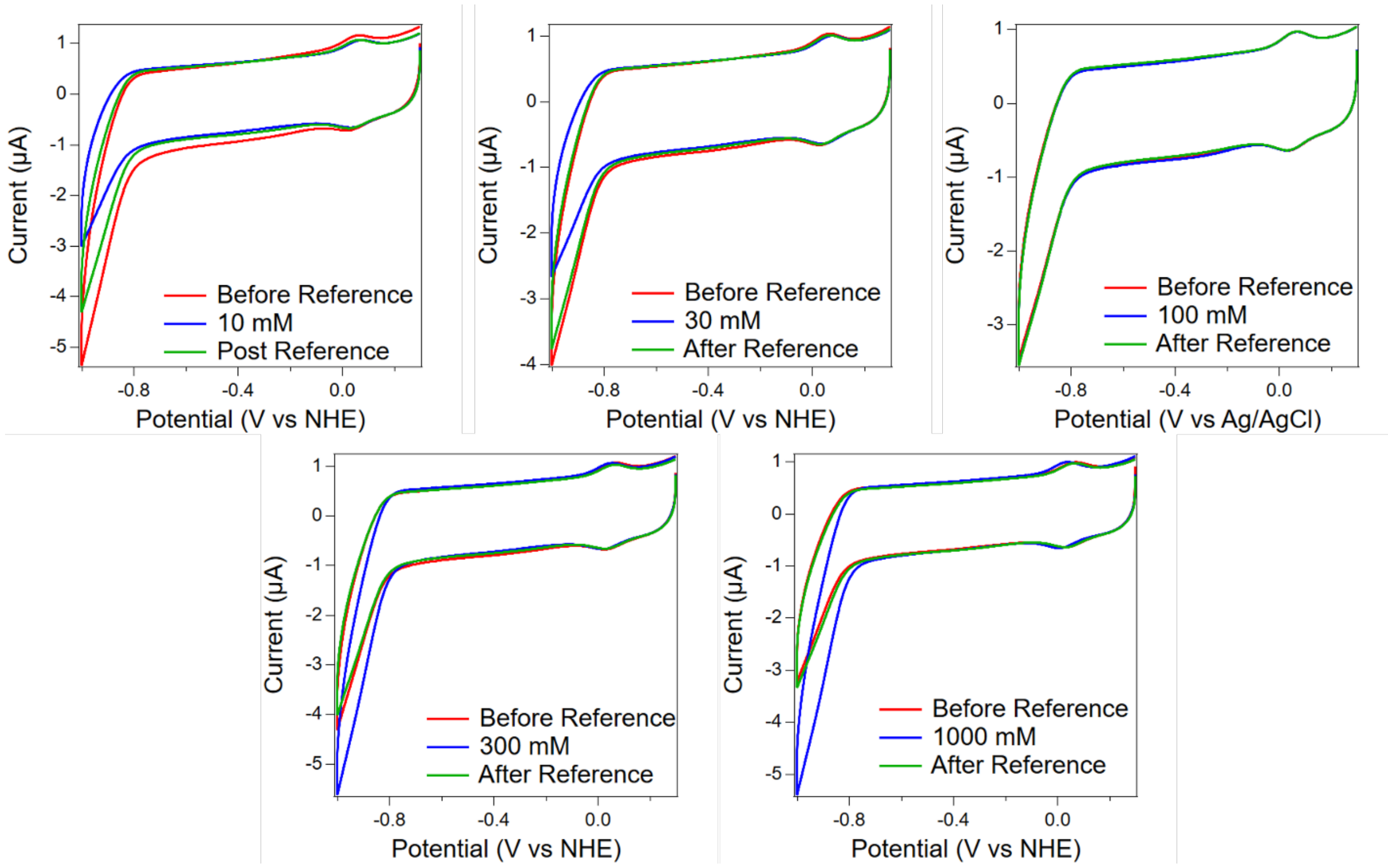

Figure S15. MES pH 4.5 Trial 3. Cyclic voltammograms of NiRd covalently attached to electrode surface with a unique film from other trials. For each buffer concentration surveyed, a reference in a 100 mM buffer solution was recorded before (red trace) and after (green trace) the experiment was conducted in the buffer concentration of interest (blue trace).

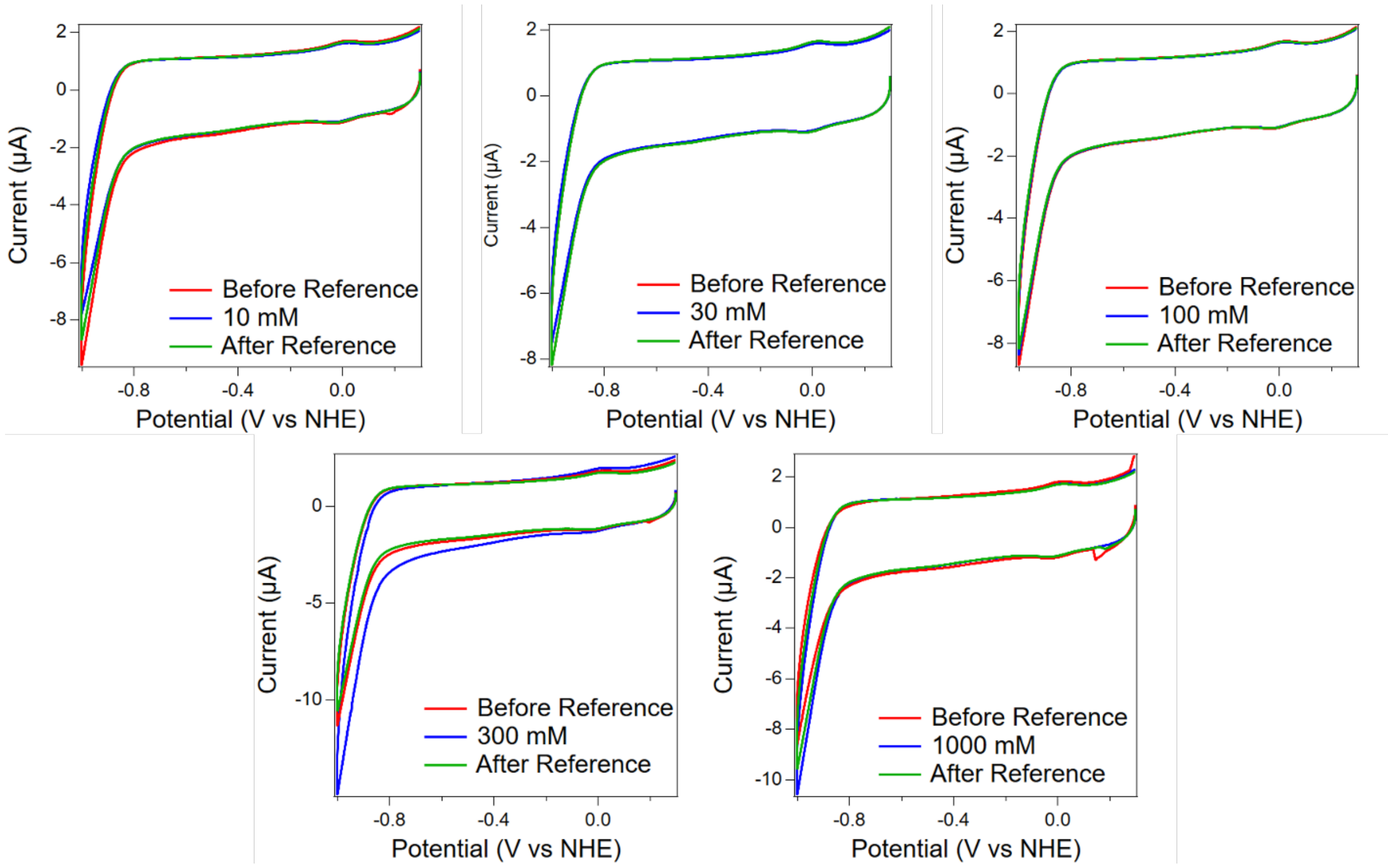

Figure S16. MES pH 5.5 Trial 1. Cyclic voltammograms of NiRd covalently attached to electrode surface with a unique film from other trials. For each buffer concentration surveyed, a reference in a 100 mM buffer solution was recorded before (red trace) and after (green trace) the experiment was conducted in the buffer concentration of interest (blue trace).

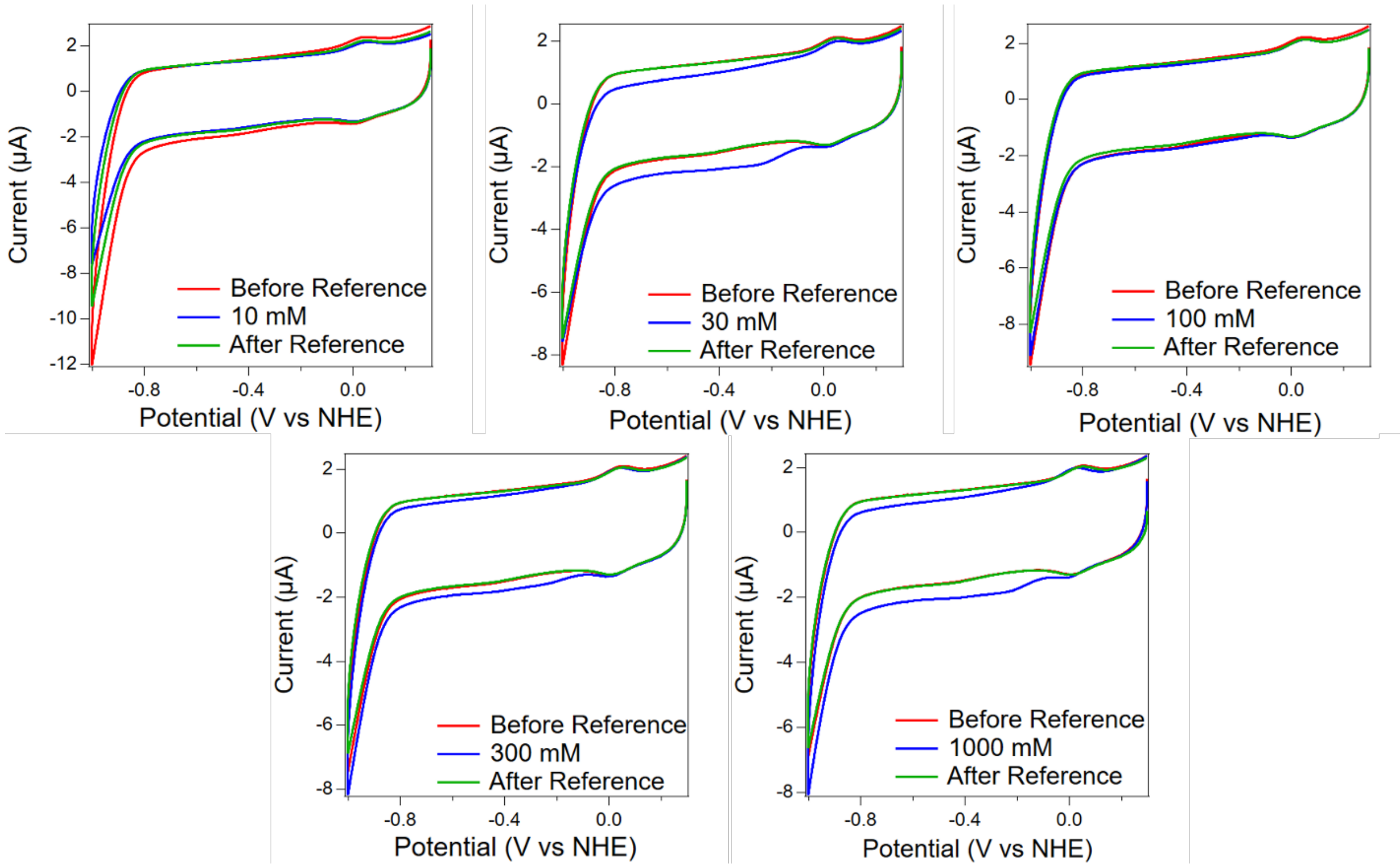

Figure S17. MES pH 5.5 Trial 2. Cyclic voltammograms of NiRd covalently attached to electrode surface with a unique film from other trials. For each buffer concentration surveyed, a reference in a 100 mM buffer solution was recorded before (red trace) and after (green trace) the experiment was conducted in the buffer concentration of interest (blue trace).

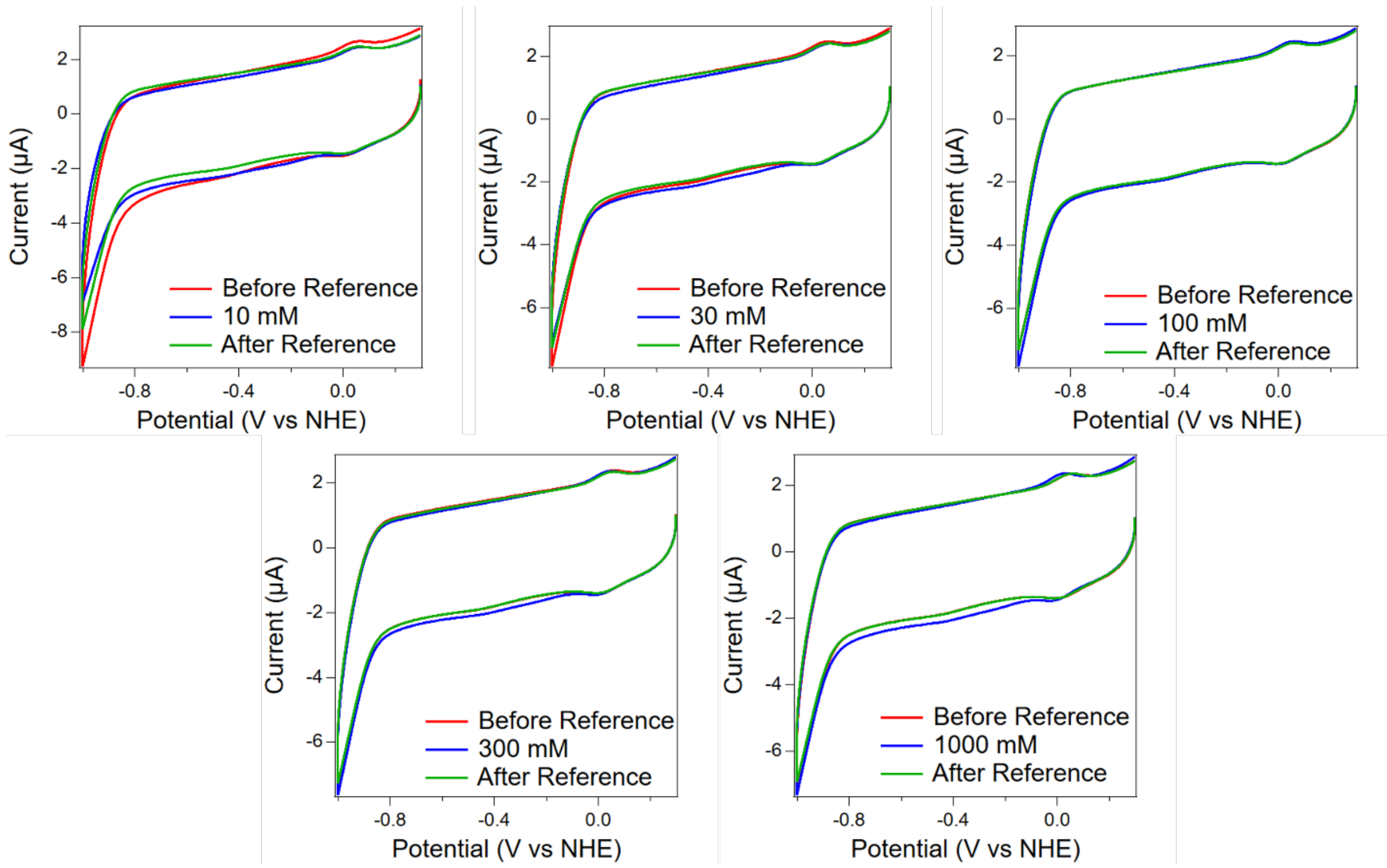

Figure S18. MES pH 5.5 Trial 3. Cyclic voltammograms of NiRd covalently attached to electrode surface with a unique film from other trials. For each buffer concentration surveyed, a reference in a 100 mM buffer solution was recorded before (red trace) and after (green trace) the experiment was conducted in the buffer concentration of interest (blue trace).

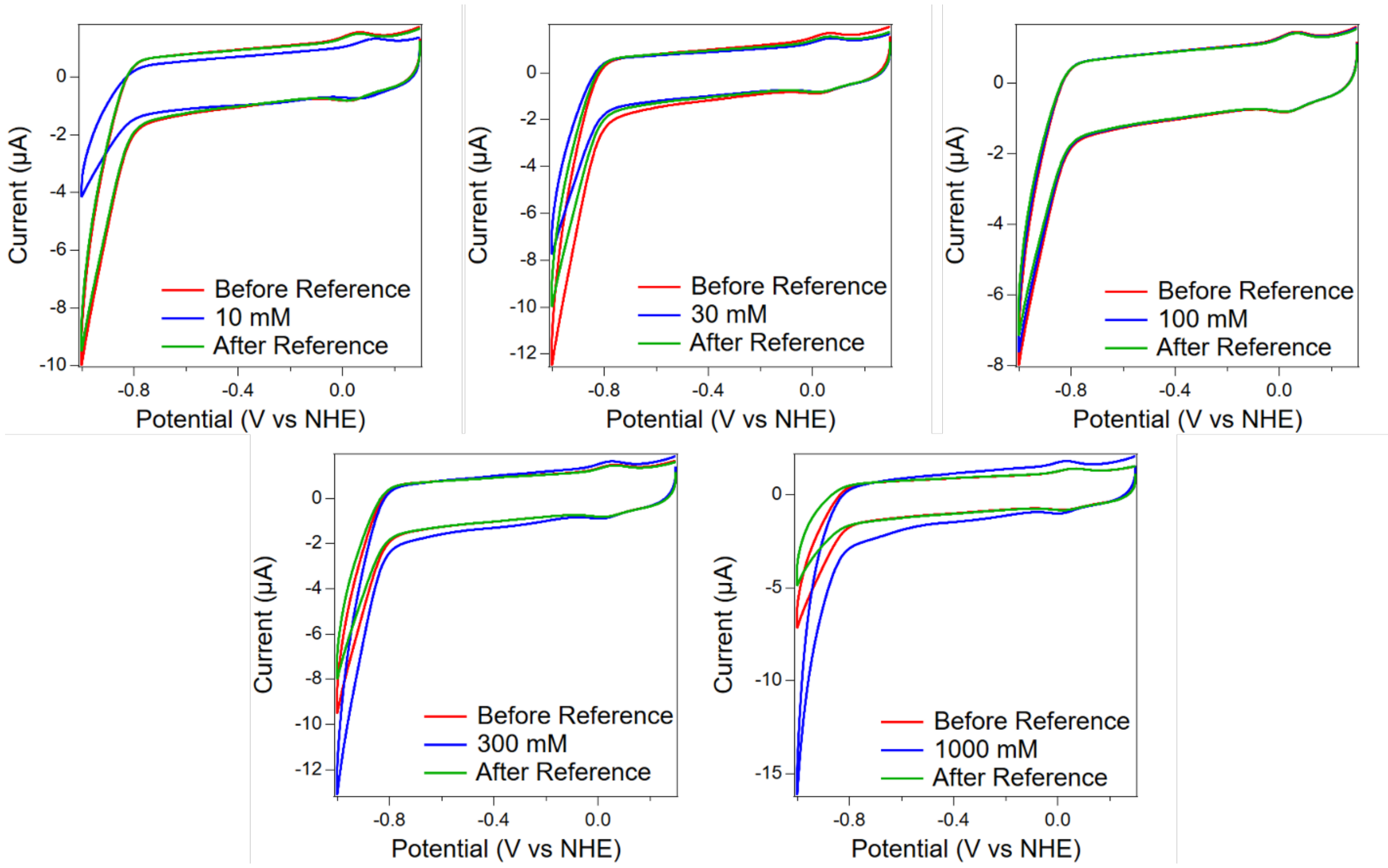

Figure S19. Pyridine pH 4.5 Trial 1. Cyclic voltammograms of NiRd covalently attached to electrode surface with a unique film from other trials. For each buffer concentration surveyed, a reference in a 100 mM buffer solution was recorded before (red trace) and after (green trace) the experiment was conducted in the buffer concentration of interest (blue trace). In solutions of 1000 mM pyridine, the catalytic current has a shape unlike typical NiRd HER, which prevented its usual analysis and thus was not recorded further. Additionally, the electrochemistry of pyridinium has been previously explored and has an irreversible reduction event around -800 mV vs NHE, which matches what we observe in this voltammogram.[6]

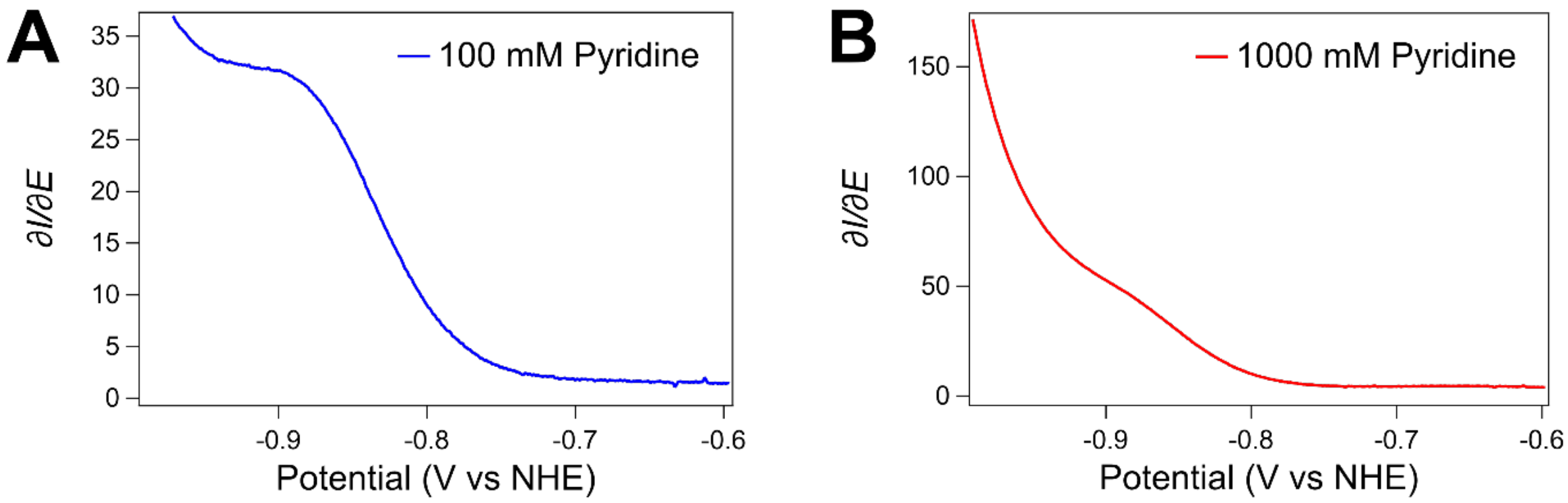

Figure S20. Pyridine Catalytic Wave Derivatives. (A) First derivative of the cathodic portion of a representative CV in 100 mM pyridine (blue trace). The first derivative has a typical sigmoidal shape characteristic of NiRd catalysis. (B) First derivative of the cathodic portion of a representative CV in 1000 mM pyridine (red trace). There is a lack of clearly defined sigmoidal shape of NiRd catalysis. Additionally, the current and $\frac{\partial I}{\partial E}$ values are much higher than expected of NiRd and likely correspond with electrochemistry of pyridinium.[6] Within the pH range studied, large amounts of pyridinium will always be present precluding the study of this specific concentration.

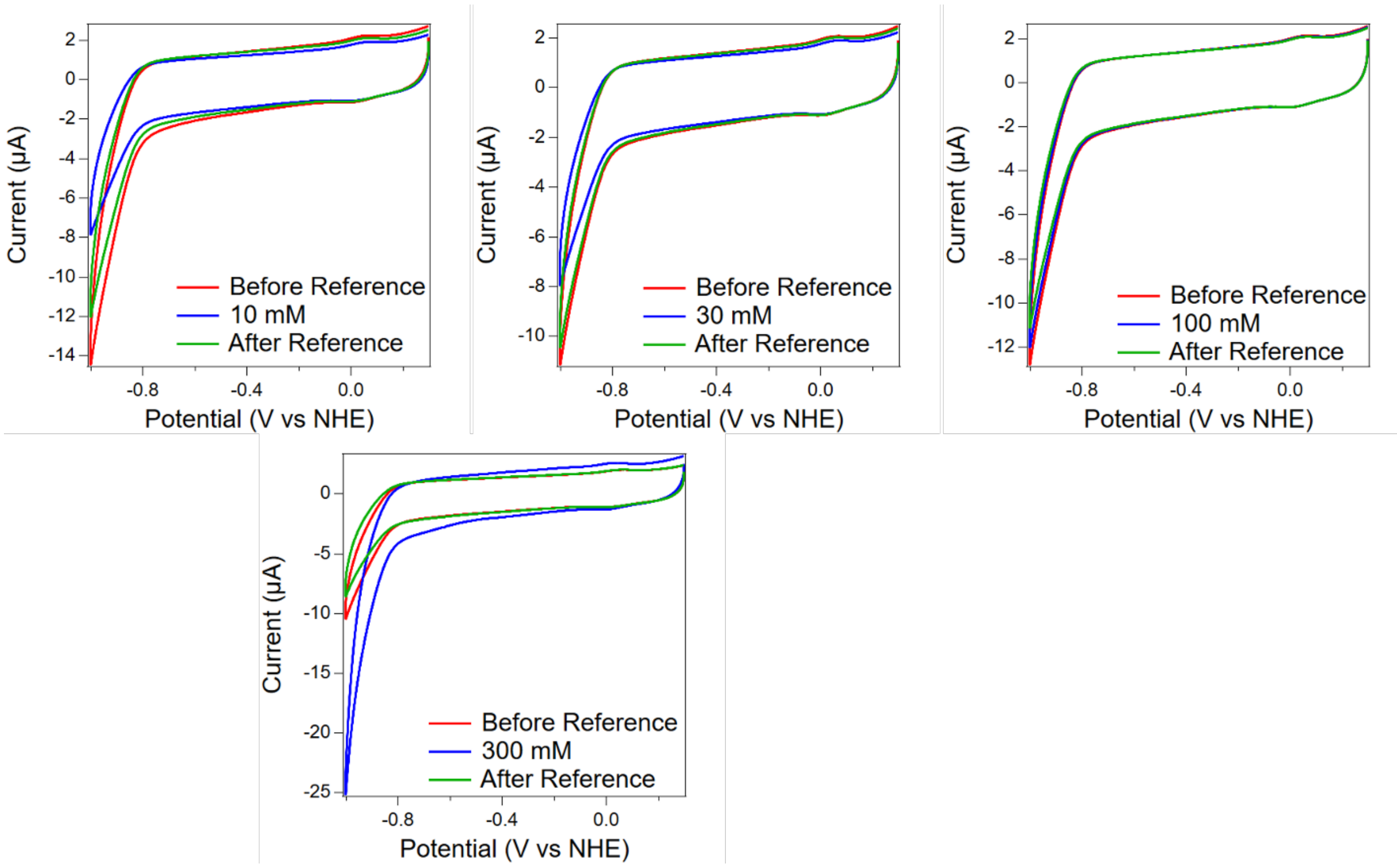

Figure S21. Pyridine pH 4.5 Trial 2. Cyclic voltammograms of NiRd covalently attached to electrode surface with a unique film from other trials. For each buffer concentration surveyed, a reference in a 100 mM buffer solution was recorded before (red trace) and after (green trace) the experiment was conducted in the buffer concentration of interest (blue trace).

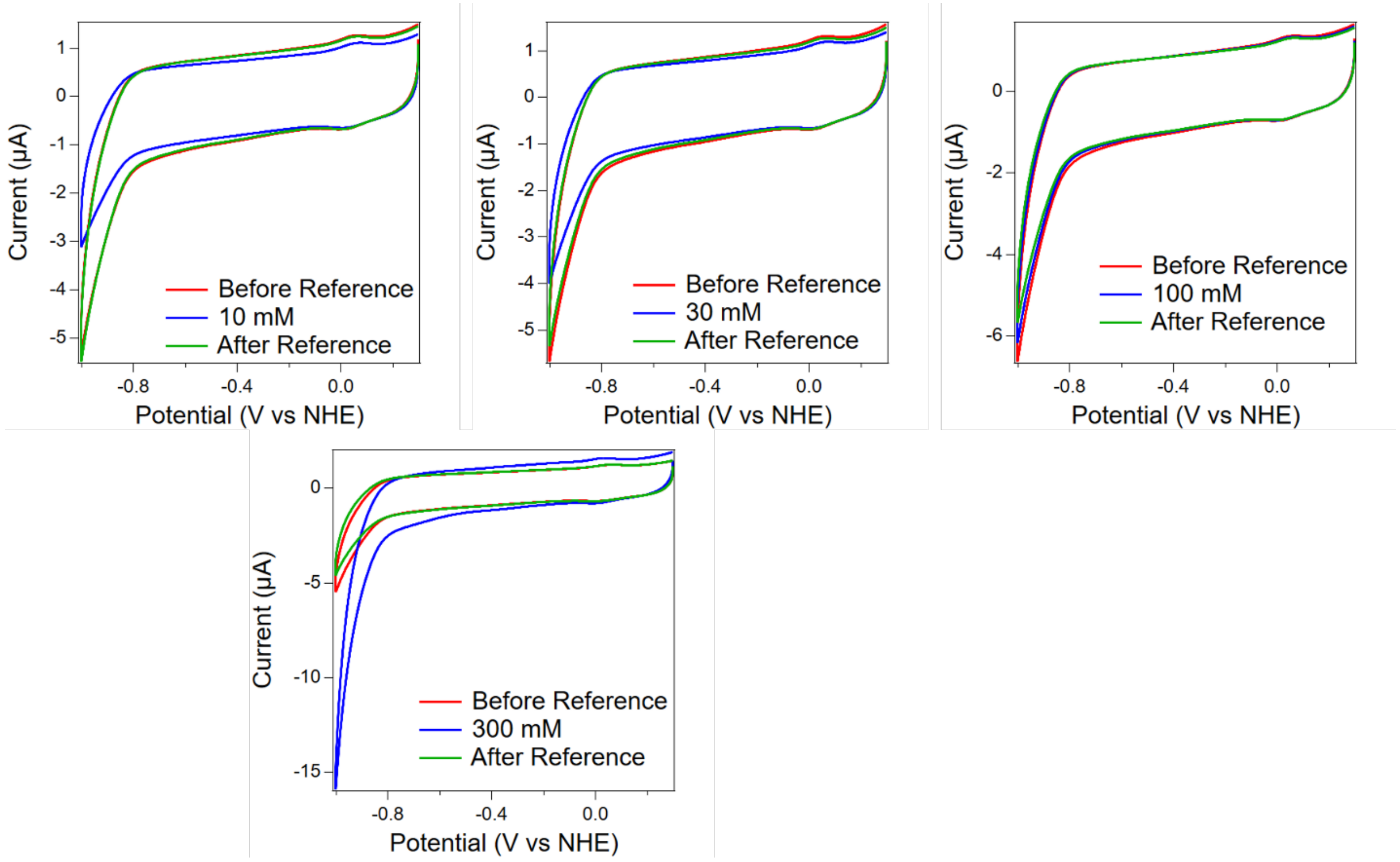

Figure S22. Pyridine pH 4.5 Trial 3. Cyclic voltammograms of NiRd covalently attached to electrode surface with a unique film from other trials. For each buffer concentration surveyed, a reference in a 100 mM buffer solution was recorded before (red trace) and after (green trace) the experiment was conducted in the buffer concentration of interest (blue trace).

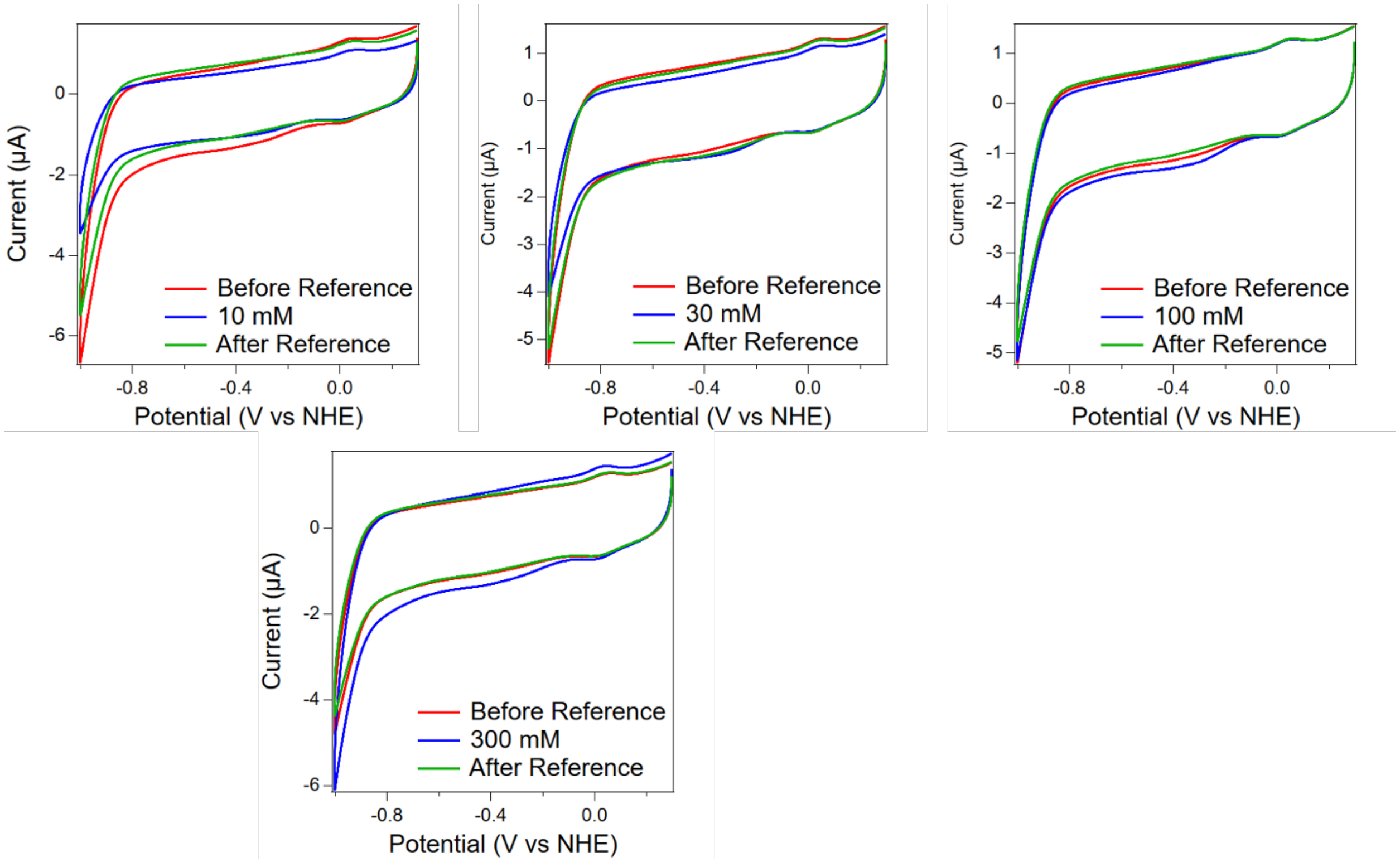

Figure S23. Pyridine pH 5.5 Trial 1. Cyclic voltammograms of NiRd covalently attached to electrode surface with a unique film from other trials. For each buffer concentration surveyed, a reference in a 100 mM buffer solution was recorded before (red trace) and after (green trace) the experiment was conducted in the buffer concentration of interest (blue trace).

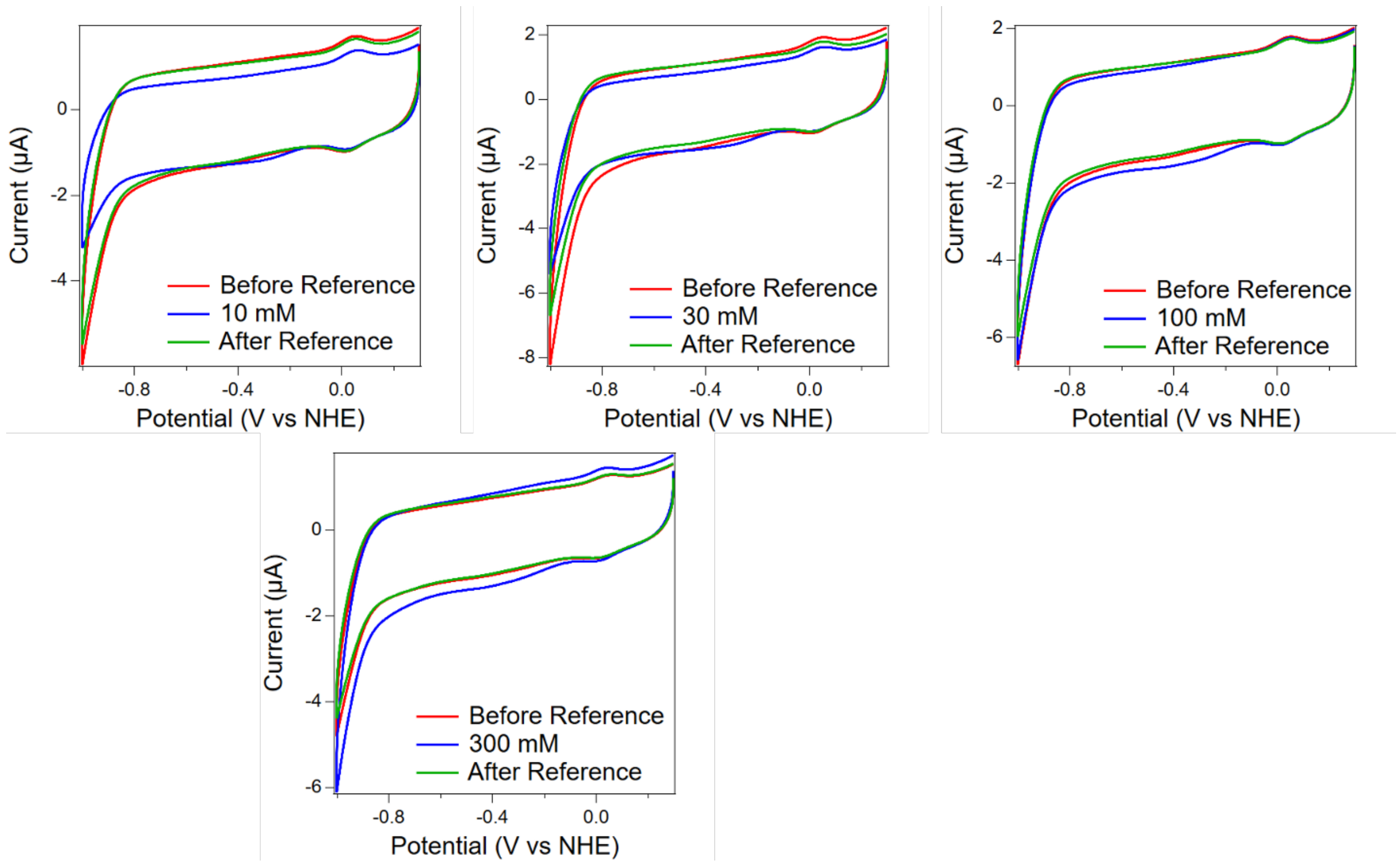

Figure S24. Pyridine pH 5.5 Trial 2. Cyclic voltammograms of NiRd covalently attached to electrode surface with a unique film from other trials. For each buffer concentration surveyed, a reference in a 100 mM buffer solution was recorded before (red trace) and after (green trace) the experiment was conducted in the buffer concentration of interest (blue trace).

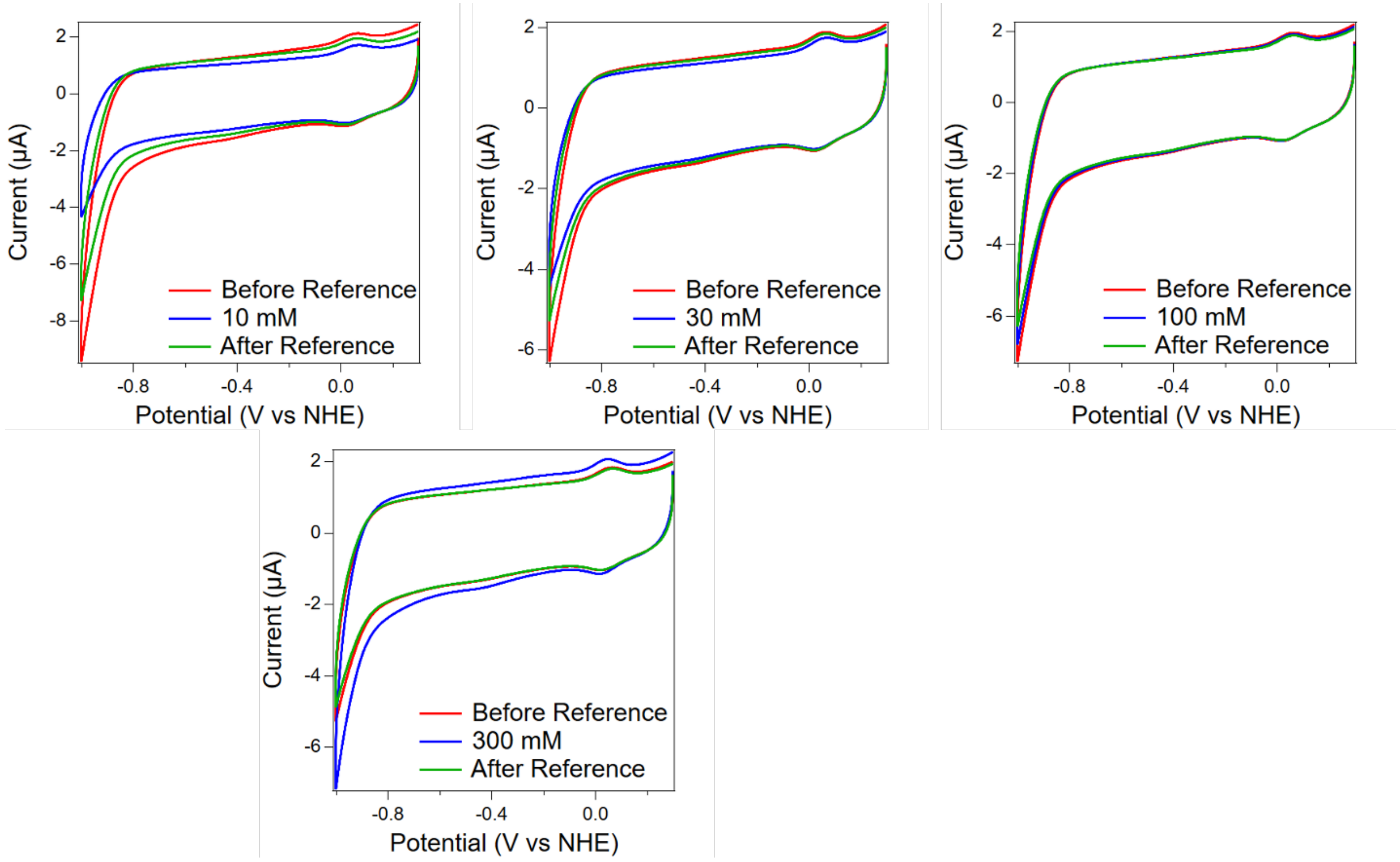

Figure S25. Pyridine pH 5.5 Trial 3. Cyclic voltammograms of NiRd covalently attached to electrode surface with a unique film from other trials. For each buffer concentration surveyed, a reference in a 100 mM buffer solution was recorded before (red trace) and after (green trace) the experiment was conducted in the buffer concentration of interest (blue trace).

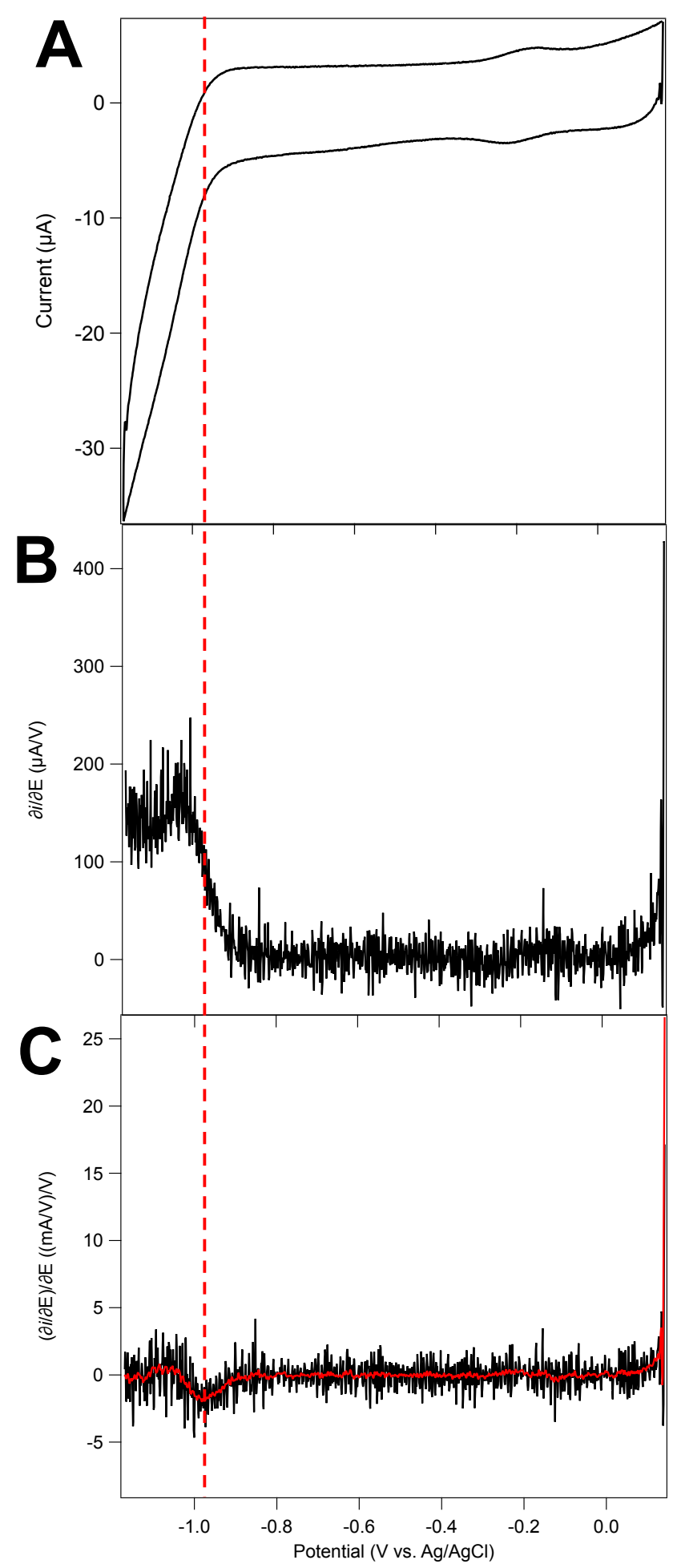

Figure S26. Procedure used to identify the onset potential of NiRd. (**A**) CV of a 76:24 mixture of Ni^{II}Rd/Fe^{II}Rd in 150 mM OAc, pH 3.5. (**B**) The first derivative of the CV reveals a sigmoid-like feature. (**C**) The second derivative (black), shown with noise reduced using a 20-point box-smoothing function, exhibits a negative peak (red). The minimum of this feature (equivalent to the inflection point of the first derivative) is the point defined as the onset potential for NiRd catalysis (dotted line).

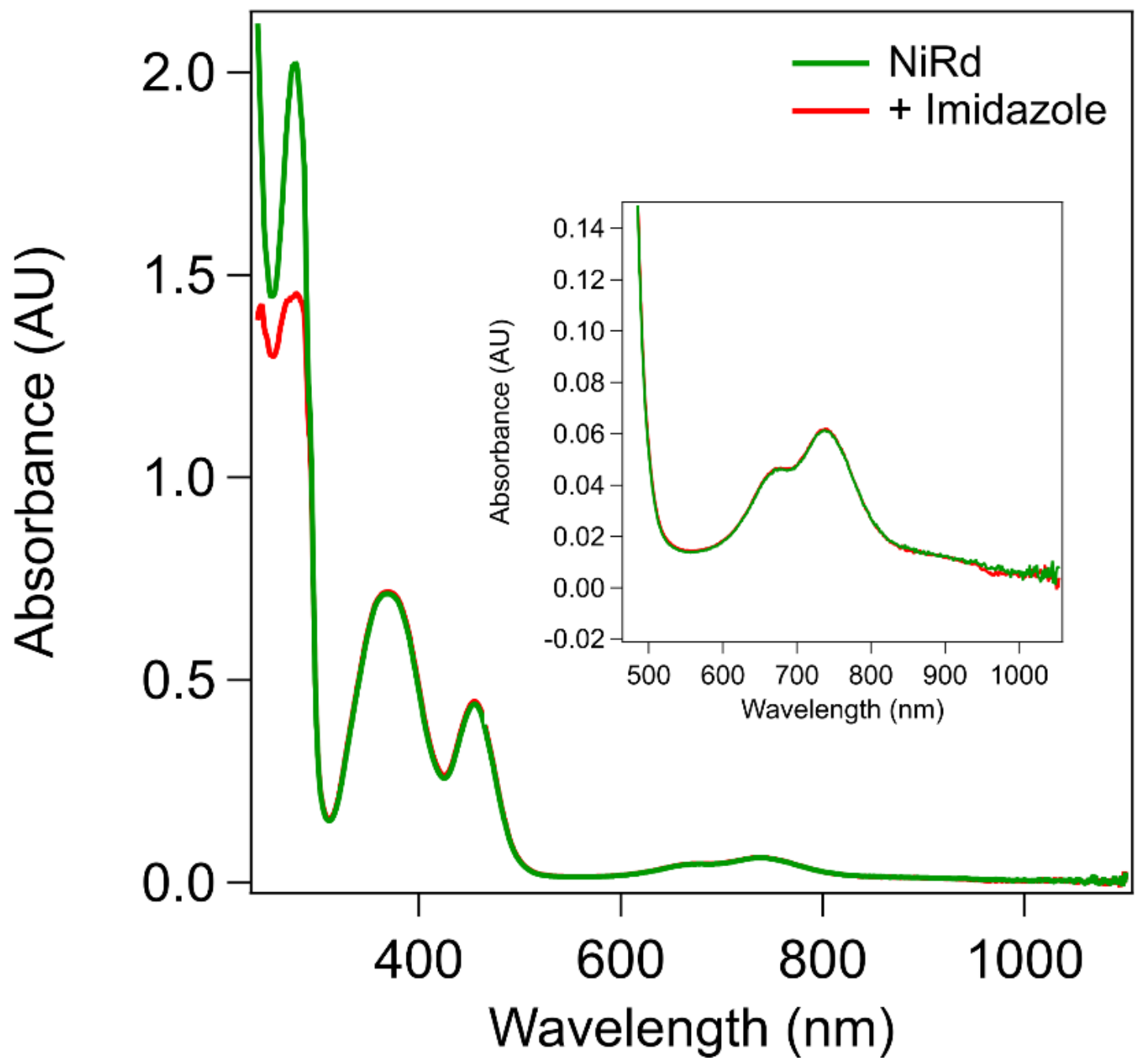

Figure S27. UV-Vis spectrum of 132 μM NiRd (green) with 100 mM added imidazole (red) at pH 4.5. (Inset) Zoomed-in view of the d-d transitions of NiRd.

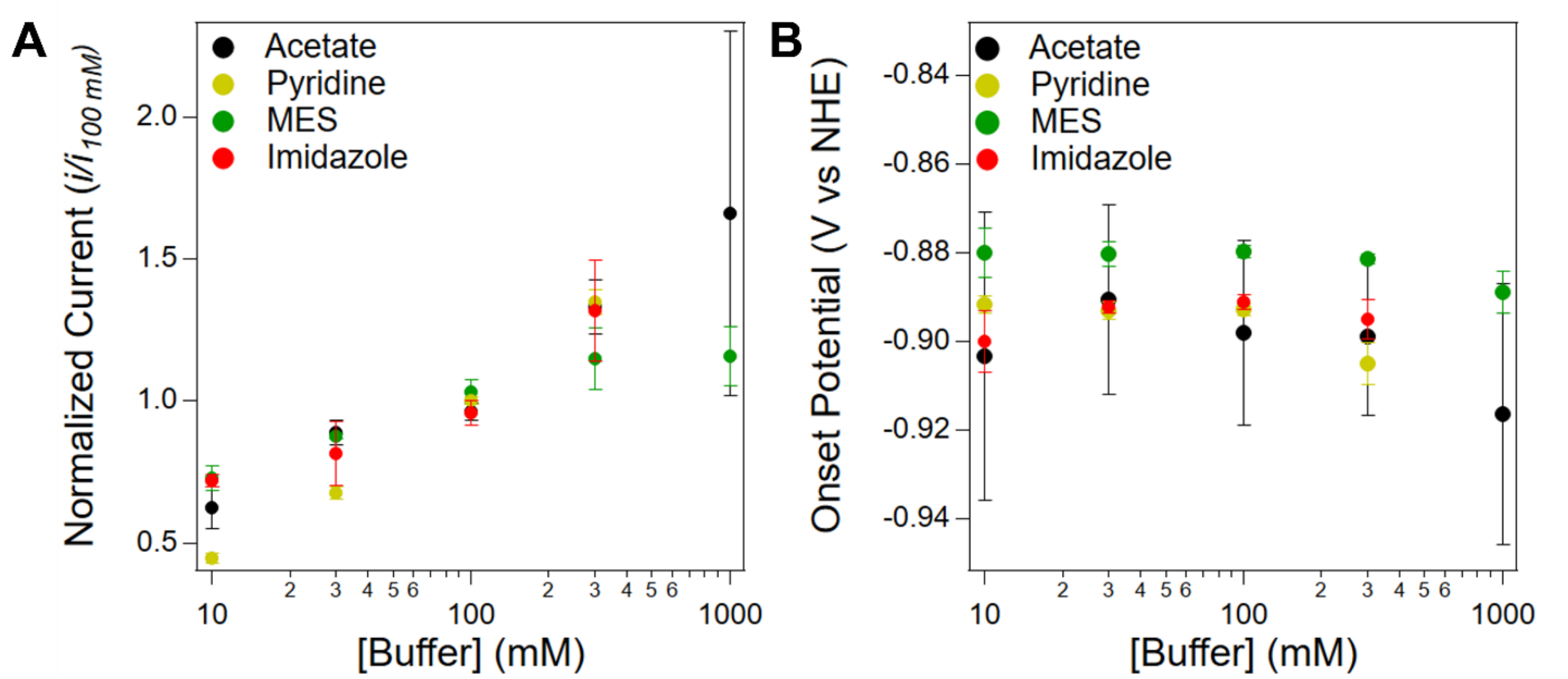

Figure S28. Normalized current (A) and onset potential (B) of NiRd as a function of buffer concentration at pH 5.5.

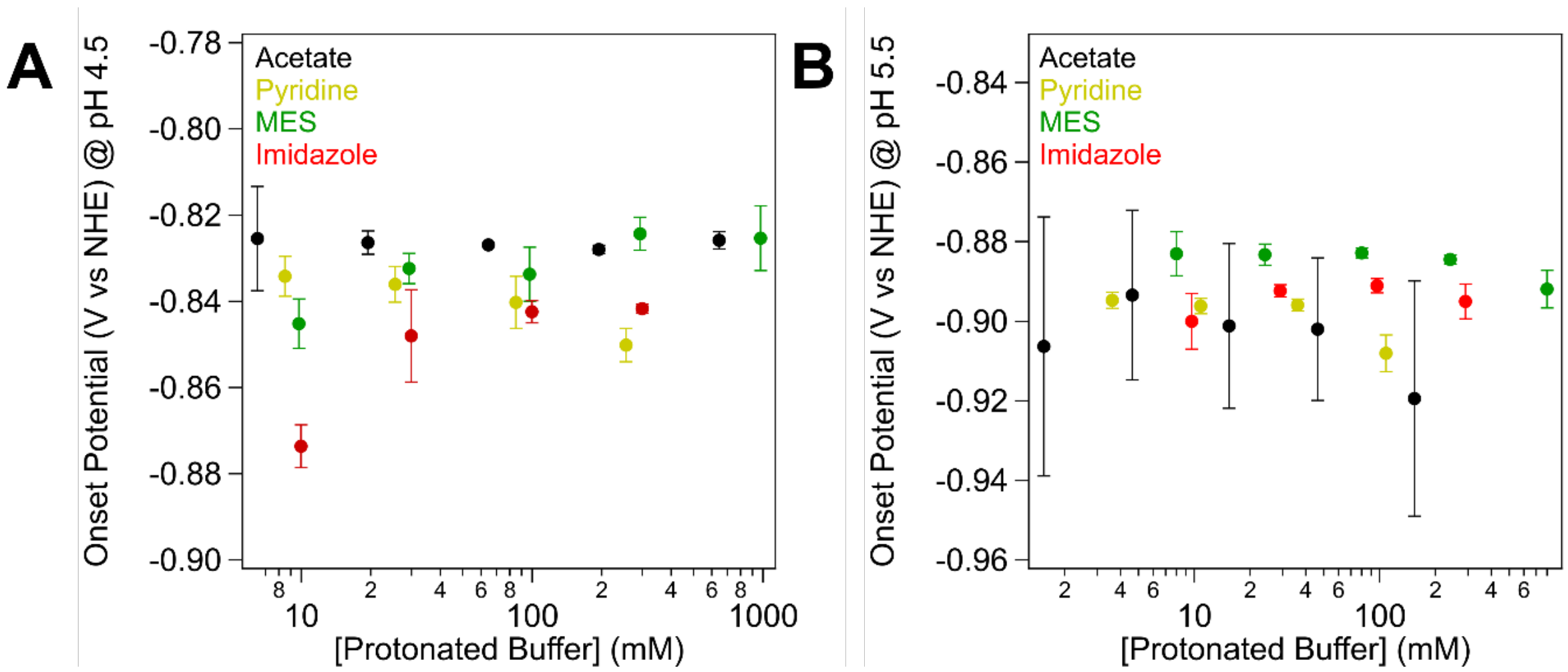

Figure S29. Onset potential as a function of proton donor concentration (protonated buffer molecules). (**A**) Onset potential as a function of the conjugate acid present at pH 4.5. (**B**) Onset potential as a function of the conjugate acid present at pH 5.5. In all cases, these plots yield the exact same trends as total buffer concentration, only the concentrations values slightly shift. These results thus demonstrate that NiRd is reacting with the conjugate acid of the buffer.

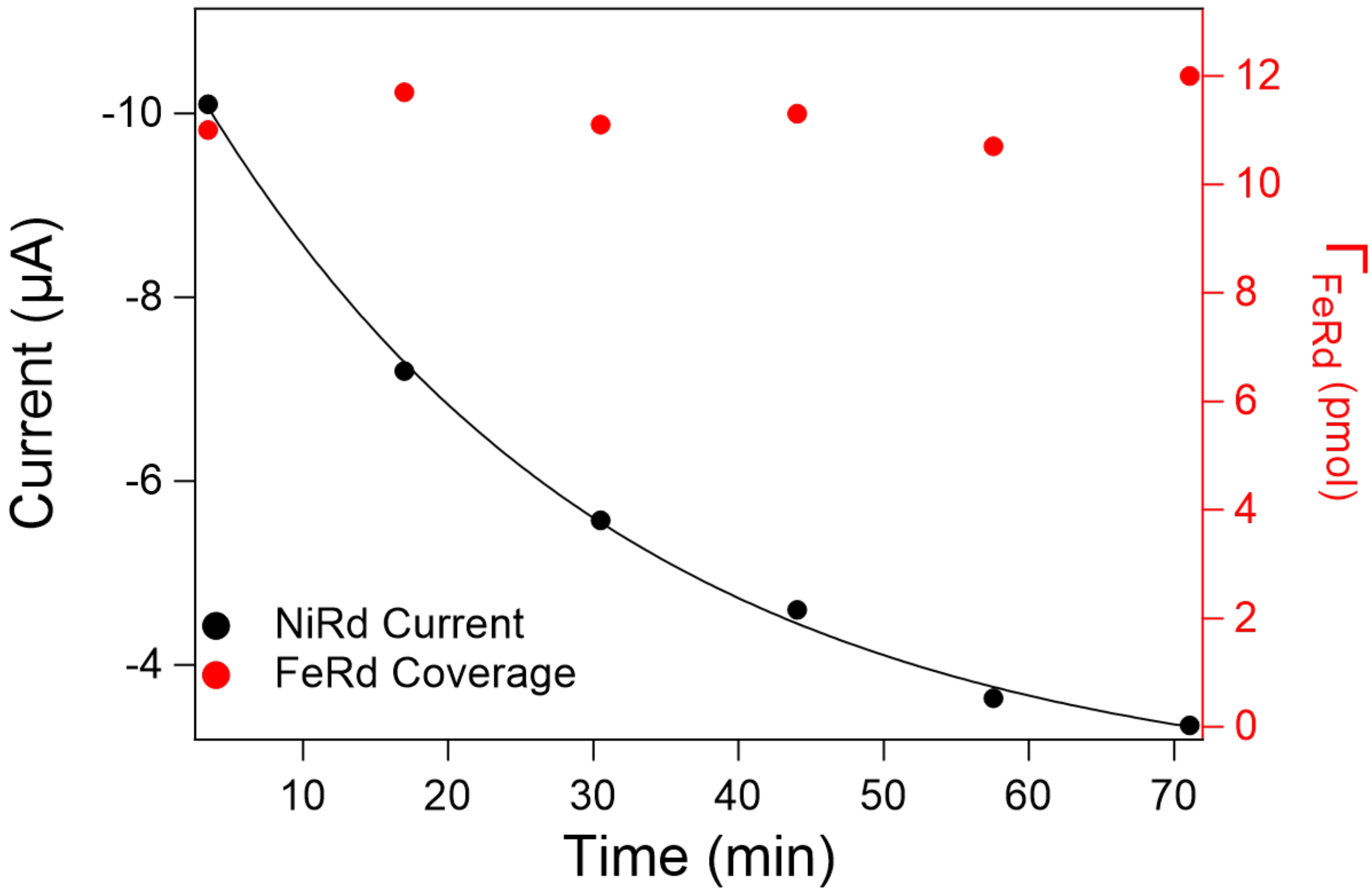

Figure S30. NiRd current over time**.** Measured NiRd current at the potential of analysis over time compared to FeRd coverage. The FeRd electroactive coverage can be directly measured via the reversible $Fe^{II/III}$ couple and shows that the same film of FeRd when exposed to experimental electrochemical conditions retains the same electroactive coverage throughout the experimental timescale. However, prolonged exposure to the experimental electrochemical conditions of the same film of NiRd shows that the current from NiRd attenuates overtime. This has complicated turnover frequency analysis. To overcome this, the procedure outlined in the materials and methods was followed. With this approach, the NiRd current was fit to an exponential function (black line) which yielded good agreement to the data with a time constant of approximately 30 minutes. The current measurements were all taken in the same 100 mM reference solution. The time recorded was the time of applied current and not any time spent rinsing electrodes or transferring between solutions.

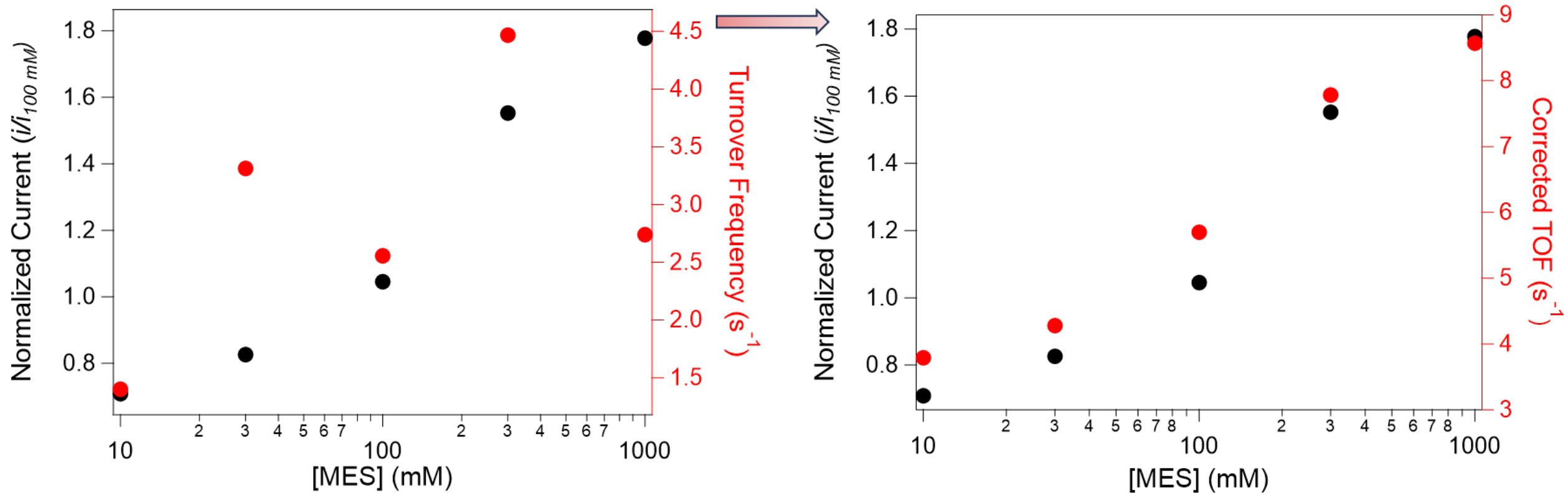

Figure S31. Correction Factor Application. (Left) Both the normalized current (black circles) and uncorrected turnover frequency (red circles) as a function of buffer concentration. (Right) The normalized current and corrected turnover frequency as a function of buffer concentration. As is shown in the left, the uncorrected turnover frequencies are challenging to assess, as they show no clear trend with buffer concentration. However, this is unexpected as turnover frequency is a current value normalized by catalyst concentration. By accounting for NiRd inactivation during the experimental timescale as outlined in Figure S15, the turnover frequency can be corrected for catalyst inactivation. The result of this correction is much better agreement in the turnover frequency trends with buffer concentration as compared to normalized current trends.

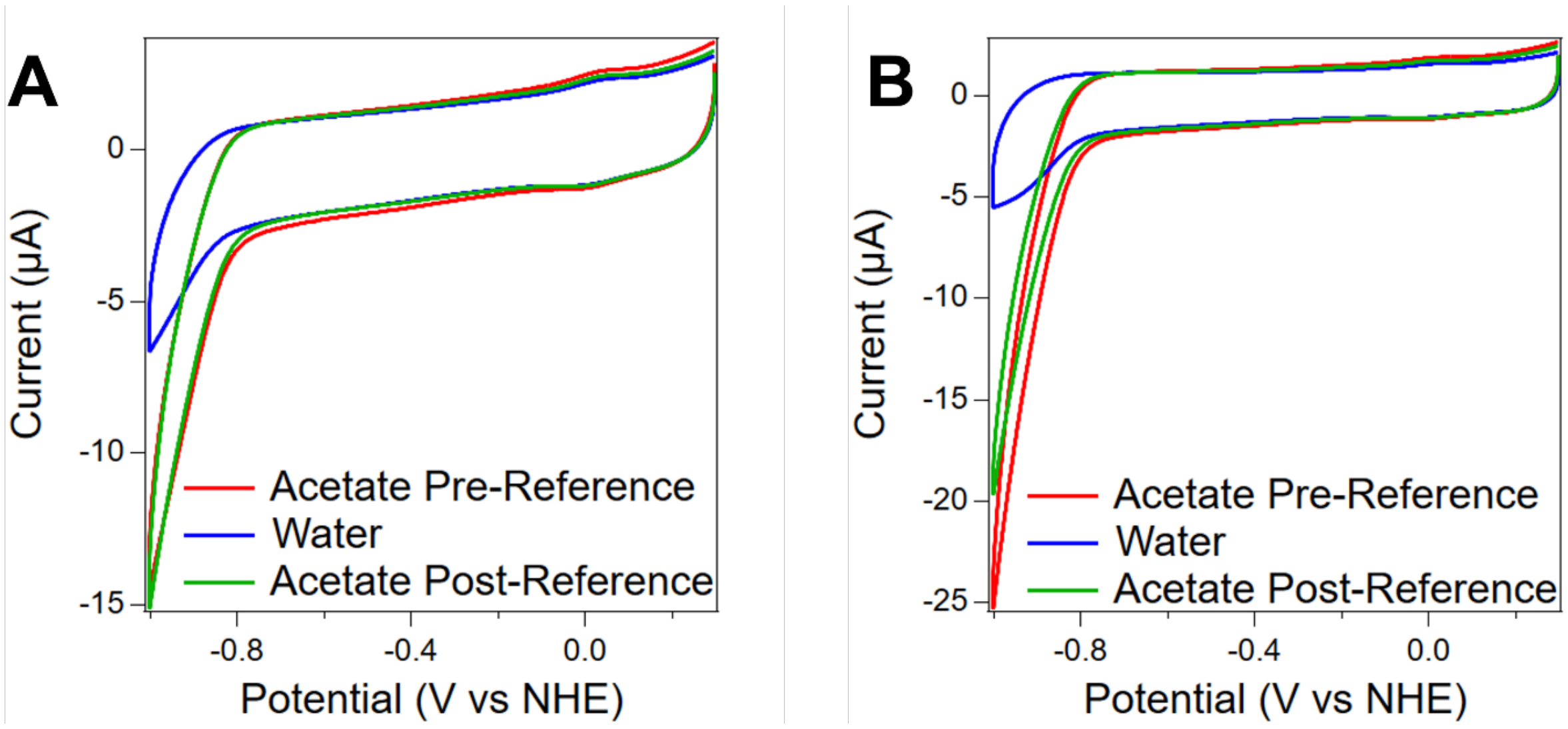

Figure S32. CVs of NiRd in water at pH 4.5 where (A) is trial 2 and (B) is trial 2 (blue traces). The other trial is presented in Figure 5A. In all cases, reference CVs in 100 mM acetate at the same pH was used to determine normalized currents (red and green traces). Each trial represents a unique film.

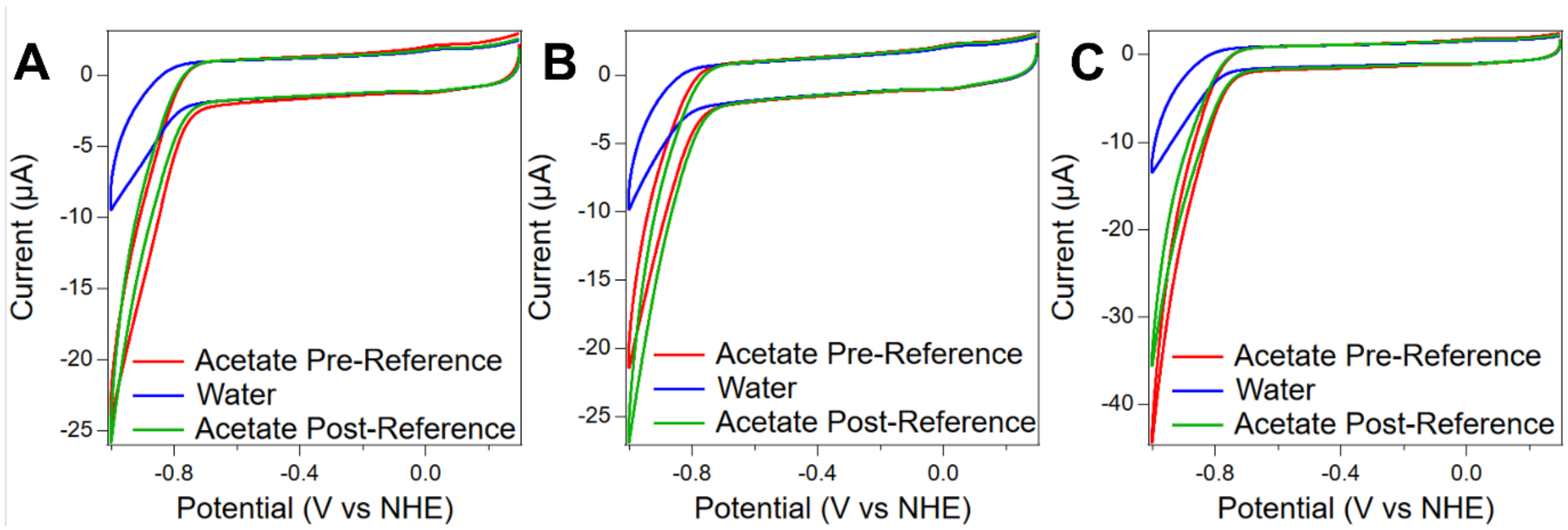

Figure S33. CVs of NiRd in water at pH 3.5 where (A) is trial 1, (B) trial 2, and (C) trial 3 (blue traces). In all cases, reference CVs in 100 mM acetate at the same pH was used to determine normalized currents (red and green traces). Each trial represents a unique film.

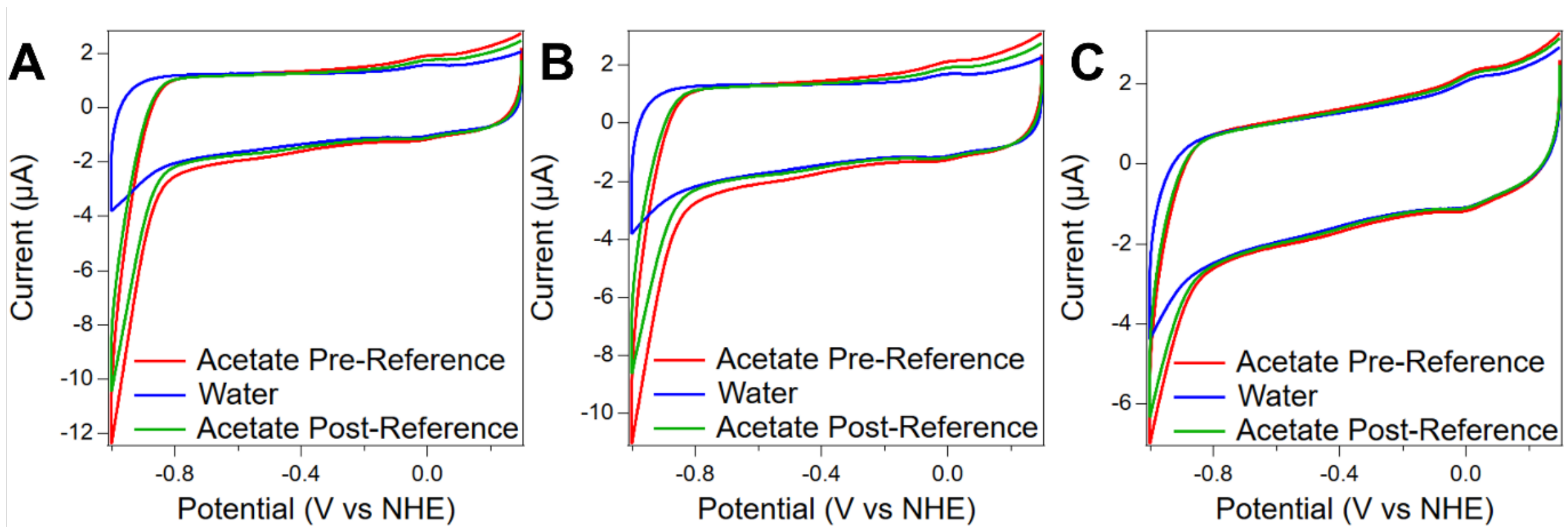

Figure S34. CVs of NiRd in water at pH 5.5 where (A) is trial 1, (B) trial 2, and (C) trial 3 (blue traces). In all cases, reference CVs in 100 mM acetate at the same pH was used to determine normalized currents (red and green traces). Each trial represents a unique film.

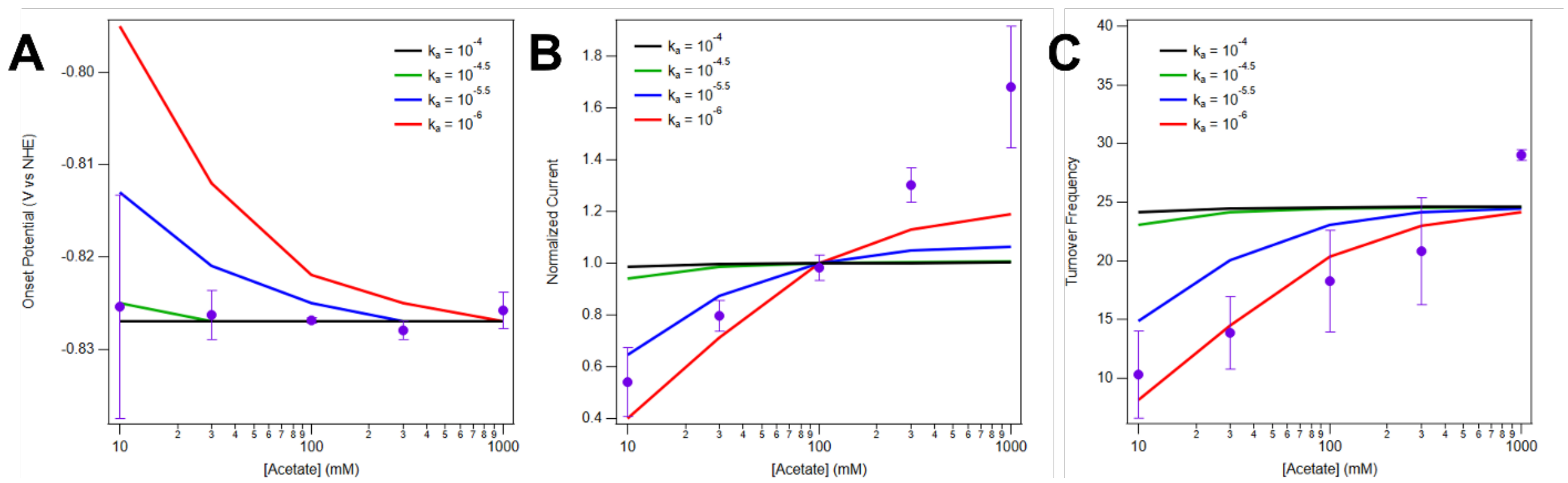

Figure S35. Simulated (A) onset potential, (B) normalized current, and (C) turnover frequency for NiRd catalysis at pH 4.5 in acetate buffer. The k_a value was varied to match the onset potential while k_1 was varied to match the normalized current and turnover frequency with a k_a of 10^{-6} and k_1 of 15 satisfying the greatest amount of experimental data.

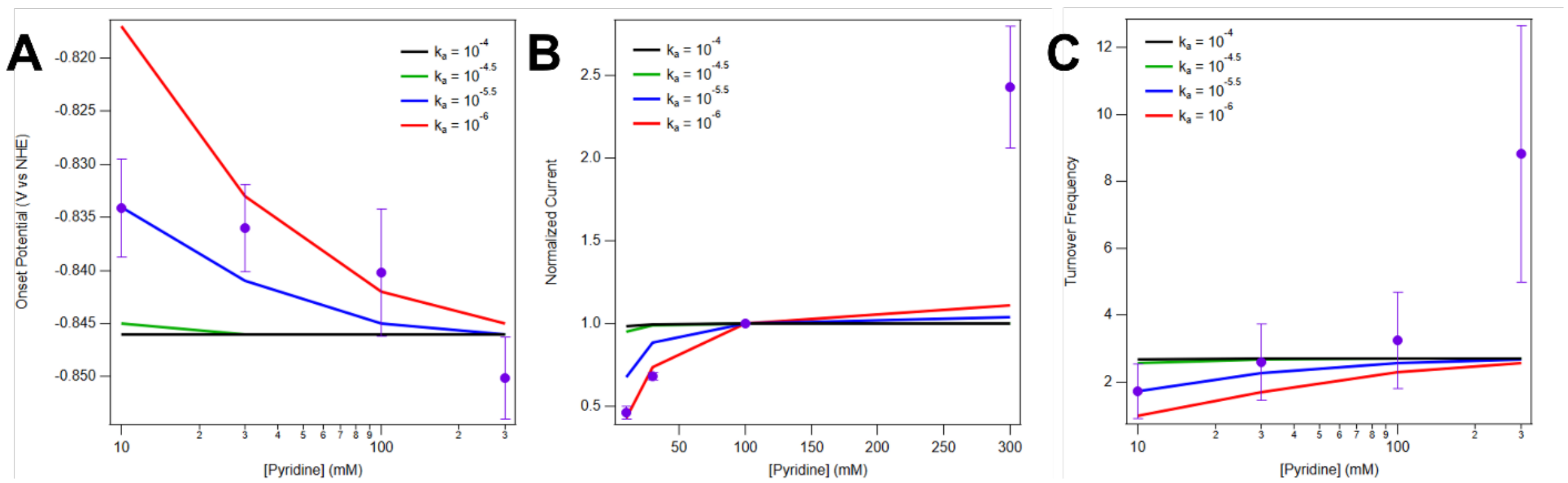

Figure S36. Simulated parameters of NiRd catalysis at pH 4.5 in pyridine with (A) onset potential, (B) normalized current, and (C) turnover frequency. . The ka value was varied to match the onset potential while k1 was varied to match the normalized current and turnover frequency with a k_a of 10^{-6} and k1 of 17 satisfying the most experimental data.

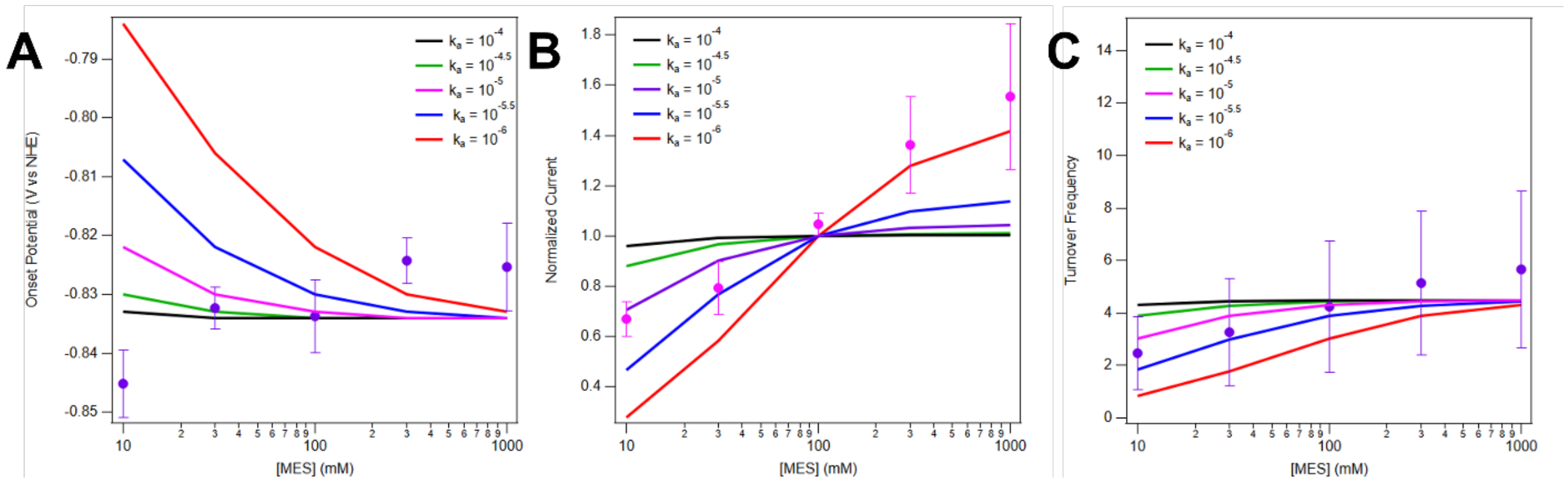

Figure S37. Simulated parameters of NiRd catalysis at pH 4.5 in MES with (A) onset potential, (B) normalized current, and (C) turnover frequency. . The ka value was varied to match the onset potential while k1 was varied to match the normalized current and turnover frequency with a k_a of 10^{-5} and k1 of 55 satisfying the most experimental data.

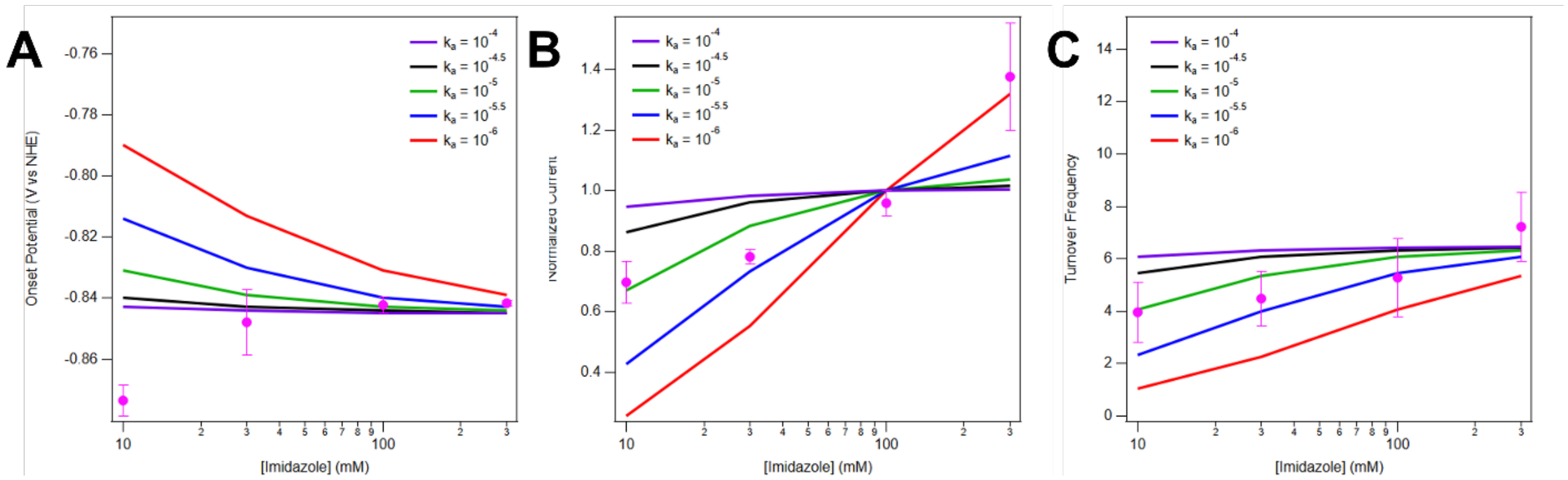

Figure S38. Simulated parameters of NiRd catalysis at pH 4.5 in imidazole with (A) onset potential, (B) normalized current, and (C) turnover frequency. . The ka value was varied to match the onset potential while k1 was varied to match the normalized current and turnover frequency with a k_a of 10^{-5} and k1 of 60 satisfying the most experimental data.

Table S1. Onset potentials for all samples at all pH values.

Onset Potentials (V vs. NHE)								
	Acetate (pK_a 4.76)		Pyridine (pK_a 5.23)		MES (pK_a 6.15)		Imidazole (pK_a 6.95)	
[Buffer] (mM)	pH 4.5	pH 5.5	pH 4.5	pH 5.5	pH 4.5	pH 5.5	pH 4.5	pH 5.5
10	-0.825 ± 0.012	-0.906 ± 0.033	-0.834 ± 0.005	-0.895 ± 0.002	-0.845 ± 0.006	-0.883 ± 0.006	-0.874 ± 0.005	-0.900 ± 0.007
30	-0.826 ± 0.003	-0.893 ± 0.021	-0.836 ± 0.004	-0.896 ± 0.002	-0.832 ± 0.003	-0.883 ± 0.003	-0.848 ± 0.011	-0.892 ± 0.002
100	-0.827 ± 0.0003	-0.901 ± 0.021	-0.840 ± 0.006	-0.896 ± 0.001	-0.834 ± 0.006	-0.883 ± 0.001	-0.842 ± 0.003	-0.891 ± 0.002
300	-0.828 ± 0.001	-0.902 ± 0.018	-0.850 ± 0.004	-0.908 ± 0.005	-0.824 ± 0.004	-0.884 ± 0.001	-0.842 ± 0.001	-0.895 ± 0.004
1000	-0.826 ± 0.002	-0.919 ± 0.030	–	–	-0.825 ± 0.008	-0.892 ± 0.005	–	–

Appendix I: Sample Matlab Script and Description of Fitting Process

The following script was used for modeling the electrochemical behavior of NiRd in the buffers used in this study. This script has been extensively characterized and used in the past for describing this system[3,4] and other rubredoxin variants[7]. All parameters have been kept the same, or modified in the same fashion to replicate experimental data as described earlier. In this work, the k_a parameter was explored.

k_a is related to the pK_a of NiRd and the buffer by the following equilibria:

We begin with the simple equilibria between water with the buffer and NiRd,

$$HA + H_2O \rightleftharpoons H_3O^+ + A^-$$

$$K_{a,\ buffer} = \frac{[H_3O^+][A^-]}{[HA][H_2O]}$$

$$NiRd - SH^+ + H_2O \rightleftharpoons H_3O^+ + NiRd$$

$$K_{a,\ NiRd} = \frac{[H_3O^+][NiRd]}{[NiRd - SH^+][H_2O]}$$

The above simpler equilibria can be used to derive the pK_a of the system $pK_{a,\ sys}$ through a combination of the $pK_{a,\ NiRd}$ and $pK_{a,\ buffer}$:

$$NiRd + HA \rightleftharpoons NiRd - SH^+ + A^-$$

$$K_{sys} = \frac{1}{K_{a,\ NiRd}} \times K_{a,\ buffer} = \frac{[NiRd - SH^+][A^-]}{[HA][NiRd]}$$

Taking the negative log of each side of the equation results in:

$$pK_{a,\ sys} = pK_{a,\ buffer} - pK_{a,\ NiRd}$$

Where the k_a and k_b presented are equal to the system pK_a by:

$$pK_{a,sys} = \frac{k_{a,sys}}{k_{b,sys}}$$

Sample Matlab Script

```
% All concentrations currently in nM

% Define pH and buffer pKa!
pH = 5.5;
pKa = 4.76;

% Constants
Eo = -0.751;      % Reduction Potential in V
Ei = 0;           % Initial Potential in V
v = -0.1;         % Scan rate in V/s
a = 0.5;          % Alpha
F = 96485.33289; % Faraday's Constant in C/mol
R = 8.314;        % Gas Constant in J/mol*T
T = 298;          % Temp in Kelvin
```

```
f = F/(R * T);   % Easier Constant for BV Equations
NiConc = 0.01;   % in nM

% Define time/potential parameters
tfinal = -1.1/v; % numerator is final/lowest potential
tstep = 0.01;
tspan = 0:tstep:tfinal;

% Define oxidation/reduction rates as functions
x = 4:0.1:12; % dispersion distances
kred  = @(t, x) 225000 * exp(-x) * exp(-(a * f) * ((Ei + v*t) - Eo));
kox   = @(t, x) 225000 * exp(-x) * exp(((1-a) * f) * ((Ei + v*t) - Eo));

% Set resolution of solutions
opt = odeset('Reltol', 1e-11, 'Abstol', 1e-13);

% Define the pH range (number of LSVs)
Bufferrange = [10, 30, 100, 300, 1000]; % in mM

%Preallocate for speed
Time = cell(1, length(x));
Concentrations = cell(1, length(x));
TOFList = cell(1, length(Bufferrange));
PotentialOnsetList = cell(1, length(Bufferrange));
CurrentList = cell(1, length(Bufferrange));

for BufferConc = 1:length(Bufferrange)

%   Determine Protonated and Unprotonated Values
BufferM = Bufferrange(BufferConc)*1e6;
A      = BufferM/(10^(pKa-pH)+1);
HA      = BufferM - A;

for i = 1:length(x)
   d = x(i);

%      Initial Concentrations (nM)
      C0 = [NiConc, 0, 0, 0, 0, 0];

%      Solve the ODEs/mechanism
      [Time{i}, Concentrations{i}] = ode15s(@(t, C) mechanism(t, C, kred, kox, d, HA, A), tspan,
C0, opt);

end

% Define arrays for ODE solutions (faster computations)
M = length(x);
AvgConc = zeros(length(tspan),length(C0));

% Average solutions
for i = 1:M
```

```
    AvgConc = AvgConc + Concentrations{i}/M;
end

% Define outputs/axes
%LSV
Potential = Time{i}*v;
Potential_Current = Potential(2:end);

ChargeDif = diff(AvgConc(:,6))/tstep;
Current = ChargeDif*(F/1e9);

CurrentList{BufferConc} = Current;

% figure(1);
% plot(Potential_Current, Current);
xlabel('Potential(V vs NHE)');
ylabel('Current (A)');


%First Derivative
% figure(2);
CurrentDif = diff(Current)/tstep;
Potential_CurrentDif = Potential(3:end);
% plot(Potential_CurrentDif, CurrentDif);
xlabel('Potential (V vs NHE)');
ylabel('dI/dE')



%Second Derivative
% figure(3)
Current2Dif = diff(CurrentDif)/tstep;
Potential_2Dif = Potential(4:end);
% plot(Potential_2Dif, Current2Dif)


%Third Derivative
% Current3Dif = diff(Current2Dif)/tstep;
% Potential_3Dif = Potential(5:end);

%Turnover Frequency
minimumval = min(Current2Dif);

PotentialOnset = Potential_2Dif(find(Current2Dif == minimumval));
PotentialOnsetList{BufferConc} = PotentialOnset; %Look at specific Onset Potentials


PotentialAnalysis = PotentialOnset - 0.1;
CurrentAnalysis   = Current(find(abs(Potential - PotentialAnalysis) < 1e-6));
TOF = -CurrentAnalysis/(NiConc*2*(F/1e9));
TOFList{BufferConc} = TOF; %Look at specific TOFs
```

```
end

TOFList = transpose(TOFList);
PotentialOnsetList = transpose(PotentialOnsetList);

%Plotting Onset Potential
% figure(4);
% PotentialOnsetList = cell2mat(PotentialOnsetList);
% scatter(Bufferrange, PotentialOnsetList);
% xlabel('[Buffer] (mM)');
% ylabel('Onset Potential (V vs NHE)');


%Plotting Turnover Frequency
% figure(5);
% TOFList = cell2mat(TOFList);
% scatter(Bufferrange, TOFList);
% xlabel('[Buffer] (mM)');
% ylabel('Turnover Frequency (1/s)');



function dC = mechanism(t, C, kred, kox, x, HA, A)

%Rate Constants
k1 = 15;
k2 = 1;
pKaNi = 2.5;
pKaHA = 4.76;
pKasystem = pKaHA - pKaNi;

%Protonation Rates(Rework)
ka = 10^(-6);
Ka = 10^(-pKasystem); %Product ka*CNiII*HA should equal 1e1 roughly
kb = ka / Ka;


  % Variables
    CNiII   = C(1);
    CNiIISH = C(2);
    CNiISH  = C(3);
    CNiIIIH = C(4);
    CNiIIH  = C(5);


   % Rate Laws / ODEs
    dNiII   = -ka*CNiII*HA + kb*CNiIISH*A + k2*CNiIIH*HA;
    dNiIISH = -kb*CNiIISH*A + ka*CNiII*HA - kred(t, x)*CNiIISH + kox(t, x)*CNiISH;
    dNiISH  = kred(t, x)*CNiIISH - kox(t, x)*CNiISH - k1*CNiISH;
    dNiIIIH = k1*CNiISH - kred(t, x)*CNiIIIH + kox(t, x)*CNiIIH;
```

```
    dNiIIH  = kred(t, x)*CNiIIIH - kox(t, x)*CNiIIH - k2*CNiIIH*HA;
    de      = -kred(t, x)*(CNiIISH + CNiIIIH) + kox(t, x)*(CNiISH + CNiIIH);

  % Assign Output Variables
    dC(1,:) = dNiII;
    dC(2,:) = dNiIISH;
    dC(3,:) = dNiISH;
    dC(4,:) = dNiIIIH;
    dC(5,:) = dNiIIH;
    dC(6,:) = de;


end
```

Representative simulations overlaid with experimental values for identifying best-fit parameters

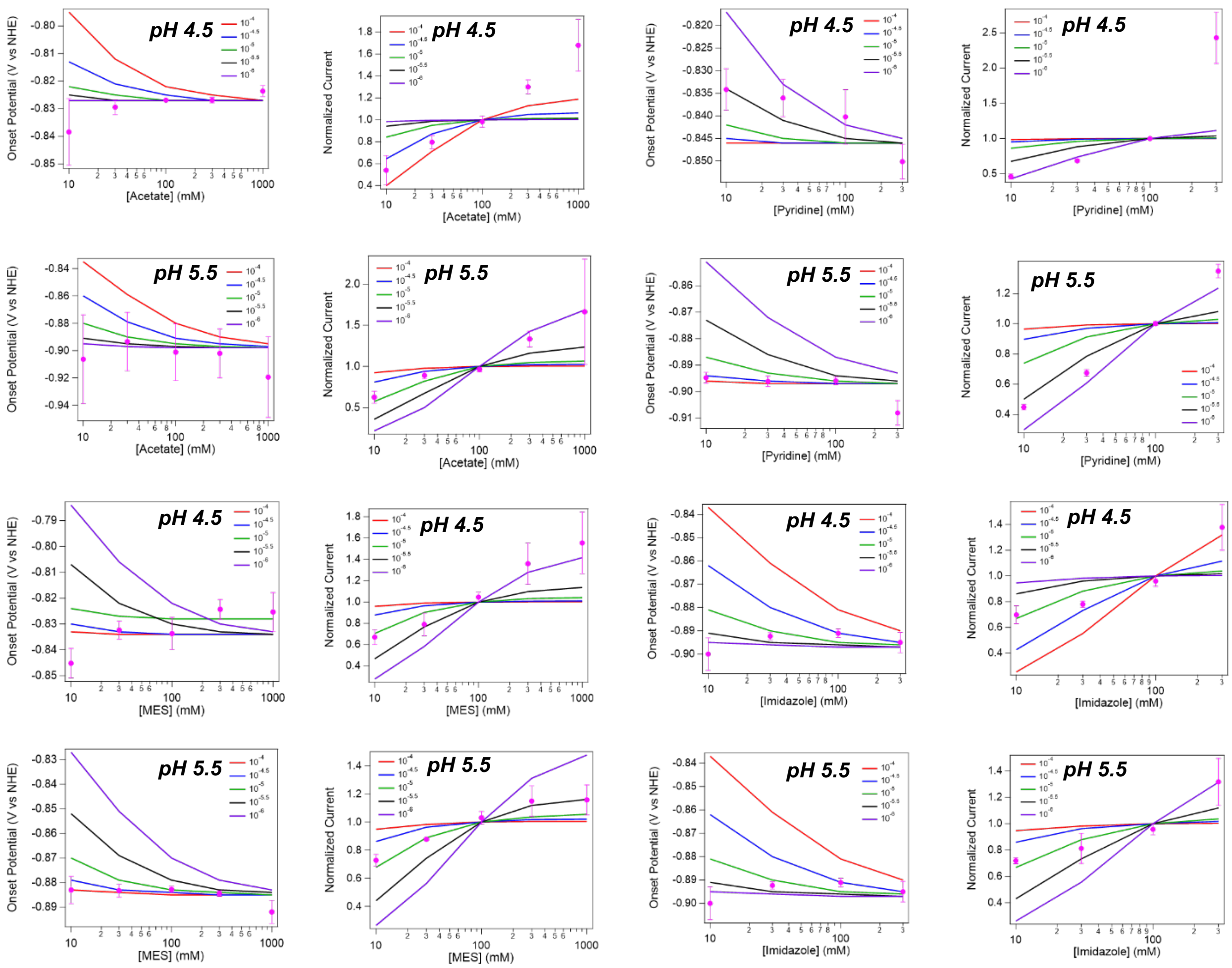

To fit the k_a value for each buffer, both the onset potential and normalized current values were modeled at both pH 4.5 and 5.5. All other parameters were kept constant as k_a was varied. All concentrations were simulated in the nM range to avoid integration artifacts and therefore rate constants listed are $nM^{-1}\ s^{-1}$.

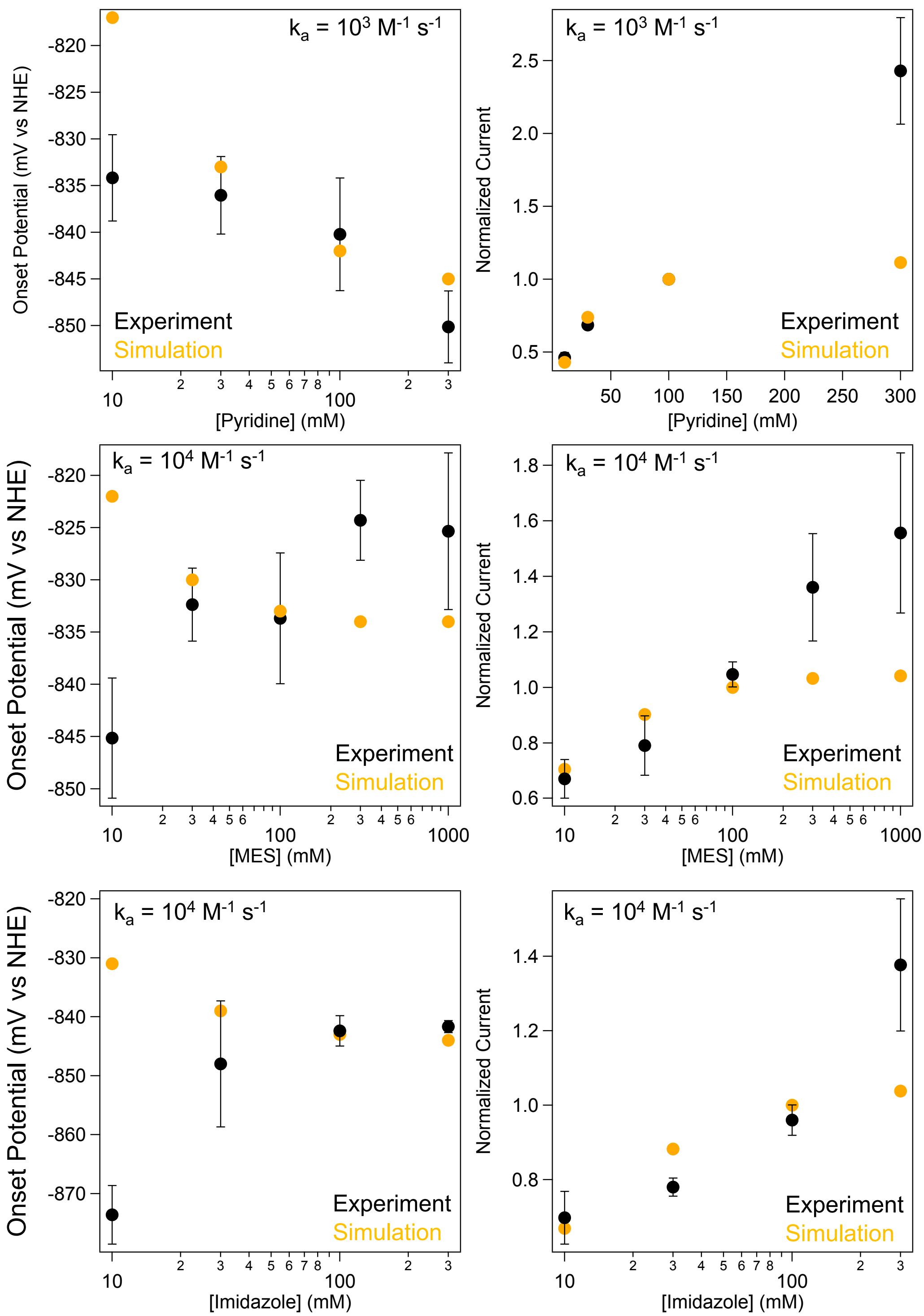

Figure S39. Simulated and experimental onset potential (left) and normalized currents (right) given best-fit k_a values (indicated on figure) for pyridine, MES, and imidazole.

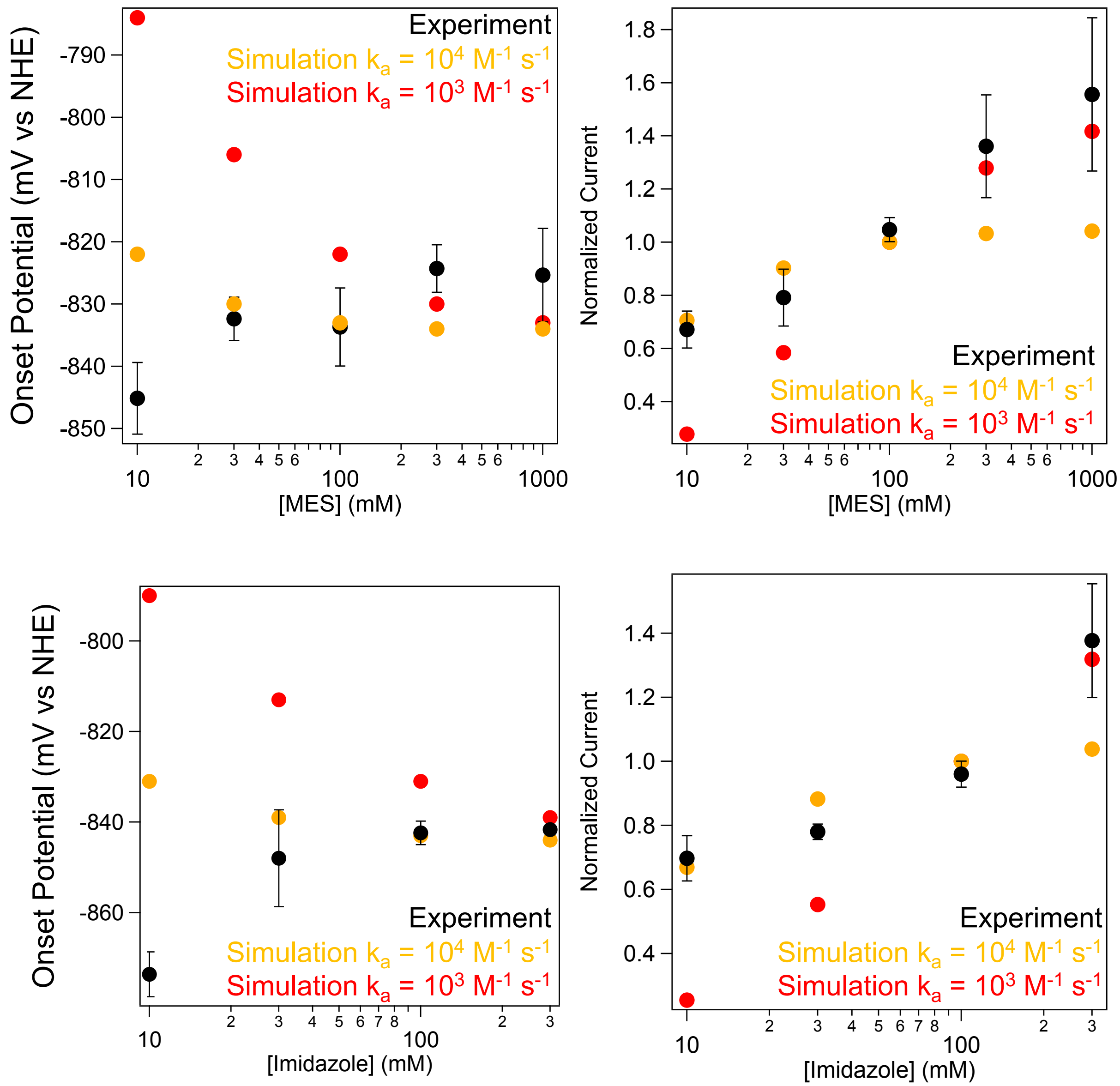

Figure S40. Simulated and experimental onset potential (left) and normalized currents (right) given best-fit k_a values and ka values constrained to the acetate value (k_a = 10^3 M^{-1} s^{-1}) (indicated on figure) for MES and imidazole.

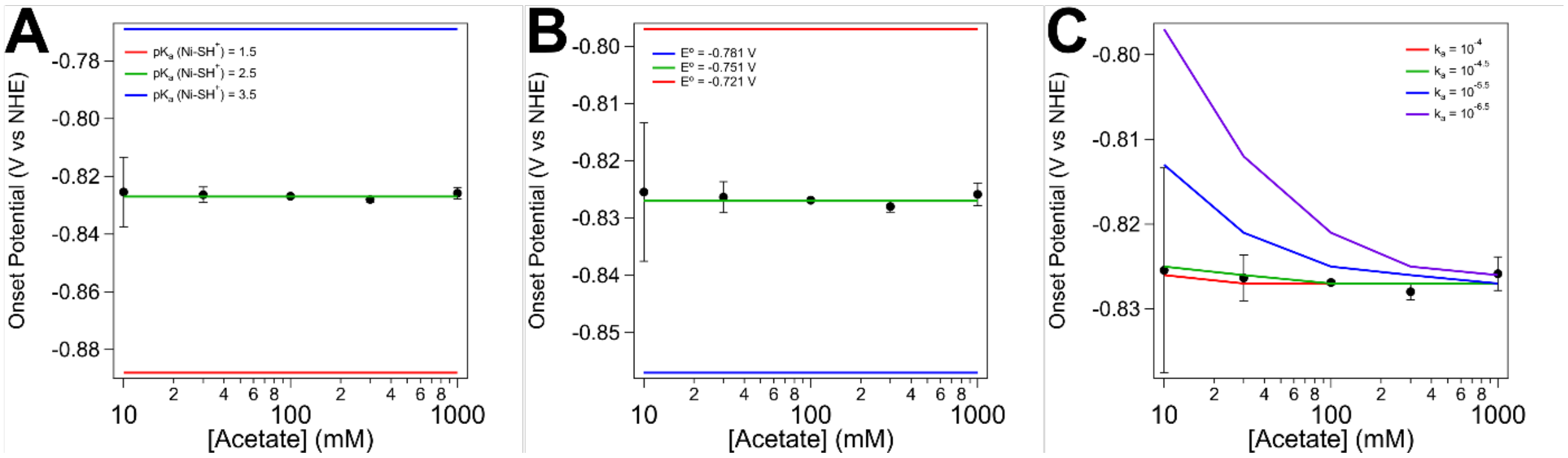

Figure S41. Sensitivity of mechanistic parameters. (A) Sensitivity of simulated onset potential with the pK_a of the nickel-thiolate ($Ni\text{-}SH^+$) with E° = -0.751 V vs NHE and $k_a = 10^{-4}$. (B) Sensitivity of simulated onset potential with the intrinsic E° with pK_a ($Ni\text{-}SH^+$) = 2.5 and ka = 10^{-4}. (C) Simulated onset potential with E° = -0.846 V and pK_a ($Ni\text{-}SH^+$) = 4.5. In all graphs, the experimental data for acetate at pH 4.5 is plotted in black and the various changed parameters are shown as connected points in blue, green, and red. All potentials in the caption are against NHE. The intrinsic E° of the system is essentially a standard reduction potential for the nickel center. Thus, the experimental onset potential differs from the intrinsic E° in a Nernstian way. In the simulations, the onset potential can be changed by adjusting the pK_a of the $Ni\text{-}SH^+$ which directly affects the ratio of Ni^{II}/ $Ni\text{-}SH^+$. Alternatively, the k_a can achieve a similar effect because it is related to the pK_a of the system. k_a has the largest impact on any buffer dependence on onset potential, while the pK_a of $Ni\text{-}SH^+$ and E° only impact the value of the observed onset potential. As observed in (A)-(B), the experimental data could be reproduced using a less basic nickel-thiolate and negative onset potential, but this fails to reproduce the pH dependence of the onset potential, as shown in Ref. 3. More basic pK_a values are unlikely given that experimental attempts to observe a protonated thiol have not been successful at pH ≥ 3.

Supplemental References

(1) Fourmond, V. QSoas: A Versatile Software for Data Analysis. *Anal. Chem.* **2016**, *88* (10), 5050–5052. https://doi.org/10.1021/acs.analchem.6b00224.
(2) Espinoza, E. M.; Clark, J. A.; Soliman, J.; Derr, J. B.; Morales, M.; Vullev, V. I. Practical Aspects of Cyclic Voltammetry: How to Estimate Reduction Potentials When Irreversibility Prevails. *J. Electrochem. Soc.* **2019**, *166* (5), H3175. https://doi.org/10.1149/2.0241905jes.
(3) Slater, J. W.; Marguet, S. C.; Monaco, H. A.; Shafaat, H. S. Going beyond Structure: Nickel-Substituted Rubredoxin as a Mechanistic Model for the [NiFe] Hydrogenases. *J. Am. Chem. Soc.* **2018**, *140* (32), 10250–10262. https://doi.org/10.1021/jacs.8b05194.
(4) Treviño, R. E.; Slater, J. W.; Shafaat, H. S. Robust Carbon-Based Electrodes for Hydrogen Evolution through Site-Selective Covalent Attachment of an Artificial Metalloenzyme. *ACS Appl. Energy Mater.* **2020**, *3* (11), 11099–11112. https://doi.org/10.1021/acsaem.0c02069.
(5) Fourmond, V.; Lautier, T.; Baffert, C.; Leroux, F.; Liebgott, P.-P.; Dementin, S.; Rousset, M.; Arnoux, P.; Pignol, D.; Meynial-Salles, I.; Soucaille, P.; Bertrand, P.; Léger, C. Correcting for Electrocatalyst Desorption and Inactivation in Chronoamperometry Experiments. *Anal. Chem.* **2009**, *81* (8), 2962–2968. https://doi.org/10.1021/ac8025702.
(6) Yan, Y.; Zeitler, E. L.; Gu, J.; Hu, Y.; Bocarsly, A. B. Electrochemistry of Aqueous Pyridinium: Exploration of a Key Aspect of Electrocatalytic Reduction of CO2 to Methanol. *J. Am. Chem. Soc.* **2013**, *135* (38), 14020–14023. https://doi.org/10.1021/ja4064052.
(7) Wertz, A. E.; Teptarakulkarn, P.; Stein, R. E.; Moore, P. J.; Shafaat, H. S. Rubredoxin Protein Scaffolds Sourced from Diverse Environmental Niches as an Artificial Hydrogenase Platform. *Biochemistry* **2023**, *62* (17), 2622–2631. https://doi.org/10.1021/acs.biochem.3c00249.
